# Discovery of core genes for systemic lupus erythematosus via genome-wide aggregated *trans*-effects analysis

**DOI:** 10.1038/s41435-025-00352-4

**Published:** 2025-09-03

**Authors:** Andrii Iakovliev, Olivia Castellini-Pérez, Buddhiprabha Erabadda, PRECISESADS Clinical Consortium, Javier Martín, Guillermo Barturen, Elena Carnero-Montoro, Marta E. Alarcón-Riquelme, PRECISESADS Flow Cytometry Consortium, Javier Martín, Guillermo Barturen, Elena Carnero-Montoro, Marta E. Alarcón-Riquelme, Javier Martín, Guillermo Barturen, Paul M. McKeigue, Elena Carnero-Montoro, Marta E. Alarcón-Riquelme, Athina Spiliopoulou

**Affiliations:** 1 Institute of Genetics and Cancer, College of Medicine and Veterinary Medicine, University of Edinburgh, Edinburgh, Scotland UK; 2 GENYO. Pfizer-University of Granada-Junta de Andalucía Centre for Genomics and Oncological Research, Granada, Spain; 3 University of Granada, Granada, Spain; 4 Usher Institute, College of Medicine and Veterinary Medicine, University of Edinburgh, Edinburgh, Scotland UK; 5 IBPLN-CSIC, Instituto de Parasitología y Biomedicina “López-Neyra” (CSIC), Granada, Spain; 6 Department of Genetics, Faculty of Sciences, University of Granada, Granada, Spain; 7 Unit of Chronic Inflammation, Institute for Environmental Medicine, Karolinska Institutet, Stockholm, Sweden

**Keywords:** Disease genetics, Genome-wide association studies, Autoimmunity, Chronic inflammation

## Abstract

The “omnigenic” hypothesis postulates that the polygenic effects of common variants on a typical complex trait coalesce on relatively few core genes through *trans*-effects on their expression. Our aim was to identify core genes for systemic lupus erythematosus (SLE) by testing for association with genome-wide aggregated *trans*-effects (GATE) scores for gene expression in a large genetic dataset (5267/4909 SLE cases/controls). SLE was strongly associated with upregulation of expression of eight interferon-stimulated genes driven by shared *trans*-effects. We estimate that *trans*-effects on interferon signaling account for 9% of the total genetic effect on SLE risk. Outside this pathway, GATE analysis detected twenty putative core genes for SLE. Direct protein measurements for these genes were strongly associated with SLE in UK Biobank. Two putative core genes (*TNFRSF17*, *TNFRSF13B*) encode receptors (BCMA, TACI) expressed on B cells; their ligands (BAFF, APRIL) are targeted by drugs licensed or in development for SLE. Four genes (*PDCD1*, *LAG3*, *TNFRSF9*, *CD27*) encode receptors that have been characterized as immune checkpoints, and three (*CD5L*, *SIGLEC1*, *CXCL13*) are biomarkers of SLE disease activity. These results provide genetic support for existing drug targets in SLE (interferon signaling, BAFF/APRIL signaling) and identify other possible therapeutic targets including immune checkpoint receptors.

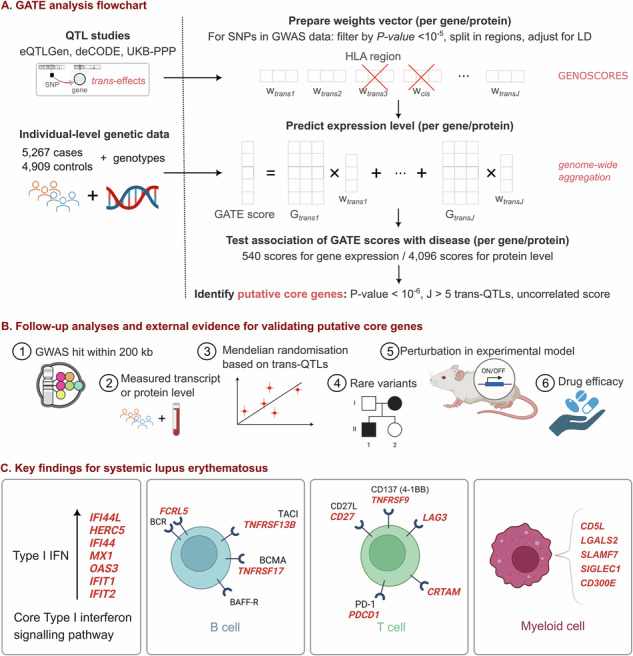

## Introduction

Systemic lupus erythematosus (SLE) is a chronic autoimmune disease that can affect almost any organ or tissue resulting in a wide range of clinical presentations. The disease affects mainly younger women; it is ranked among the top 10 leading causes of death in Black and Hispanic women aged 15-44 years in the US [1]. SLE is characterized by loss of immune tolerance to nuclear antigens, evidenced by the presence of antinuclear antibodies [2]. Defective clearance of remnants from cell death during apoptosis or in neutrophil extracellular traps leads to accumulation of nuclear autoantigens that trigger the innate and adaptive branches of the immune system through production of type I interferons and inflammatory cytokines [3, 4].

The total genetic contribution to disease is typically measured by the recurrence risk ratio in first-degree relatives, which for SLE is estimated as 16 [5, 6]. The strongest genetic association for SLE is with the HLA-DRB1*03:01 allele in the human leukocyte antigen (HLA) region, which has an odds ratio of 1.87 [7]. This allele can be shown to account for about 1% of the total genetic effect on the risk of SLE (see Supplementary Methods). Outside the HLA, approximately 200 genomic regions in which common variants are associated with SLE have been identified through Genome-Wide Association Studies (GWAS) [8]. However, as for other complex diseases, the genes nearest to disease-associated variants are often expressed broadly across tissues, and the contribution of GWAS findings to drug target discovery has been limited.

The “omnigenic model”, renamed by us as the “sparse effector hypothesis” [9], postulates that the polygenic effects of common variants on disease coalesce on a relatively sparse set of core genes via long-range *trans*-effects [10, 11]. On this hypothesis, while there may be thousands of peripheral genes through which common variants influence disease via *cis*-effects on their expression, there may be only a few dozen *core genes*, expressed in disease-relevant tissues, in the effector layer. Identifying these core genes is a promising strategy for drug target identification. Modification of the product of core genes would be more closely related to disease impact, while intervention on the activity of peripheral genes, which are further away in the regulatory network, would have weaker downstream effects on disease and higher likelihood of inducing side effects by also disrupting other regulatory processes.

Until recently it was not possible to test the sparse effector hypothesis directly because *trans*-effects on gene expression are mostly weak, and very large studies are required to detect them. With the availability of summary statistics based on more than 30,000 individuals for associations of single-nucleotide polymorphisms (SNPs) with transcript levels in eQTLGen [12], and with circulating protein levels in the deCODE [13] and UK Biobank studies [14], it has become possible to construct polygenic scores for gene products based on aggregating *trans*-effects of quantitative trait loci for gene expression (*trans*-eQTLs) and for protein level (*trans*-pQTLs). We have described the application of this Genome-wide Aggregated *Trans*-Effects (GATE) analysis to type 1 diabetes [9]. In this study, we apply GATE analysis to detect putative core genes for SLE and describe a systematic approach for gathering additional evidence in support of a direct causal role of candidate genes in SLE based on six independent criteria.

## Materials and methods

### Genetic analysis

We combined individual-level data from three case-control studies of the genetics of SLE: the BIOLUPUS/HRS study [15], the SLEGEN Consortium [16], and the PRECISESADS study [17]. Details for the component studies are given in Supplementary Methods. The three studies were combined into a single dataset after excluding variants with minor allele frequency below 5%. Quality controls were performed to exclude duplicated individuals, related individuals with proportion of identity-by-state above 0.5 (PLINK PI_HAT value), and individuals with a mismatch between reported and genetically inferred sex. The final dataset comprised 10176 individuals of European ancestry (5267 cases and 4909 controls). Genetic principal components were computed using the variance-standardized relationship matrix in PLINK.

Ethical approval for the component studies was obtained from the local ethical committees at each clinical recruitment site [15–17]. All participants were adults aged 18 or older, and each provided written informed consent. Additional ethical approval for the analyses described in this article was obtained from the University of Edinburgh research ethics committee (REC reference 24-EMREC-041). All methods were performed in accordance with the relevant guidelines and regulations.

Replication of findings from the GATE analysis in this discovery dataset was sought in the UK Biobank (UKB), a cohort of 502,166 participants resident in Britain, aged between 40 and 69 at recruitment. SLE case-control status was derived from first mention of this diagnosis (field ID 131895) in hospital diagnoses, primary care diagnoses, or self-reports. Of 1101 identified SLE cases, 252 were based on self-report only. The remaining individuals were assigned as controls. GATE replication analysis was restricted to unrelated individuals of European ancestry, resulting in 907 SLE cases and 450,547 controls. Written consent was obtained from all UKB participants. Ethical approval for the UKB resource was granted in 2011 by the North West Multi-centre Research Ethics Committee (11/NW/0382), and renewed every 5 years. The work described in this article has been approved by UKB (application number 23652).

### Genome-wide aggregated *trans*-effects (GATE) analysis

**Fig. 1:**
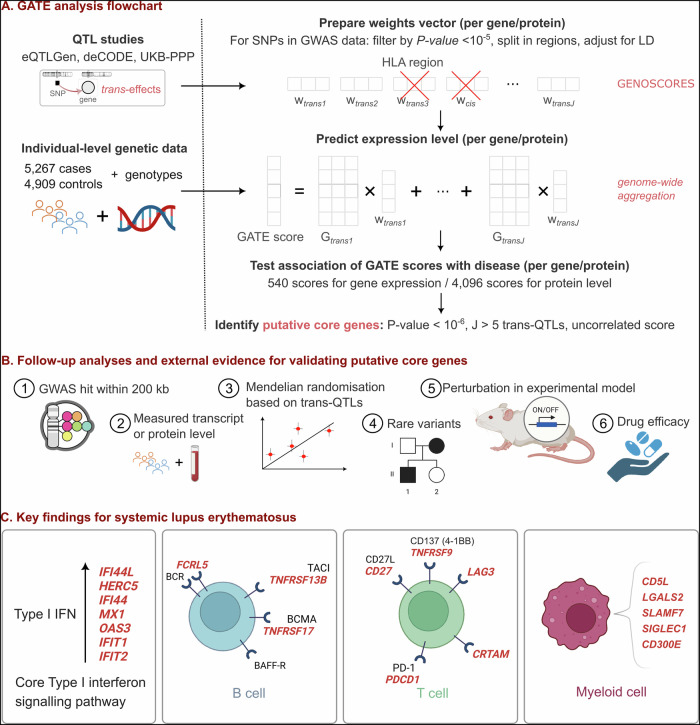
Study overview. **A** Flowchart of Genome-wide Aggregated *Trans*-Effects (GATE) analysis. **B** Summary of follow-up analyses and external evidence for validating putative core genes. **C** Overview of key findings from application to systemic lupus erythematosus (SLE). Parts of this image were created in BioRender. Iakovliev, A. (2024), https://BioRender.com/d04z288.

Methods for GATE analysis have been described previously [9]. We provide a summary of the analytical steps (Fig. 1A), with more details given in Supplementary Methods.

Summary statistics for *trans*-QTLs were extracted from three GWAS of whole-blood gene expression or circulating protein levels in large general population cohorts: the eQTLGen Consortium [12], the deCODE SomaScan study [13], and the UK Biobank Pharma Proteomics Project (UKB-PPP) [14].

A genotypic score for each gene and each protein was computed for each individual in the SLE case-control datasets by adding up the individual’s genotypes across all *trans*-QTLs for that gene/protein weighted by the corresponding effect sizes. The GENOSCORES platform was used for the score computation [18], which included splitting SNPs that were imputed in the case-control dataset into QTLs, classifying these as *cis* or *trans* and adjusting the SNP effect sizes within each QTL for linkage disequilibrium.

*Trans*-QTLs at the HLA region (from 25 to 34 Mb on chromosome 6) were excluded from the computation of GATE scores to avoid confounding of the association of a GATE score with SLE by the strong direct effects of HLA antigens on SLE. HLA-specific *trans*-scores and *cis*-scores were tested for association with SLE separately. GATE scores with fewer than 5 *trans*-QTLs were removed, on the basis that aggregating multiple *trans*-QTLs increases the “signal” (effects from multiple *trans*-QTLs consistent in direction) to “noise” (effects random in direction) ratio, and limits confounding of associations by strong effects from a single *trans*-QTL.

In total, 540 aggregated *trans*-scores for expression (eGATE), corresponding to 540 unique genes, and 4096 *trans*-scores for protein levels (pGATE), corresponding to 3295 unique genes, were tested for association with SLE. A logistic regression model was used, adjusting for sex, cohort, and the first 10 genetic principal components in the discovery dataset (combined SLE-genetics studies), and for sex and the first 20 genetic principal components in the replication dataset (UKB).

Putative core genes were identified using a threshold of $p\, < \,{10}^{-6}$ for the association of SLE risk with an eGATE or pGATE score for a gene. Genes with correlated GATE scores were not assigned “putative core gene” status, as correlated scores have substantial sharing among their contributing *trans*-QTLs. In this case, it is not possible to distinguish which gene is mediating these shared *trans*-effects on disease. To further characterize the genomic locations harboring these shared signals, we grouped *trans*-QTLs into clumps if they were within 200 kb of each other.

An information theoretic approach was used to estimate the contribution of *trans*-effects on a gene or pathway to SLE risk. For a rare disease, under a model in which genetic effects on SLE are additive on a logistic scale, the total genetic information for discrimination (discriminating cases from non-cases) is equal to the logarithm of the sibling recurrence risk ratio [19, 20]. For SLE, this corresponds to 4 bits of information (${\log }_{2}\left(16\right)$) using logarithms to base 2. The contribution of a single genetic predictor to the information for discrimination (in natural log units) is half the square of the standardized log odds ratio for its association with the disease (${\beta }^{2}/2$) [20]. This can be converted to bits by dividing by $\log \left(2\right)$. To quantify the expected information for discrimination attributable to *trans*-effects on the expression of a gene we additionally accounted for the dilution of the underlying genetic effects due to the GATE score being an imperfect predictor. Specifically, we used:

1 $$I=\frac{{\beta }^{2}}{2\times {\text{log}}\left(2\right)}/\frac{{r}^{2}}{{h}_{trans}^{2}},$$

where ${r}^{2}$ is the squared correlation between GATE score and measured expression, ${h}_{trans}^{2}$ is the *trans*-heritability of expression, and the dilution factor, ${r}^{2}/{h}_{trans}^{2}$, is the proportion of variance of gene expression attributable to *trans*-heritability that is explained by the GATE score (see also Supplementary Methods).

GATE-detected putative core genes were followed up using six prespecified criteria to evaluate supportive evidence for their causal role in SLE (Fig. 1B and Supplementary Methods).

### Transcriptomics analysis

Whole-blood RNA samples from the PRECISESADS study [17] were sequenced on a HiSeq2500 device with the v4 SBS Kit (Illumina). Post-processing included gene-level expression quantification and application of quality controls described in Supplementary Methods. Whole-blood gene expression levels for 149 SLE cases and 219 healthy controls were analyzed. Normalization of gene expression levels was carried out using variance-stabilizing transformation (VST). The resulting distributions were moderately right skewed (Supplementary Figs. S1 and S2). A logistic regression model, which does not make assumptions about the distribution of the input variables, was used to test the association of each transcript with SLE case-control status, adjusting for sex, age, sample extraction center and cell type proportions. Cell type proportions were determined using a standardized flow cytometry workflow [21] to quantify the frequencies of CD15hiCD16hi neutrophils, CD15hiCD16+ eosinophils, CD14+CD15hi low density granulocytes (LDGs), CD14hiCD16- classic monocytes, CD14+/hiCD16+ intermediate monocytes, CD14-CD16+ non classic monocytes, CD3+ T cells, CD19+ B cells, CD3-CD56+ NK cells, CD3+CD56+ NK-like cells, Lin-HLA-DR+ dendritic cells, and CD123+HLA-DR- basophils as previously described [17].

### Proteomics analysis

The UKB-PPP measured 2923 circulating proteins in 54,219 individuals using the Olink Explore 3072 library. Cases of SLE were oversampled for the proteomics study, so that 557 cases were included [14]. The association between SLE case-control status and the measured protein level in the UKB-PPP dataset was tested using a logistic regression model adjusting for sex, age at assessment and self-reported racial background categorized as “White” or “Other”.

## Results

### Associations of aggregated *trans*-eQTL scores with lupus

GATE analysis using *trans*-effects on gene expression detected 8 genes with strong associations between their eGATE score and SLE risk (Table 1 top panel). These genes are recognizable as interferon-stimulated genes (ISGs) for type I interferon; their co-expression constitutes an “interferon signature”. The eGATE scores for all detected ISGs were also significantly associated with SLE in the UKB replication analysis (Supplementary Table S1).

**Table 1:** Candidate core genes for SLE based on aggregated *trans*-effects on gene expression (top panel; eGATE) or on protein levels (bottom panel; pGATE).

Gene symbol	Transcription site		GATE score			*cis*-score		GWAS hit within 200 kb
	Chr	Start position (Mb)	N *trans-*loci	Log OR	*P*	Log OR	*P*	
*Trans*-effects on transcript levels								
*(RSAD2)*	2	6.87	6	0.23	3×10^-22^	0	0.9	
*(IFI44L)*	1	78.62	8	0.17	4×10^-12^	−0.03	0.2	
*(HERC5)*	4	88.46	6	0.16	6×10^-11^			
*(IFI44)*	1	78.65	6	0.16	6×10^-11^	−0.02	0.3	
*(MX1)*	21	41.42	7	0.16	7×10^-11^	−0.03	0.2	
*(OAS3)*	12	112.94	5	0.15	1×10^-10^	0.03	0.3	+
*(IFIT1)*	10	89.39	6	0.15	2×10^-10^	0	0.9	
*(IFIT2)*	10	89.28	5	0.15	3×10^-10^	0.03	0.2	
*Trans*-effects on protein levels								
*CD27*	12	6.44	36	0.18	1×10^-13^	0.01	0.7	
*TNFRSF17*	16	11.97	46	0.18	1×10^-13^	−0.04	0.06	
*CD5L*	1	157.83	64	0.18	2×10^-13^	−0.05	0.04	*FCRL5*
*LGALS2*	22	37.57	10	0.16	1×10^-10^	0.02	0.4	
*CXCL10*	4	76.02	18	0.15	7×10^-10^	−0.02	0.3	
*CRTAM*	11	122.84	13	0.14	2×10^-9^	0.02	0.3	
*CXCL13*	4	77.51	9	0.15	3×10^-9^	−0.01	0.5	
*TNFRSF13B*	17	16.93	30	0.14	5×10^-9^	-0.06	0.007	*TNFRSF13B*
*TNFRSF9*	1	7.92	21	0.14	1×10^-8^	−0.01	0.7	
*MX1*	21	41.42	10	0.14	1×10^-8^	−0.04	0.08	
*SLAMF7*	1	160.74	29	0.13	2×10^-8^	−0.04	0.06	
*SIGLEC1*	20	3.69	29	0.13	4×10^-8^	0.04	0.1	
*ZG16*	16	29.78	5	0.13	6×10^-8^			
*ALKBH2*	12	109.09	6	0.13	6×10^-8^	0	1	
*IL5RA*	3	3.07	31	0.12	2×10^-7^	−0.03	0.2	
*FCRL5*	1	157.51	42	0.12	3×10^-7^	−0.03	0.2	*FCRL5*
*PDCD1*	2	241.85	38	0.12	4×10^-7^	0	0.9	
*LAG3*	12	6.77	20	0.12	5×10^-7^	−0.02	0.4	
*SHISA5*	3	48.47	11	0.12	6×10^-7^	0.02	0.3	
*CD300E*	17	74.61	28	0.12	7×10^-7^	−0.01	0.6	

Genes in this table are those with GATE scores associated with SLE at *p* < 10^−6^, and with number of trans-QTLs ≥ 5.

Gene symbols in brackets represent a cluster of interferon stimulated genes (ISGs) with highly correlated GATE scores.

Association with the *cis*-score for a gene is presented when at least one *cis*-SNP with *p* < 10^−6^ has been reported in the eQTL/pQTL study. None of these genes would be detected by looking at *cis*-effects on gene expression/protein level.

If a candidate core gene is within 200 kb of a known GWAS hit, and the GWAS hit has been attributed to a gene by the original study, then the reported gene symbol is displayed. If the GWAS hit has not been attributed to a gene, then the “+” sign is displayed.

The full name and the aliases of the proteins encoded by putative core genes shown in the bottom panel (pGATE) are given in Supplementary Table S6.

*Chr* chromosome, *Mb* Megabase, *Log OR* log(odds ratio) for one standard deviation change in the score, *P* *p*-value of association.

**Fig. 2:**
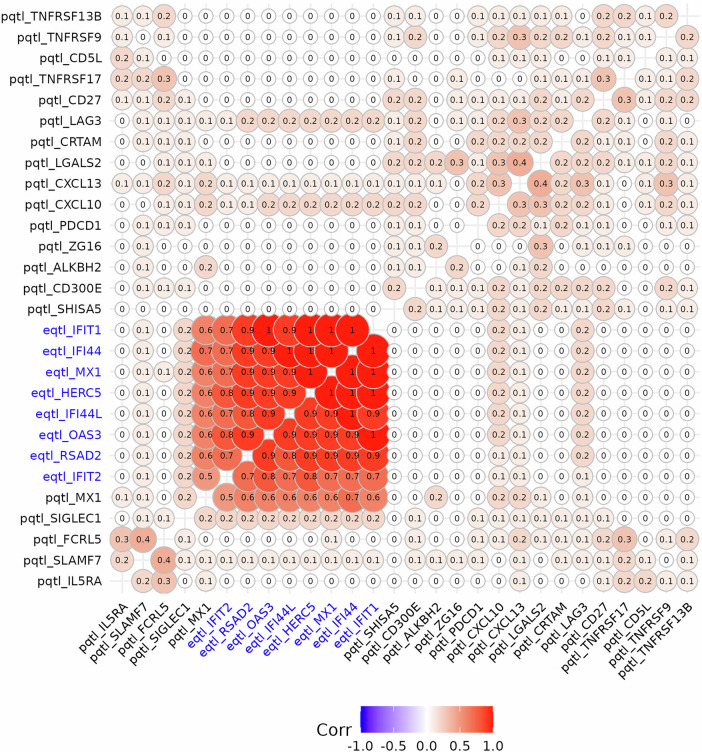
Heatmap of correlations among GATE scores for genes detected by GATE analysis ordered by hierarchical clustering. Correlations were computed using SLE controls in the discovery dataset. The three-letter prefix, *eqtl* or *pqtl* denotes if the corresponding gene was detected via aggregated *trans*-effects on expression or protein level, respectively. Blue labels are used to highlight the cluster of interferon stimulated genes (ISGs) with highly correlated GATE scores.

**Fig. 3:**
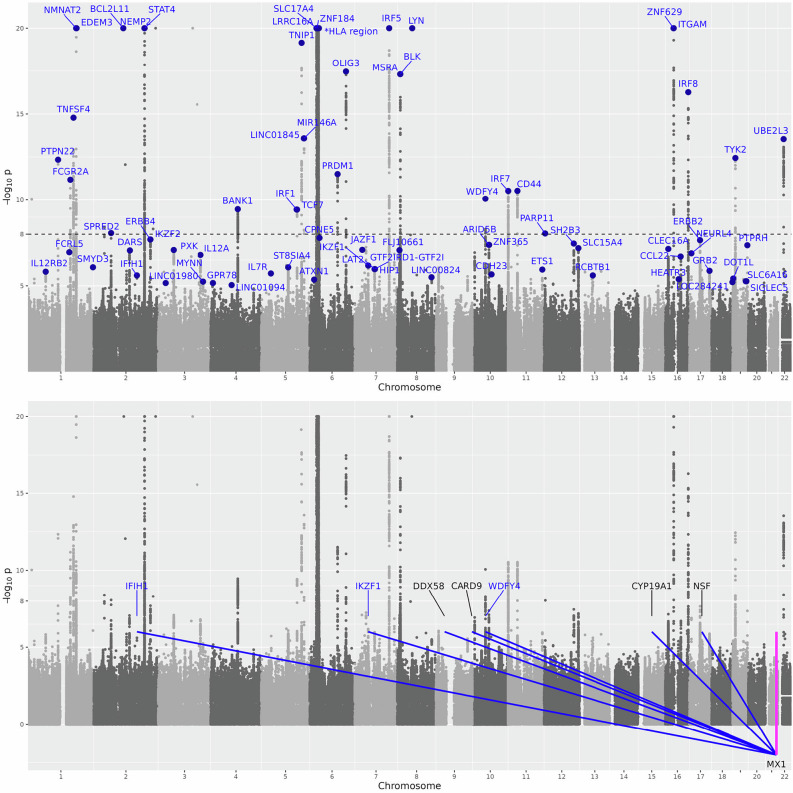
Manhattan plot for the association of genetic variants with SLE using summary statistics from Bentham et al. [15] annotated with known SLE GWAS hits from the GWAS Catalog (top) and with eQTLs for *MX1* gene expression (bottom). Blue lines point to *trans*-eQTLs for *MX1*. Magenta line points to the *cis*-eQTL.

The GATE scores for all detected ISGs were highly correlated with each other (Fig. 2), explained by shared *trans*-effects on their expression. Specifically, after clumping by genomic location, most genetic contribution comes from 5 shared *trans*-eQTLs (Fig. S3). Four of these are in the *IFIH1*, *RIGI* (also known as *DDX58*), *IKZF1*, and *WDFY4* regions, previously reported as genes in which rare variants cause monogenic lupus [22, 23] or for harboring SLE GWAS variants [15, 24] (Fig. 3). Association of SLE with these GATE scores independently replicates the convergence of SLE-associated variants on this cluster of ISGs, as previously described [12, 25].

**Fig. 4:**
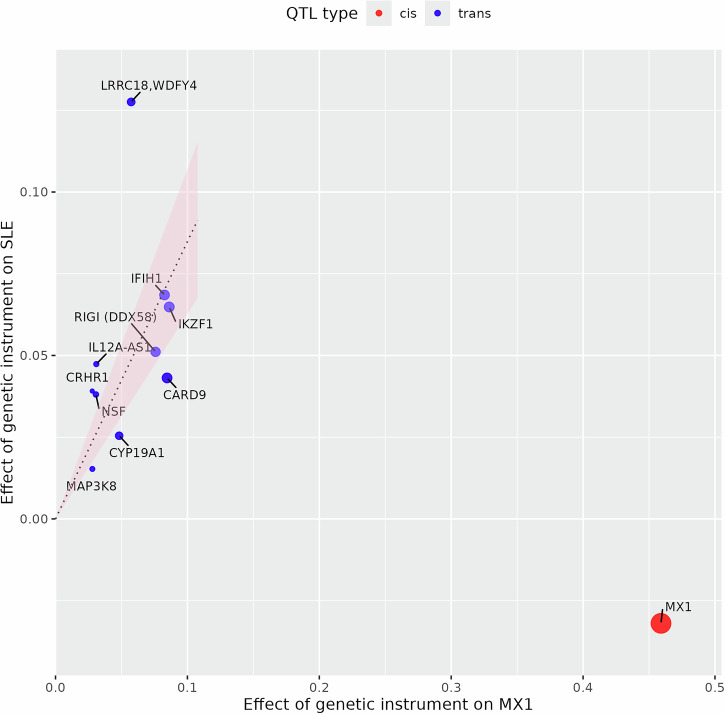
Effects of individual eQTLs for *MX1*, on *MX1* expression level (x-axis) and on SLE risk (y-axis). Blue points show effects of *trans*-eQTLs, labeled by nearby genes. Red point shows effects of the *cis*-eQTL. The size of the marker is inversely proportional to the standard error of the Wald ratio estimate for the effect of *MX1* expression level on SLE based on each individual eQTL. The slope of the line is the causal effect estimate of *MX1* expression (exposure) on SLE risk (outcome) using all *trans*-eQTLs as instrumental variables. The red shaded area contains all possible lines passing through the origin whose slope is within one standard error of the estimate. Estimation is performed by marginalizing over the posterior distribution of (unobserved) pleiotropic effects of the *trans*-eQTLs. *IFIH1*, *IKZF1* and *WDFY4* are known GWAS hits for SLE. Rare variants in *IFIH1*, *IKZF1* and *RIGI* (also known as *DDX58*) are known to cause monogenic forms of lupus.

The independent effects of these *trans*-eQTLs on ISG expression and on SLE risk are consistent in direction and roughly proportional, albeit with some scattering, as shown for *MX1* (Fig. 4). Accordingly, all detected ISGs were supported at $p\, < \,0.01$ by Mendelian randomization using *trans*-eQTLs as instrumental variables (Supplementary Table S2). However, since the majority of these *trans*-eQTLs are shared across multiple ISGs, it is not possible to distinguish individual ISGs as causal without additional evidence.

The *cis*-scores for *MX1* and the other detected ISGs are not associated with SLE (Table 1, Fig. 4). This could reflect *cis*-effects leading to changes in measured gene expression level but not in a manner relevant to disease, for instance, an alternatively spliced isoform without a downstream functional effect.

### Associations of aggregated *trans*-pQTL scores with lupus

GATE analysis using *trans*-effects on circulating protein levels detected 20 putative core genes for SLE based on the strength of association of their pGATE score with SLE (Table 1 bottom panel) and zero or low correlation among pGATE scores (Fig. 2).

Upon examining the genomic locations affecting these genes in *trans*, we observe that 58 (out of a total of 303) *trans*-pQTL clumps are located within 200 kb of a previously reported GWAS hit for SLE (Supplementary Fig. S4). This supports the omnigenic model for the genetic architecture of SLE, demonstrating that the effects of common variants on disease coalesce onto a smaller set of core genes through *trans*-effects on their expression. Of interest, another 15 *trans*-pQTL clumps are located within 200 kb of a gene in which rare variants cause monogenic lupus (Supplementary Fig. S4).

While 19 of the 20 putative core genes had a *cis*-score computed (at least one *cis*-SNP with $p\, < \,{10}^{-6}$), these *cis*-scores were not strongly associated with SLE (Table 1). On the other hand, 3 of the putative core genes were within 200 kb of a known GWAS hit for SLE based on data from the GWAS catalog [26]. The effect of these GWAS hits on the risk of disease could be through a *cis*-effect on the expression of the gene in a specific disease-relevant tissue or cell state.

Nine putative core genes were supported at $p\, < \,0.01$ by Mendelian randomization analysis (Supplementary Table S2), three reached nominal significance at $p\, < \,0.05$ in the UKB replication analysis, and all had consistent direction of effect on SLE between discovery and replication datasets (Supplementary Table S1). We note that the pGATE replication analysis in UKB had limited sample size (only about 350 SLE cases), because we excluded individuals in the UKB-PPP subset who had been used to estimate the pQTL effects.

### Associations of HLA scores with lupus

A total of 2941 HLA-specific scores for 239 genes and 2702 proteins were computed based on *trans*-effects from SNPs in the HLA region. Of these, 1032 scores were associated with SLE at $p\, < \,{10}^{-6}$ (Table S3). The HLA region is a hotspot for *trans*-eQTLs for genes involved in the immune system [27]. As in our previous GATE analyses of type 1 diabetes [9] and of rheumatoid arthritis [28], the HLA region was excluded from the aggregation of *trans*-effects into GATE scores to avoid confounding of associations between GATE scores and SLE by the strong direct effects of the HLA antigens on SLE risk. This exclusion is necessary to ensure that relevant genes can be detected over the many other genes that are simply affected by this *trans*-QTL hotspot.

### Associations of lupus with transcripts and proteins encoded by putative core genes

**Fig. 5:**
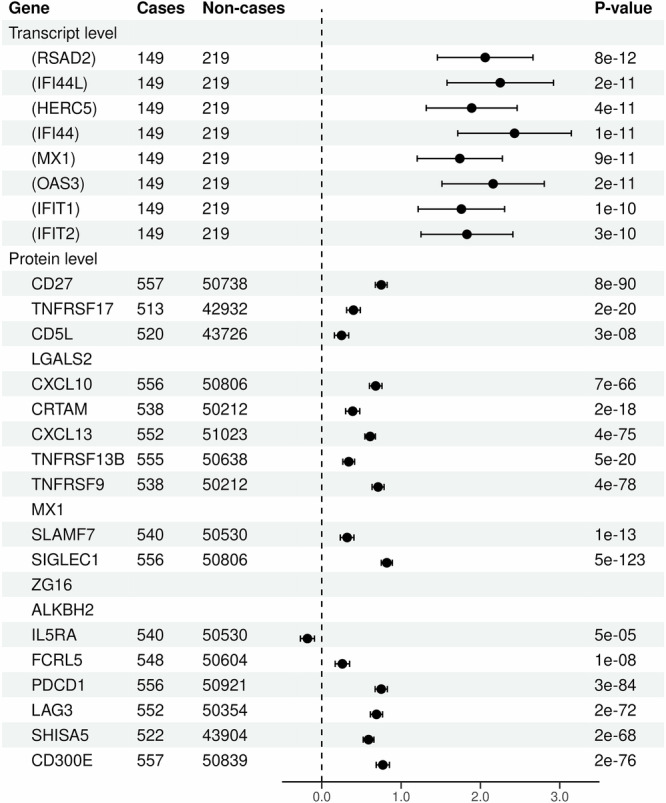
Forest plot for the associations of SLE with measured transcript levels (in PRECISESADS) for genes detected by eGATE analysis, and with measured protein levels (in UKB-PPP) for genes detected by pGATE analysis. For each gene the log(odds ratio) and 95% confidence interval corresponding to one standard deviation change in the measured transcript/protein level is shown. Gene symbols in brackets represent a cluster of interferon stimulated genes (ISGs) with highly correlated GATE scores. *LGALS2*, *MX1*, *ZG16*, and *ALKBH2* were not measured in UKB-PPP and could not be evaluated in this analysis.

All ISGs detected by the eGATE analysis had whole-blood RNAseq measurements in PRECISESADS [17], and all were statistically significantly upregulated in SLE cases compared to controls (Fig. 5). These findings are consistent with the known role of upregulation of ISGs in SLE. Additionally, we quantified the genetic contribution to SLE attributable to *trans*-effects on the interferon signaling pathway as 0.35 bits (Supplementary Table S4). This corresponds to 9% of the total genetic effect on SLE risk (4 bits) and is comparable to the effect of the HLA region, where the HLA-DRB1*03:01 allele –the strongest risk allele for SLE– explains 1% of the total genetic effect (0.048 bits).

Of the 20 putative core genes detected by pGATE analysis, 17 had measured protein levels in the UKB-PPP dataset [14]. All were strongly associated with SLE ($p\, < \,5\times {10}^{-5}$) and 15 had standardized effect sizes at least twice as large as the standardized effect size of the GATE score on SLE (Fig. 5 and Supplementary Table S5). In addition, the measured transcript levels for eight pGATE-detected genes were associated with SLE at $p\, < \,0.01$ in PRECISESADS (Supplementary Table S5).

These associations of transcript and protein levels with SLE include people already diagnosed with the disease. This means that changes in measured levels could be influenced by risk factors not adjusted for in our models, the disease itself, or its treatment. This is in contrast to genetic associations which cannot be confounded by factors occurring during the lifecourse.

### Summary of additional supporting evidence

All eGATE-detected ISGs were supported by association of SLE with measured transcript levels in PRECISESADS, and by Mendelian randomization. *OAS3* was also supported by a GWAS hit within 200 kb of its transcription site that had not been attributed to a gene. *MX1* was supported by perturbation in an experimental model of lupus [29] (Table 2 and Supplementary Table S6). In addition, the type I interferon pathway is targeted by anifrolumab, a monoclonal antibody to type I interferon receptor, recently approved for SLE [30, 31].

**Table 2:** Additional evidence in support of GATE-detected genes as core genes for SLE.

Gene symbol	GWAS hit (a)	Measured gene product (b)	Mendelian randomization (c)	Experimental validation (d)	Drug efficacy (e)
Analysis based on *trans*-eQTLs					
*(RSAD2)*	.	+	+	.	.
*(IFI44L)*	.	+	+	.	.
*(HERC5)*	.	+	+	.	.
*(IFI44)*	.	+	+	.	.
*(MX1)*	.	+	+	+	.
*(OAS3)*	+	+	+	.	.
*(IFIT1)*	.	+	+	.	.
*(IFIT2)*	.	+	+	.	.
Analysis based on *trans*-pQTLs					
*CD27*	.	+	.	.	.
*TNFRSF17*	.	+	+	+	+
*CD5L*	+	.	+		.
*LGALS2*	.	.	.	.	.
*CXCL10*	.	+	+	.	.
*CRTAM*	.	+	.	.	.
*CXCL13*	.	+	+	.	.
*TNFRSF13B*	+	+	.	+	+
*TNFRSF9*	.	+	+	+	.
*SLAMF7*	.	+	.	.	.
*SIGLEC1*	.	+	.	.	.
*ZG16*	.	.	.	.	.
*ALKBH2*	.	.	.	.	.
*IL5RA*	.	.	+	.	.
*FCRL5*	+	+	+	+	.
*PDCD1*	.	+	.	+	.
*LAG3*	.	+	+		.
*SHISA5*	.	+	.	.	.
*CD300E*	.	+	.	.	.

Gene symbols in brackets represent a cluster of interferon stimulated genes (ISGs) with highly correlated GATE scores.

(a) Contains “+” if a known GWAS hit for SLE exists within 200 kb of the gene’s transcription site or if a *cis*-score for the expression of the gene is associated with SLE.

(b) Contains “+” if the measured transcript level (for eQTLs) or the measured protein level (for pQTLs) of the gene is associated with SLE.

(c) Contains “+” if Mendelian randomization based on *trans*-QTLs and marginalizing over the distribution of pleiotropic effects supports a causal effect for the gene on SLE.

(d) Contains “+” if there is evidence of causality through perturbation of the gene in an animal model of SLE.

(e) Contains “+” if an existing drug (either investigational or approved) that targets the protein encoded by the gene or its ligand/receptor has some clinical evidence of efficacy against the disease.

Fifteen pGATE-detected core genes (of the 17 with measured protein levels in UKB-PPP) were supported by an association of SLE with the measured protein, and nine genes were supported by Mendelian randomization analysis. Two genes (*TNFRSF13B*, *FCRL5*) were supported by GWAS hits within 200 kb of their transcription sites, which had been attributed to the same gene. For *LGALS2* there is indirect support as it is a ligand for CD14 [32], which is encoded by a GWAS hit for SLE. Five genes were supported by perturbation of the gene in an experimental model of lupus, and two genes were supported by clinical evidence of efficacy of the biologic drug belimumab in SLE [33] (Table 2 and Supplementary Table S6).

## Discussion

### Interferon signaling

The central role of type I interferon signaling in SLE is well-established, based on higher expression of ISGs in cases than in controls, monogenic forms of lupus caused by rare variants in genes that encode proteins in the interferon signaling pathway, and clinical experience that therapeutic administration of interferon alpha frequently causes antinuclear antibodies and occasionally causes clinical lupus [23, 34, 35].

Our results show that common variants in multiple genes coalesce on the interferon signaling pathway via *trans*-effects to upregulate the expression of ISGs and to increase risk of SLE. This provides independent replication of the previously reported *trans*-association between SLE risk variants and expression of this cluster of ISGs based on expression quantitative trait score (eQTS) analysis [12, 25]. In eQTS analysis, a polygenic risk score for the disease is tested for association with the measured levels of expression of each gene. These results are also consistent with earlier work suggesting that basal interferon $\alpha$ (IFN-$\alpha$) activity is a primary risk factor of SLE, and not simply the result of autoimmunity, by showing that high serum IFN-$\alpha$ is a heritable trait, clustering in families of SLE patients who have high IFN-$\alpha$ themselves and that this clustering could not be accounted for by autoantibody status [36].

All detected ISGs share the same five *trans*-eQTLs at the *IFIH1*, *RIGI* (also known as *DDX58*), *IKZF1*, *WDFY4*, and *CARD9* regions. These genes have been previously reported for harboring GWAS hits for SLE (*IFIH1*, *IKZF1*, *WDFY4*) [15, 24], or as genes in which rare variants cause monogenic lupus (*IFIH1*, *IKZF1*, *RIGI*) [22, 23]. *IFIH1* and *RIGI* encode cytosolic sensors of double-stranded RNA that initiate interferon signaling (MDA5 and RIG-I, respectively). Gain-of-function variants in *IFIH1* and *RIGI* cause monogenic lupus. *IKZF1* regulates the development and function of plasmacytoid dendritic cells [37], a cell type with specialized capacity for production of type I and type III interferons [38]. *CARD9* mediates the activation by C-type lectin receptors of nuclear factor $\kappa$B (NF-$\kappa$B) [39], which regulates a subset of ISGs [40]. The WDFY4 protein interacts with pattern recognition receptors, including MDA5, and enhances NF-$\kappa$B signaling, while a truncated WDFY4 isoform interacts with MDA5 in the cytosol and alters downstream type I interferon signaling [41].

### Other pathways

Nineteen putative core genes for SLE were detected by GATE scores for circulating proteins (excluding *MX1*, an ISG detected by both transcript-based and protein-based GATE analyses). Seventeen of these genes are in the immune system: six regulate T cells, three regulate B cells, five regulate myeloid cells, and three encode cytokines.

#### Genes regulating B cells

*TNFRSF17* and *TNFRSF13B* encode receptors BCMA and TACI, respectively. These are two of the three receptors expressed on the surface of B cells that recognize B-cell Activating Factor (BAFF), an immunostimulant cytokine strongly involved in SLE pathogenesis. BCMA and TACI also recognize the A proliferation-inducing ligand (APRIL). TACI haploinsufficiency was protective of lupus nephritis in a BAFF-driven lupus mouse model [42]. Disruption of TACI in the Nba2.Yaa lupus mouse model led to reduced disease, while disruption of BCMA led to increased disease, attributed to increased TACI signaling [43].

*FCRL5* is a regulator of B cell-mediated autoimmunity. Upregulation of FcRL5 in B cells was shown to exacerbate pathogenesis in the imiquimod-induced SLE-like mouse model [44].

#### Genes regulating T cells

*CD27*, *TNFRSF9*, *LAG3* and *PDCD1* encode immune checkpoint receptors (CD27, CD137 also known as 4-1BB, LAG-3, PD-1, respectively), broadly defined as receptors on immune cells that are exploited by cancer cells to escape immunity. The soluble isoforms of the proteins encoded by these genes appear to act as decoys for the cellular receptor, so that high levels of the soluble protein reduce the downregulation of autoimmune responses that is signaled through the cellular receptor [45]. An intronic SNP in *PDCD1* has been previously associated with SLE [46]. Mice without the *PDCD1* gene develop lupus-like disease [47], and systemic lupus is a rare adverse effect of PD-1 inhibitors. Deletion of 4-1BB accelerates induction of disease and increases severity in the MRL/lpr lupus mouse model [48].

*ZG16* has been shown to modulate the immune response in colorectal cancer by regulating the expression of PD-L1, the ligand for PD-1 [49].

*CRTAM* encodes a cell surface receptor expressed by T cells and NK cells and regulates pre-inflammatory cytokine production and cytotoxicity. Interaction with its ligand, Necl-2, which is expressed by antigen-presenting cells, supports formation of an immunological synapse and a targeted cytotoxic response [50].

#### Genes regulating myeloid cells

*CD5L*, also known as apoptosis inhibitor of macrophages, is primarily expressed by macrophages and regulates their survival, but its role in autoimmunity could also be through its association with proliferation of B cells and Th17 cells [51]. Loss of CD5L has been shown to convert non-pathogenic Th17 cells into pathogenic cells that induce autoimmunity [52]. Soluble CD5L is a biomarker of disease activity in SLE [51].

*LGALS2* encodes galectin-2, which binds with high affinity to CD14-expressing monocytes and induces production of proinflammatory cytokines [32]. CD14 is a genetic susceptibility locus for SLE, and soluble CD14 is a biomarker of disease activity [53].

*SLAMF7* is a cell surface protein expressed by immune cells and has been shown to have a varied role in the immune response, including activation of inflammatory macrophages [54], activation of NK cells, and B cell proliferation and autocrine signaling [55].

*SIGLEC1*, expressed exclusively in cells of myeloid lineage, encodes the lectin sialoadhesin (CD169). In children with SLE, expression of CD169 on monocytes is strongly associated with disease activity [56]. In UKB-PPP, soluble sialoadhesin has the strongest association with SLE of all proteins measured. Although *SIGLEC1* is an interferon-stimulated gene, circulating levels of the protein are not associated with the same *trans*-QTLs that regulate the interferon signature.

*CD300E* is a surface receptor for sphingomyelin expressed by myeloid cells. Initially considered as an immune activating receptor, it has more recently been shown to downregulate T cell activation by impairing antigen presentation [57].

#### Genes encoding cytokines

*CXCL13*, *CXCL10* and *IL5RA* are chemokines and cytokines, whose role is to regulate immune responses by acting as signaling molecules. CXCL13 is a biomarker of active SLE and is elevated in SLE compared to other autoimmune diseases [17, 58].

#### Other genes

The possible roles of *ALKBH2* and *SHISA5* on SLE pathophysiology are not obvious. *ALKBH2* encodes a DNA repair enzyme that mainly affects double-stranded DNA structures. As defective DNA degradation and clearance has a key role in the pathogenesis of SLE, it is plausible that an enzyme that processes DNA could be relevant. *SHISA5*, also known as SCOTIN, encodes an endoplasmic reticulum membrane protein that is involved in caspase-dependent cellular apoptosis. Its expression is induced by type I interferon [59].

### Concluding remarks

Using aggregated *trans*-effects of common SNPs on transcript and protein levels, we identified interferon signaling and 20 genes outside the HLA region as putative core genes for SLE. A limitation of our study is that genetic prediction of *trans*-regulated gene expression relies on eQTLGen Phase I, which tested only 10,316 preselected SNPs for *trans*-associations. eGATE scores account for only about 2% of the total variance in transcript levels of the detected ISGs and about 5% of the variance attributable to *trans*-effects (Supplementary Table S4). Because the underlying effect of upregulation of interferon signaling on SLE is large, it is easily detected in case-control datasets of a few thousand individuals despite the weak prediction of gene expression by a *trans*-eQTL score derived from eQTLGen Phase 1. More comprehensive eQTL summary statistics from eQTLGen Phase 2 will increase the power of GATE analysis to detect core genes based on predicted transcript levels.

Our genetic analyses were restricted to people of European ancestry both in terms of the *trans*-effects on gene expression and the associations of the aggregated effects with disease. Polygenic risk scores for complex diseases do not generalize well across populations with different genetic ancestries [60]. SNP frequencies can differ substantially between ancestries, and thus the effects of specific genetic variants (e.g., GWAS hits) will also differ. On the other hand, it has been suggested that the effect sizes of core genes on disease are more likely to remain consistent across populations, since these would reflect the fundamental biology of the disease [61]. To evaluate this hypothesis using GATE analysis requires large-scale genetic studies of gene expression and protein levels in diverse populations in order to learn good predictors of gene expression that generalize across ancestries, before testing the aggregated scores for association with disease in different populations.

Our results provide further support for the primary role of type I interferon signaling in SLE. A novel contribution of our paper is to show that upregulation of interferon signaling via *trans*-effects of common SNPs accounts for a substantial proportion (around 9%) of the total genetic effect on SLE risk. The ISGs that are genetically upregulated in SLE are genetically downregulated in inflammatory bowel disease (McKeigue et al*.* [20], Inflammatory Bowel Diseases, in press), and some of these effects are attributable to the same *trans*-eQTLs. While the role of interferon signaling is well-established in SLE, and anifrolumab, a biologic drug blocking interferon signaling, has been recently approved for SLE [30, 31], this validation of GATE analysis in SLE provides support for targeting deficient interferon signaling in inflammatory bowel disease.

These results also demonstrate that *trans*-effects on transcript levels are not restricted to effects on gene transcription within the same cell type but can operate through signaling between cells of different types. The type I interferons released by plasmacytoid dendritic cells in response to signaling from MDA5 and RIG-I bind to receptors on a broad range of immune cell types to activate transcription of ISGs via the JAK/STAT pathway.

Our results confirm signaling from BAFF and APRIL to the receptors BCMA and TACI on B cells as a core pathway for SLE. This pathway is already targeted by the BAFF inhibitor belimumab, which has been approved for use in SLE since 2011 [33], and for lupus nephritis more recently where it appears to be more effective. Our findings support further efforts to develop more effective drugs targeting this pathway. A Phase 2 clinical trial for a more potent dual BAFF/APRIL antagonist (povetacicept) for autoimmune kidney diseases, including lupus nephritis, is under way [62].

Several other proteins encoded by the core genes identified in this study are possible therapeutic targets. So far three have been validated in experimental models: deletion of *TNFRSF9* and *PDCD1* causes lupus, while overexpression of *FCRL5* causes lupus. We recently reported *PDCD1* as the top core gene for rheumatoid arthritis identified by GATE analysis [28], and PD-1 (encoded by *PDCD1*) agonists have undergone a successful Phase 2 trial for rheumatoid arthritis [63]. Our data support that these drugs should be trialed also for SLE. The druggability of 4-1BB and FcRL5 (encoded by *TNFRSF9* and *FCRL5*, respectively) should be studied to determine if these could act as therapeutic targets for SLE. 4-1BB agonists have reached clinical stage as immunotherapy for cancer [64]. The remaining fourteen identified core proteins require validation in an experimental model of lupus.

## Supplementary information

Consortia information

Supplementary Materials

## Data Availability

The code used in this study is available at https://github.com/molepi-precmed/trans-qtls. Summary-level data for the association of SLE with all tested GATE scores has been deposited to Zenodo and available at 10.5281/zenodo.16412020. Data from the PRECISESADS study is available upon request at ELIXIR Luxemburg, except the GWAS data that cannot be anonymized, with the permanent link: 10.17881/th9v-xt85. The access procedure is described on the ELIXIR data landing page. The platform used to compute locus-specific genotypic scores and the database of published GWAS summary statistics are accessible via a web API on the https://genoscores.cphs.mvm.ed.ac.uk/ platform. Access requests to the genoscores platform and the corresponding R package should be directed to the corresponding author. Code for running the Bayesian Mendelian randomization analysis is available at https://github.com/molepi-precmed/mrhevo. UK Biobank data may be accessed by completing an application at https://www.ukbiobank.ac.uk/.
