# Supplementary material for: Discovery of core genes for systemic lupus erythematosus via genome-wide aggregated trans-effects analysis
Source: Genes Immun. 2025 Sep 3;26(5):497–508. doi: 10.1038/s41435-025-00352-4 (PMC12527927; doi:10.1038/s41435-025-00352-4)
Supplement: Supplementary file 1 — Consortia information [file 41435_2025_352_MOESM1_ESM.docx]

**PRECISESADS Clinical Consortium**

Lorenzo Beretta^8^, Barbara Vigone^8^, Jacques‐Olivier Pers^9^, Alain Saraux^9^, Valérie Devauchelle‐Pensec^9^, Divi Cornec^9^, Sandrine Jousse‐Joulin^9^, Bernard Lauwerys^10^, Julie Ducreux^10^, Anne‐Lise Maudoux^10^, Carlos Vasconcelos^11^, Ana Tavares^11^, Esmeralda Neves^11^, Raquel Faria^11^, Mariana Brandão^11^, Ana Campar^11^, António Marinho^11^, Fátima Farinha^11^, Isabel Almeida^11^, Miguel Angel Gonzalez‐Gay Mantecón^12^, Ricardo Blanco Alonso^12^, Alfonso Corrales Martínez^12^, Ricard Cervera^13^, Ignasi Rodríguez‐Pintó^13^, Gerard Espinosa^13^, Rik Lories^14^, Ellen De Langhe^14^, Nicolas Hunzelmann^15^, Doreen Belz^15^, Torsten Witte^16^, Niklas Baerlecken^16^, Georg Stummvoll^17^, Michael Zauner^17^, Michaela Lehner^17^, Eduardo Collantes^18^, Rafaela Ortega Castro^18^, Ma Angeles Aguirre‐Zamorano^18^, Alejandro Escudero‐Contreras^18^, Mª Carmen Castro‐Villegas^19^, Norberto Ortego^19^, María Concepción Fernández Roldán^19^, Enrique Raya^20^, Inmaculada Jiménez Moleón^20^, Enrique de Ramon^21^, Isabel Díaz Quintero^21^, Pier Luigi Meroni^22^, Maria Gerosa^22^, Tommaso Schioppo^22^, Carolina Artusi^22^, Carlo Chizzolini^23^, Aleksandra Zuber^23^, Donatienne Wynar^23^, Laszló Kovács^24^, Attila Balog^24^, Magdolna Deák^24^, Márta Bocskai^24^, Sonja Dulic^24^, Gabriella Kádár^24^, Falk Hiepe^25^, Velia Gerl^25^, Silvia Thiel^25^, Manuel Rodriguez Maresca^26^, Antonio López‐Berrio^26^, Rocío Aguilar‐Quesada^26^ & Héctor Navarro‐Linares^26^

^8^Referral Center for Systemic Autoimmune Diseases, Fondazione IRCCS Ca’ Granda Ospedale Maggiore Policlinico di Milano, Milano, Italy. ^9^Centre Hospitalier Universitaire de Brest, Hospital de la Cavale Blanche, Brest, France. ^10^Pôle de pathologies rhumatismales systémiques et inflammatoires, Institut de Recherche Expérimentale et Clinique, Université catholique de Louvain, Brussels, Belgium. ^11^Centro Hospitalar do Porto, Porto, Portugal. ^12^Servicio Cantabro de Salud, Hospital Universitario Marqués de Valdecilla, Santander, Spain. ^13^Hospital Clinic I Provicia, Institut d’Investigacions Biomèdiques August Pi i Sunyer, Barcelona, Spain. ^14^Katholieke Universiteit Leuven, Leuven, Belgium. ^15^Klinikum der Universitaet zu Koeln, Cologne, Germany. ^16^Medizinische Hochschule Hannover, Hannover, Germany. ^17^Medical University Vienna, Vienna, Austria. ^18^Servicio Andaluz de Salud, Hospital Universitario Reina Sofía Córdoba, Córdoba, Spain. ^19^Servicio Andaluz de Salud, Complejo hospitalario Universitario de Granada (Hospital Universitario San Cecilio), Granada, Spain. ^20^Servicio Andaluz de Salud, Complejo hospitalario Universitario de Granada (Hospital Virgen de las Nieves), Granada, Spain. ^21^Servicio Andaluz de Salud, Hospital Regional Universitario de Málaga, Málaga, Spain. ^22^Università degli studi di Milano, Milan, Italy. ^23^Hospitaux Universitaires de Genève, Genève, Switzerland. ^24^University of Szeged, Szeged, Hungary. ^25^Charite, Berlin, Germany. ^26^Andalusian Public Health System Biobank, Granada, Spain.

**PRECISESADS Flow Cytometry Study Group**

Montserrat Alvarez^27^, Damiana Alvarez‐Errico^28^, Nancy Azevedo^29^, Nuria Barbarroja^30,31^, Anne Buttgereit^32^, Qingyu Cheng^33^, Carlo Chizzolini^27^, Jonathan Cremer^34^, Aurélie De Groof^35^, Ellen De Langhe^36^, Julie Ducreux^37^, Aleksandra Dufour^27^, Velia Gerl^33^, Maria Hernandez‐Fuentes^37^, Laleh Khodadadi^33^, Katja Kniesch^38^, Tianlu Li^33^, Chary Lopez‐Pedrera^35^, Zuzanna Makowska^32^, Concepción Marañón^2^, Brian Muchmore^2^, Esmeralda Neves^29^, Bénédicte Rouvière^39^, Quentin Simon^39^, Elena Trombetta^40^, Nieves Varela^38^ & Torsten Witte^38^

^27^Immunology and Allergy, University Hospital and School of Medicine, Geneva, Switzerland. ^28^Chromatin and Disease Group, Bellvitge Biomedical Research Institute (IDIBELL), Barcelona, Spain. ^29^Serviço de Imunologia EX-CICAP, Centro Hospitalar e Universitário do Porto, Porto, Portugal. ^30^IMIBIC, Reina Sofia Hospital, University of Cordoba, Córdoba, Spain. ^31^Bayer AG, Berlin, Germany. ^32^Pharmaceuticals Division, Bayer Pharma, Berlin, Germany. ^33^Department of Rheumatology and Clinical Immunology, Charité University Hospital, Berlin, Germany. ^34^Department of Microbiology and Immunology, Laboratory of Clinical Immunology, KU Leuven, Leuven, Belgium. ^35^Pôle de Pathologies Rhumatismales Inflammatoires et Systémiques, Institut de Recherche Expérimentale et Clinique, Université Catholique de Louvain, Brussels, Belgium. ^36^University Hospitals Leuven and Skeletal Biology and Engineering Research Center, KU Leuven, Leuven, Belgium. ^37^UCB, Slough, UK. ^38^Klinik für Immunologie Und Rheumatologie, Medical University Hannover, Hannover, Germany. ^39^NSERM, UMR1227, CHRU Morvan, Lymphocytes B et Autoimmunité, University of Brest, BP 824, Brest, France. ^40^Laboratorio di Analisi Chimico Cliniche e Microbiologia - Servizio di Citofluorimetria, Fondazione IRCCS Ca’ Granda Ospedale Maggiore Policlinico di Milano, Milan, Italy.

[www.precisesads.eu](http://www.precisesads.eu)
