# Supplementary material for: Discovery of core genes for systemic lupus erythematosus via genome-wide aggregated trans-effects analysis
Source: Genes Immun. 2025 Sep 3;26(5):497–508. doi: 10.1038/s41435-025-00352-4 (PMC12527927; doi:10.1038/s41435-025-00352-4)
Supplement: Supplementary file 2 — Supplementary Materials [file 41435_2025_352_MOESM2_ESM.pdf]

# Discovery of core genes for systemic lupus erythematosus via genome-wide aggregated *trans*-effects analysis

Andrii Iakovliev† [1] , Olivia Castellini-Pérez† [2, 3] , Buddhiprabha Erabadda [4] , PRECISESADS Clinical Consortium ‡, PRECISESADS Flow Cytometry Consortium ‡, Javier Martín [5] , Guillermo Barturen [2, 6] , Paul M McKeigue [4] , Elena Carnero-Montoro [2, 3] , Marta E Alarcón-Riquelme [2, 7] , Athina Spiliopoulou [4] *

**1** Institute of Genetics and Cancer, College of Medicine and Veterinary Medicine, University of Edinburgh, Crewe Road South, Edinburgh EH4 2XU, Scotland, United Kingdom.
**2** GENYO. Pfizer-University of Granada-Junta de Andalucía Centre for Genomics and Oncological Research, 18016, Parque Tecnológico de la Salud, Granada, Spain.
**3** University of Granada, Spain
**4** Usher Institute, College of Medicine and Veterinary Medicine, University of Edinburgh, BioQuarter - Gate 3, 5-7 Little France Road, Edinburgh EH16 4UX, Scotland, United Kingdom.
**5** IBPLN-CSIC, Instituto de Parasitología y Biomedicina "López-Neyra" (CSIC) Avda. Conocimiento 17 Parque Tecnologico Ciencias de la Salud 18016 Armilla - Granada, Spain.
**6** Department of Genetics, Faculty of Sciences, University of Granada, Granada, Spain
**7** Unit of Chronic Inflammation, Institute for Environmental Medicine, Karolinska Institutet, Stockholm, Sweden.

* Corresponding author: a.spiliopoulou@ed.ac.uk

## Supplementary Methods

### SLE GWAS datasets

We used individual-level data for SLE cases and controls of European ancestry from the following three genetic datasets:

(1) 4,946 SLE cases and 7,013 controls from the Bentham *et al* (2015) study[1] . Samples and clinical data for this study were contributed by different centres in the UK, Europe and Canada, and obtained via the BIOLUPUS network. Southern European cases were matched with 1,286 healthy controls mostly from southern Europe, while the remaining 5,727 controls were taken from the University of Michigan Health and Retirement Study (HRS). The HRS controls were genotyped using the Illumina Human2.5M Beadchip, while the SLE cases and remaining controls were genotyped with the Illumina HumanOmni1-Quad BeadChip.

(2) 730 SLE cases and 475 controls obtained by members of the International Consortium for Systemic Lupus Erythematosus Genetics (SLEGEN)[2]. All study participants self-identified as women of European ancestry. Cases and controls were genotyped with the Illumina Infinium HumanHap300 genotyping Bead-Chip.

(3) 428 SLE cases and 591 controls from the PRECISESADS cohort, which recruited people at 19 institutions in 9 European countries and across 7 systemic autoimmune diseases[3]. Cases and controls were genotyped with the IIllumina HumanCore-24 v1.0 chip.

†Contributed equally to this work.
‡Full list of consortia authors and affiliations is in the supplementary material.

Quality control, phasing and imputation of the BIOLUPUS/HRS study and the SLEGEN study have been previously described by the corresponding studies[1,2]. Imputation of the PRECISESADS study was carried out using the Michigan Imputation Server with the Haplotype Reference Consortium as the reference panel. All three datasets underwent ancestry checks based on genetic similarity as described in each of the corresponding studies, with cases and controls of European ancestry included in our analyses.

### Computation of GATE scores

GATE analysis proceeds in three analytical steps conducted for each gene or protein: (1) summary statistics for expression/protein quantitative trait loci (QTL) are extracted and processed to compute a weights vector for the SNPs in each locus, (2) a genotypic score for each individual in a case-control dataset is computed by adding up their genotypes at *trans*-QTLs across the genome, weighted by the weights vector from step 1, (3) this genotypic score, corresponding to the predicted level of the gene's *trans*-regulated expression/protein level, is tested for association with the disease. Here, we describe each of these steps in more detail.

QTL summary statistics were extracted from three large GWAS of gene expression and of protein level: (1) eQTLGen, which analyzed gene expression in whole blood in 31,684 healthy individuals of European ancestry[4], (2) deCODE, which measured 4,719 circulating proteins on the SomaLogic v4 panel based on plasma samples from 35,559 Icelanders[5], and (3) the UK Biobank Pharma Proteomics Project (UKB-PPP), which measured 2,923 circulating proteins on the Olink Explore panel based on plasma samples from 54,219 UK Biobank participants[6]. eQTLGen tested only 10,317 pre-selected trait-associated SNPs for association with gene expression in *trans*, which limits the number of *trans*-eQTLs contributing to GATE scores.

A *trans*-pQTL at the *CFH* locus was present for 1,975 proteins in deCODE. This highly pleiotropic *trans*-pQTL has been attributed to nonspecific binding of complement factor H, the protein encoded by *CFH*, to SomaLogic aptamers[7]. As this binding could interfere with the protein measurement, these 1,975 proteins were excluded from our analyses.

GATE scores were computed in the two case-control datasets (discovery and replication) using the GENOSCORES platform[8]. For each gene (or protein), GWAS summary statistics for SNPs typed or imputed in the case-control dataset were extracted. QTLs were defined as containing at least one SNP with $p < 10^{-6}$ and being separated by at least 500 kb from other SNPs associated with gene expression at $p < 10^{-5}$. For each locus, univariate regression coefficients for SNPs with $p < 10^{-5}$ were included in a locus-specific weights vector. This vector was premultiplied by the inverse correlation matrix between SNP genotypes, computed in the European ancestry subset of the 1000 Genomes reference panel (phase 3)[9] to adjust for linkage disequilibrium. This operation approximates the multivariate coefficients that would be obtained by fitting the SNPs jointly through multiple regression analysis of gene expression in the original GWAS dataset. The operation has been implemented using a pseudo-inverse solution to handle ill-conditioned matrices in loci with highly correlated SNPs. For each gene (or protein) and each QTL, a locus-specific score was computed by multiplying the vector of adjusted weights by the matrix of SNP genotypes in the case-control dataset.

Locus-specific scores were classified as *cis*, *cis-x* or *trans* if the distance from the locus to the transcription site of the respective gene was less than or equal to 50 kb, between 50 kb and 5 Mb, and more than 5 Mb, respectively. The start and end positions of the transcription site of each gene were obtained from Ensembl[10]. Mapping of proteins to the gene encoding them was obtained from Uniprot[11]. The eGATE (pGATE) score for each gene (protein) was computed as the sum of locus-specific *trans*-scores for the corresponding gene expression (or protein level).

## Genetic contribution to disease attributable to *trans*-effects on a core gene

Under a model where many risk loci act multiplicatively on disease risk (additively on the logistic scale), the C-statistic (or the area under the ROC curve, AUC) of a logistic regression model regressing the risk of disease on all relevant genetic loci can be derived from the sibling recurrence risk ratio, $\lambda_s$, as $C = 1 - \Phi\left(-\sqrt{\log(\lambda_s)}\right)$, where $\Phi(\cdot)$ is the Gaussian cumulative distribution function[12,13]. This relation can also be interpreted in terms of the expected information for discriminating between cases and non-cases, $\Lambda_{total}$, with $\Lambda_{total} = \log(\lambda_s)$[13]. For SLE, the estimated sibling recurrence risk ratio is 16, giving a total genetic information for discrimination of 2.78 nats (log(16)) or 4 bits ($\log_2(16)$). Using these formulas, information values of (1, 2, 3, 4) bits are equivalent to C-statistic values of (0.80, 0.88, 0.925, 0.952).

If the log odds ratio (the linear predictor in a logistic regression model) has a Gaussian distribution in non-cases, then the distribution of the log odds ratio in cases is also Gaussian, the two class-conditional distributions have the same variance[12], and the information for discrimination conveyed by the linear predictor is half this variance. If a GATE score for a single gene is based on multiple SNPs and multiple QTLs, its distribution is approximately Gaussian, and thus the information for discrimination is equal to half the square of the standardized log odds ratio for its association with the disease, $\Lambda = \beta^2/2$ nats. As the GATE score is an imperfect predictor of *trans*-regulated expression, to calculate the expected information for discrimination that would be obtained from an optimal predictor, we need to account for the dilution of the underlying genetic effect. Let $r^2$ denote the proportion of variance in expression explained by the GATE score, and let $h^2_{trans}$ denote the *trans*-heritability of the expression of a gene, i.e. the proportion of variance in expression attributable to *trans*-effects. For an optimal predictor of *trans*-regulated expression, $r^2$ would be equal to $h^2_{trans}$. Putting these steps together, the genetic information for discrimination (in bits) attributable to *trans*-effects on expression of a gene and corrected for dilution by the factor $r^2/h^2_{trans}$ was calculated as:

$$I = \frac{\beta^2}{2 \times \log(2)} \Big/ \frac{r^2}{h^2_{trans}} \tag{1}$$

The squared correlation between GATE score and measured expression of the gene, $r^2$, was computed in the transcriptomics and proteomics data for eGATE and pGATE scores, respectively. Estimates for *trans*-heritability ($h^2_{trans}$) for the expression of each gene were downloaded from supplementary table 2 of Ouwens *et al* (2020)[14]. Estimates for $h^2_{trans}$ for protein levels were downloaded from supplementary table 19 of Sun *et al* (2023)[6].

To compute the information for discrimination attributable to the HLA-DRB1*03:01 allele, we used the reported odds ratio of 1.87 for its effect on SLE risk[15] and the frequency of the allele estimated in a European population ($p = 0.0928$)[16] to get the standardized effect size ($\beta = \log(OR)\sqrt{2p(1-p)}$).

## Criteria for identifying putative core genes

*Consistency of association* Where some of the *trans*-QTLs associated with disease have pleiotropic effects on gene expression (i.e. affect the expression of multiple genes), associations of disease with GATE scores for a gene may be confounded by the effects of these *trans*-QTLs on expression of other genes that are directly causal. GATE scores were thus filtered to retain only those for which the number of aggregated *trans*-QTLs was greater than 5. The rationale for this is that, when a gene is causal, effects from multiple *trans*-QTLs will be consistent in direction. When GATE scores are formed by aggregating many *trans*-QTLs, the ratio of "signal" (causal effects consistent in direction) to "noise" (effects that are random in direction) is increased.

*Strength of association* Putative core genes were identified using a threshold of $p < 10^{-6}$ for the association of SLE risk with an eGATE or pGATE score for a gene.

*Specificity of association* To check if associations between the disease and GATE scores could be confounded by pleiotropic *trans*-QTLs, we additionally inspected the matrix of score-score correlations. By definition, pleiotropic *trans*-QTLs affect the expression of more than one genes, and this sharing makes GATE scores for these genes correlated. For correlated scores we can distinguish two cases: association of these scores with the disease is driven by a single shared (pleiotropic) *trans*-QTL, or by multiple shared *trans*-QTLs. The first case can only occur if the pleiotropic *trans*-QTL has a very strong effect on disease, so as to be detectable despite the “noise” introduced by the other *trans*-QTLs in the aggregated score. For SLE, such strong genetic signals have been detected in the HLA region, which was excluded from the GATE score computation. The second case occurs when multiple pleiotropic *trans*-QTLs affect the expression of the same genes. This can happen when genes are activated together within a regulatory pathway. In this case, correlated GATE scores reflect the shared pathway, though it is not possible to identify which genes in the pathway are causal from GATE analysis alone.
*Trans*-QTLs contributing to GATE scores of putative core genes were grouped into clumps to identify shared *trans*-effects. *Trans*-QTLs were assigned to the same clump if their genomic locations overlapped, or were within 200kb of each other.

### Additional supportive evidence for causality of putative core genes with disease

We defined six criteria to gather additional evidence that would support a GATE-detected gene as a *core* gene for SLE, where *core* reflects a strong coupling between the gene and the disease or a direct role of the gene in the disease:

1. Instrumental variable analysis (“Mendelian randomization”), based on marginalizing over the distribution of pleiotropic effects, supports a causal effect of the transcript or protein at $p < 0.01$. Similar to the approach of Grant and Burgess (2024)[17], a Bayesian model with a hierarchical shrinkage prior over the direct (pleiotropic) effects of the *trans*-QTLs on the disease was specified, and used to infer the posterior distribution over all model parameters (effects of *trans*-QTLs on exposure, causal effect of exposure on outcome, unobserved pleiotropic effects of *trans*-QTLs on outcome). The marginal likelihood of the causal effect parameter was then obtained by dividing the marginal posterior density by the prior on this parameter, and a classical hypothesis test (maximum likelihood estimate and p-value) was obtained by fitting a quadratic function to the log-likelihood. This tests whether the effects of the *trans*-QTLs on the disease are broadly consistent with their effects on the expression of the gene, allowing for possible direct effects of some *trans*-QTLs on the disease. We emphasize that Mendelian randomization analysis has limitations and that the results should be interpreted as only one line of evidence.

2. A reported genome-wide significant association with SLE in GWAS Catalog ($p < 5 \times 10^{-8}$) with a SNP within 200 kb of the transcription site of the putative core gene or an association of SLE with a *cis*-score for the gene at $p < 0.01$ and consistent direction of effect. As *cis*-SNPs (all SNPs within 5 Mb of the transcription site of a gene) were excluded from the GATE score, any association with SNPs near the transcription site is independent evidence in support of a causal role of the gene in the disease.

3. Association of disease with measured level of the transcript in whole blood, or with measured level of the encoded protein. Because GATE scores typically explain only a small proportion of the variance of the measured transcript or protein, the association of disease with measured transcript or protein should be far stronger than the association with the GATE score if the association with the transcript or protein is causal. We set a threshold for the effect size (standardized log odds ratio) of the association between the disease and the direct measurement to be at least twice the effect size of the association

between the disease and the GATE score, and require that the effects are in the same direction.

4. Perturbation of the gene by knockout, transduction or over-expression, or perturbation of the gene product by an inhibitor or an agonist, alters the severity of disease in an experimental model.

5. Drugs targeting the gene product, its ligand or its receptor cause the disease, or have shown efficacy against the disease in a clinical trial.

6. Rare variants in the gene, or a gene encoding the ligand or receptor of the gene product, cause a monogenic form of the disease.

Criteria 2 to 6 do not depend on *trans*-QTL effects, so they provide independent evidence for the role of a putative core gene in the disease, as each is based on an independent line of evidence. Most core genes will not have support from GWAS studies since *cis*-acting variants that perturb disease-relevant genes are subject to purifying selection[18,19].

GWAS summary statistics for the systemic lupus erythematosus ontology (MONDO_0007915) were downloaded from the NHGRI-EBI GWAS Catalog on 05/07/2024. These were filtered to keep SNPs with reported “DISEASE/TRAIT” matching “systemic lupus erythematosus” and with $p$-value of association $p < 5 \times 10^{-8}$ and with a reported genomic position (“CHR_ID” and “CHR_POS” columns). The retained SNPs were assigned to the same genomic region if located within 200kb of each other or to different regions otherwise. This procedure resulted in 187 SLE-associated regions coming from multiple GWAS studies (GCST011096, GCST011097, GCST90011866)[20], (GCST90100586, GCST90100585)[21], (GCST005752, GCST007400)[22], (GCST003155, GCST003156)[1], GCST003622[23], GCST004867[24], GCST011956[25], GCST90020042[26], GCST000507[27], GCST001795[28], GCST003599[29], GCST003252[30], GCST90103801[31], GCST90018917[32], GCST002463[33], GCST005831[34], GCST000216[35], GCST001708[36], GCST000996[37], GCST000144[38], GCST011426[39], GCST000142[2], GCST005754[40], GCST001384[41], GCST000143[42], (GCST011493, GCST011491, GCST90014238)[43], GCST003103[44], GCST000592[45], GCST000858[46].

Genes with known rare variants causing monogenic forms of lupus and lupus-like disease were extracted from two reviews on the topic, reporting 34 genes[47] and 38 genes[48], resulting in a total of 59 unique genes.

### PRECISESADS RNA-sequencing data

The PRECISESADS study collected various biological samples from participants, including RNA from whole blood[3]. Using the HiSeq SR Cluster Kit v4 and the cBot system (Illumina), samples were clustered and then sequenced on a HiSeq2500 device over three separate runs with the HiSeq SBS Kit v4 (Illumina). Raw sequencing data underwent preprocessing with bcl2fastq software, and quality control was performed using FastQC tools. Sequencing reads were aligned to the UCSC Homo sapiens reference genome (Build hg19) utilizing STAR v2.5.2b[49]. Gene-level expression quantification was conducted with RSEM v1.2.31[50], producing estimates in Transcripts Per Million (TPM) and read counts. For RNA quality control, samples were required to meet the following criteria: i) over 7 million reads mapped to genes, and ii) an RNA Integrity Number greater than 7. Additionally, a pre-filtering step was performed to exclude genes with fewer than 5 reads in at least 500 samples.

## Supplementary References


1. Bentham J *et al.* Genetic association analyses implicate aberrant regulation of innate and adaptive immunity genes in the pathogenesis of systemic lupus erythematosus. *Nature Genetics* **47**, 1457–1464 (2015) doi:10.1038/ng.3434.
2. International Consortium for Systemic Lupus Erythematosus Genetics (SLEGEN) *et al.* Genome-wide association scan in women with systemic lupus erythematosus identifies susceptibility variants in ITGAM, PXK, KIAA1542 and other loci. *Nature Genetics* **40**, 204–210 (2008) doi:10.1038/ng.81.
3. Barturen G *et al.* Integrative Analysis Reveals a Molecular Stratification of Systemic Autoimmune Diseases. *Arthritis & Rheumatology* **73**, 1073–1085 (2021) doi:10.1002/art.41610.
4. Võsa U *et al.* Large-scale cis- and trans-eQTL analyses identify thousands of genetic loci and polygenic scores that regulate blood gene expression. *Nature Genetics* **53**, 1300–1310 (2021) doi:10.1038/s41588-021-00913-z.
5. Ferkingstad E *et al.* Large-scale integration of the plasma proteome with genetics and disease. *Nature Genetics* **53**, 1712–1721 (2021) doi:10.1038/s41588-021-00978-w.
6. Sun BB *et al.* Plasma proteomic associations with genetics and health in the UK Biobank. *Nature* **622**, 329–338 (2023) doi:10.1038/s41586-023-06592-6.
7. Pietzner M *et al.* Genetic architecture of host proteins involved in SARS-CoV-2 infection. *Nature Communications* **11**, 6397 (2020) doi:10.1038/s41467-020-19996-z.
8. Spiliopoulou A *et al.* Association of response to TNF inhibitors in rheumatoid arthritis with quantitative trait loci for CD40 and CD39. *Annals of the Rheumatic Diseases* **78**, 1055–1061 (2019) doi:10.1136/annrheumdis-2018-214877.
9. The 1000 Genomes Project Consortium. A global reference for human genetic variation. *Nature* **526**, 68–74 (2015).
10. Martin FJ *et al.* Ensembl 2023. *Nucleic Acids Research* **51**, D933–D941 (2023) doi:10.1093/nar/gkac958.
11. The UniProt Consortium. UniProt: The Universal Protein Knowledgebase in 2023. *Nucleic Acids Research* **51**, D523–D531 (2023) doi:10.1093/nar/gkac1052.
12. Clayton DG. Prediction and Interaction in Complex Disease Genetics: Experience in Type 1 Diabetes. *PLOS Genetics* **5**, e1000540 (2009) doi:10.1371/journal.pgen.1000540.
13. McKeigue P. Quantifying performance of a diagnostic test as the expected information for discrimination: Relation to the C-statistic. *Statistical Methods in Medical Research* **28**, 1841–1851 (2019) doi:10.1177/0962280218776989.
14. Ouwens KG *et al.* A characterization of cis- and trans-heritability of RNA-Seq-based gene expression. *European Journal of Human Genetics* **28**, 253–263 (2020) doi:10.1038/s41431-019-0511-5.
15. Morris DL *et al.* Unraveling Multiple MHC Gene Associations with Systemic Lupus Erythematosus: Model Choice Indicates a Role for HLA Alleles and Non-HLA Genes in Europeans. *The American Journal of Human Genetics* **91**, 778–793 (2012) doi:10.1016/j.ajhg.2012.08.026.
16. Rendine S *et al.* Estimation of human leukocyte antigen class I and class II high-resolution allele and haplotype frequencies in the Italian population and comparison with other European populations. *Human Immunology* **73**, 399–404 (2012) doi:10.1016/j.humimm.2012.01.005.

17. Grant AJ, Burgess S. A Bayesian approach to Mendelian randomization using summary statistics in the univariable and multivariable settings with correlated pleiotropy. *The American Journal of Human Genetics* **111**, 165–180 (2024) doi:10.1016/j.ajhg.2023.12.002.

18. Wang X, Goldstein DB. Enhancer Domains Predict Gene Pathogenicity and Inform Gene Discovery in Complex Disease. *The American Journal of Human Genetics* **106**, 215–233 (2020) doi:10.1016/j.ajhg.2020.01.012.

19. Mostafavi H, Spence JP, Naqvi S, Pritchard JK. Systematic differences in discovery of genetic effects on gene expression and complex traits. *Nature Genetics* **55**, 1866–1875 (2023) doi:10.1038/s41588-023-01529-1.

20. Wang Y-F *et al.* Identification of 38 novel loci for systemic lupus erythematosus and genetic heterogeneity between ancestral groups. *Nature Communications* **12**, 772 (2021) doi:10.1038/s41467-021-21049-y.

21. Wang Y-F *et al.* Identification of Shared and Asian-Specific Loci for Systemic Lupus Erythematosus and Evidence for Roles of Type III Interferon Signaling and Lysosomal Function in the Disease: A Multi-Ancestral Genome-Wide Association Study. *Arthritis & Rheumatology (Hoboken, N.J.)* **74**, 840–848 (2022) doi:10.1002/art.42021.

22. Langefeld CD *et al.* Transancestral mapping and genetic load in systemic lupus erythematosus. *Nature Communications* **8**, 16021 (2017) doi:10.1038/ncomms16021.

23. Morris DL *et al.* Genome-wide association meta-analysis in Chinese and European individuals identifies ten new loci associated with systemic lupus erythematosus. *Nature Genetics* **48**, 940–946 (2016) doi:10.1038/ng.3603.

24. Gateva V *et al.* A large-scale replication study identifies TNIP1, PRDM1, JAZF1, UHRF1BP1 and IL10 as risk loci for systemic lupus erythematosus. *Nature Genetics* **41**, 1228–1233 (2009) doi:10.1038/ng.468.

25. Yin X *et al.* Meta-analysis of 208370 East Asians identifies 113 susceptibility loci for systemic lupus erythematosus. *Annals of the Rheumatic Diseases* **80**, 632–640 (2021) doi:10.1136/annrheumdis-2020-219209.

26. Zhang Y-M *et al.* Shared genetic study gives insights into the shared and distinct pathogenic immunity components of IgA nephropathy and SLE. *Molecular genetics and genomics: MGG* **296**, 1017–1026 (2021) doi:10.1007/s00438-021-01798-7.

27. Han J-W *et al.* Genome-wide association study in a Chinese Han population identifies nine new susceptibility loci for systemic lupus erythematosus. *Nature Genetics* **41**, 1234–1237 (2009) doi:10.1038/ng.472.

28. Yang W *et al.* Meta-analysis followed by replication identifies loci in or near CDKN1B, TET3, CD80, DRAM1, and ARID5B as associated with systemic lupus erythematosus in Asians. *American Journal of Human Genetics* **92**, 41–51 (2013) doi:10.1016/j.ajhg.2012.11.018.

29. Lessard CJ *et al.* Identification of a Systemic Lupus Erythematosus Risk Locus Spanning ATG16L2, FCHSD2, and P2RY2 in Koreans. *Arthritis & Rheumatology (Hoboken, N.J.)* **68**, 1197–1209 (2016) doi:10.1002/art.39548.

30. Alarcón-Riquelme ME *et al.* Genome-Wide Association Study in an Amerindian Ancestry Population Reveals Novel Systemic Lupus Erythematosus Risk Loci and the Role of European Admixture. *Arthritis & Rheumatology (Hoboken, N.J.)* **68**, 932–943 (2016) doi:10.1002/art.39504.

31. Leffers HCB *et al.* Established risk loci for systemic lupus erythematosus at NCF2, STAT4, TNPO3, IRF5 and ITGAM associate with distinct clinical manifestations: A Danish genome-wide association study. *Joint Bone Spine* **89**, 105357 (2022) doi:10.1016/j.jbspin.2022.105357.

32. Sakaue S *et al.* A cross-population atlas of genetic associations for 220 human phenotypes. *Nature Genetics* **53**, 1415–1424 (2021) doi:10.1038/s41588-021-00931-x.

33. Armstrong DL *et al.* GWAS identifies novel SLE susceptibility genes and explains the association of the HLA region. *Genes and Immunity* **15**, 347–354 (2014) doi:10.1038/gene.2014.23.

34. Julià A *et al.* Genome-wide association study meta-analysis identifies five new loci for systemic lupus erythematosus. *Arthritis Research & Therapy* **20**, 100 (2018) doi:10.1186/s13075-018-1604-1.

35. Graham RR *et al.* Genetic variants near TNFAIP3 on 6q23 are associated with systemic lupus erythematosus. *Nature Genetics* **40**, 1059–1061 (2008) doi:10.1038/ng.200.

36. Lee YH, Bae S-C, Choi SJ, Ji JD, Song GG. Genome-wide pathway analysis of genome-wide association studies on systemic lupus erythematosus and rheumatoid arthritis. *Molecular Biology Reports* **39**, 10627–10635 (2012) doi:10.1007/s11033-012-1952-x.

37. Chung SA *et al.* Differential genetic associations for systemic lupus erythematosus based on anti-dsDNA autoantibody production. *PLoS genetics* **7**, e1001323 (2011) doi:10.1371/journal.pgen.1001323.

38. Hom G *et al.* Association of systemic lupus erythematosus with C8orf13-BLK and ITGAM-ITGAX. *The New England Journal of Medicine* **358**, 900–909 (2008) doi:10.1056/NEJMoa0707865.

39. Tangtanatakul P *et al.* Meta-analysis of genome-wide association study identifies FBN2 as a novel locus associated with systemic lupus erythematosus in Thai population. *Arthritis Research & Therapy* **22**, 185 (2020) doi:10.1186/s13075-020-02276-y.

40. Liu L *et al.* Genome-wide association study identifies three novel susceptibility loci for systemic lupus erythematosus in Han Chinese. *The British Journal of Dermatology* **179**, 506–508 (2018) doi:10.1111/bjd.16500.

41. Okada Y *et al.* A genome-wide association study identified AFF1 as a susceptibility locus for systemic lupus eyrthematosus in Japanese. *PLoS genetics* **8**, e1002455 (2012) doi:10.1371/journal.pgen.1002455.

42. Kozyrev SV *et al.* Functional variants in the B-cell gene BANK1 are associated with systemic lupus erythematosus. *Nature Genetics* **40**, 211–216 (2008) doi:10.1038/ng.79.

43. Song Q *et al.* Genome-wide association study on Northern Chinese identifies KLF2, DOT1L and STAB2 associated with systemic lupus erythematosus. *Rheumatology (Oxford, England)* **60**, 4407–4417 (2021) doi:10.1093/rheumatology/keab016.

44. Demirci FY *et al.* Identification of a New Susceptibility Locus for Systemic Lupus Erythematosus on Chromosome 12 in Individuals of European Ancestry. *Arthritis & Rheumatology (Hoboken, N.J.)* **68**, 174–183 (2016) doi:10.1002/art.39403.

45. Yang W *et al.* Genome-wide association study in Asian populations identifies variants in ETS1 and WDFY4 associated with systemic lupus erythematosus. *PLoS genetics* **6**, e1000841 (2010) doi:10.1371/journal.pgen.1000841.

46. Yang J *et al.* ELF1 is associated with systemic lupus erythematosus in Asian populations. *Human Molecular Genetics* **20**, 601–607 (2011) doi:10.1093/hmg/ddq474.

47. Alperin JM, Ortiz-Fernández L, Sawalha AH. Monogenic Lupus: A Developing Paradigm of Disease. *Frontiers in Immunology* **9**, (2018) doi:10.3389/fimmu.2018.02496.

48. Qin Y, Ma J, Vinuesa CG. Monogenic lupus: Insights into disease pathogenesis and therapeutic opportunities. *Current Opinion in Rheumatology* **36**, 191 (2024) doi:10.1097/BOR.0000000000001008.

49. Dobin A *et al.* STAR: Ultrafast universal RNA-seq aligner. *Bioinformatics* **29**, 15–21 (2013) doi:10.1093/bioinformatics/bts635.

50. Li B, Dewey CN. RSEM: Accurate transcript quantification from RNA-Seq data with or without a reference genome. *BMC Bioinformatics* **12**, 323 (2011) doi:10.1186/1471-2105-12-323.

51. Tao S *et al.* Knockdown of KLF5 ameliorates renal fibrosis in MRL/lpr mice via inhibition of MX1 transcription. *Immunity, Inflammation and Disease* **11**, (2023) doi:10.1002/iid3.937.

52. Furie RA *et al.* Type I interferon inhibitor anifrolumab in active systemic lupus erythematosus (TULIP-1): A randomised, controlled, phase 3 trial. *The Lancet Rheumatology* **1**, e208–e219 (2019) doi:10.1016/S2665-9913(19)30076-1.

53. Morand EF *et al.* Trial of Anifrolumab in Active Systemic Lupus Erythematosus. *New England Journal of Medicine* **382**, 211–221 (2020) doi:10.1056/NEJMoa1912196.

54. Vinay DS, Choi JH, Kim JD, Choi BK, Kwon BS. Role of endogenous 4-1BB in the development of systemic lupus erythematosus. *Immunology* **122**, 394–400 (2007) doi:10.1111/j.1365-2567.2007.02653.x.

55. Ono C *et al.* Upregulated Fcrl5 disrupts B cell anergy and causes autoimmune disease. *Frontiers in Immunology* **14**, 1276014 (2023) doi:10.3389/fimmu.2023.1276014.

56. Nishimura H, Nose M, Hiai H, Minato N, Honjo T. Development of Lupus-like Autoimmune Diseases by Disruption of the *PD-1* Gene Encoding an ITIM Motif-Carrying Immunoreceptor. *Immunity* **11**, 141–151 (1999) doi:10.1016/S1074-7613(00)80089-8.

57. Jacobs HM *et al.* TACI haploinsufficiency protects against BAFF-driven humoral autoimmunity in mice. *European Journal of Immunology* **51**, 2225–2236 (2021) doi:10.1002/eji.202149244.

58. Singh JA, Shah NP, Mudano AS. Belimumab for systemic lupus erythematosus. *The Cochrane Database of Systematic Reviews* **2021**, CD010668 (2021) doi:10.1002/14651858.CD010668.pub2.

59. Tran NL, Schneider P, Santiago-Raber M-L. TACI-dependent APRIL signaling maintains autoreactive B cells in a mouse model of systemic lupus erythematosus. *European Journal of Immunology* **47**, 713–723 (2017) doi:10.1002/eji.201646630.

## Supplementary Figures

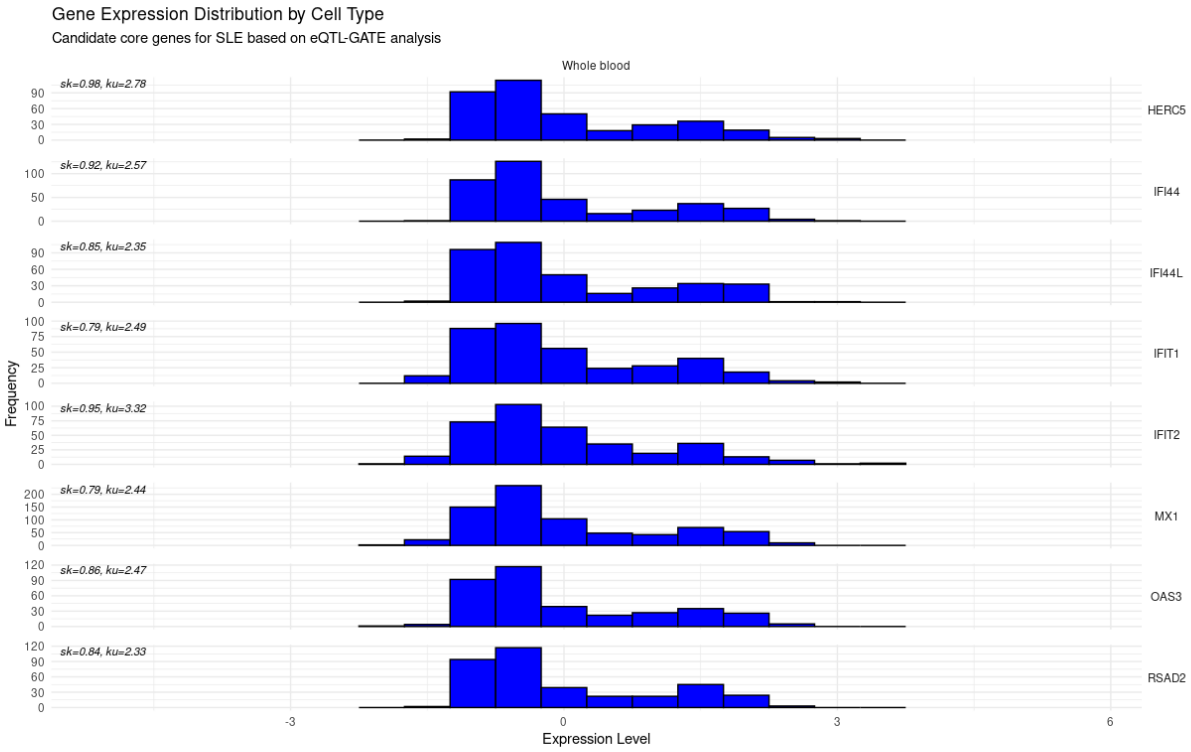


**Fig S1.** Distributions of whole-blood gene expression levels of eGATE-detected interferon stimulated genes. The skewness (sk) and kurtosis (ku) of each distribution are shown next to the respective histogram.

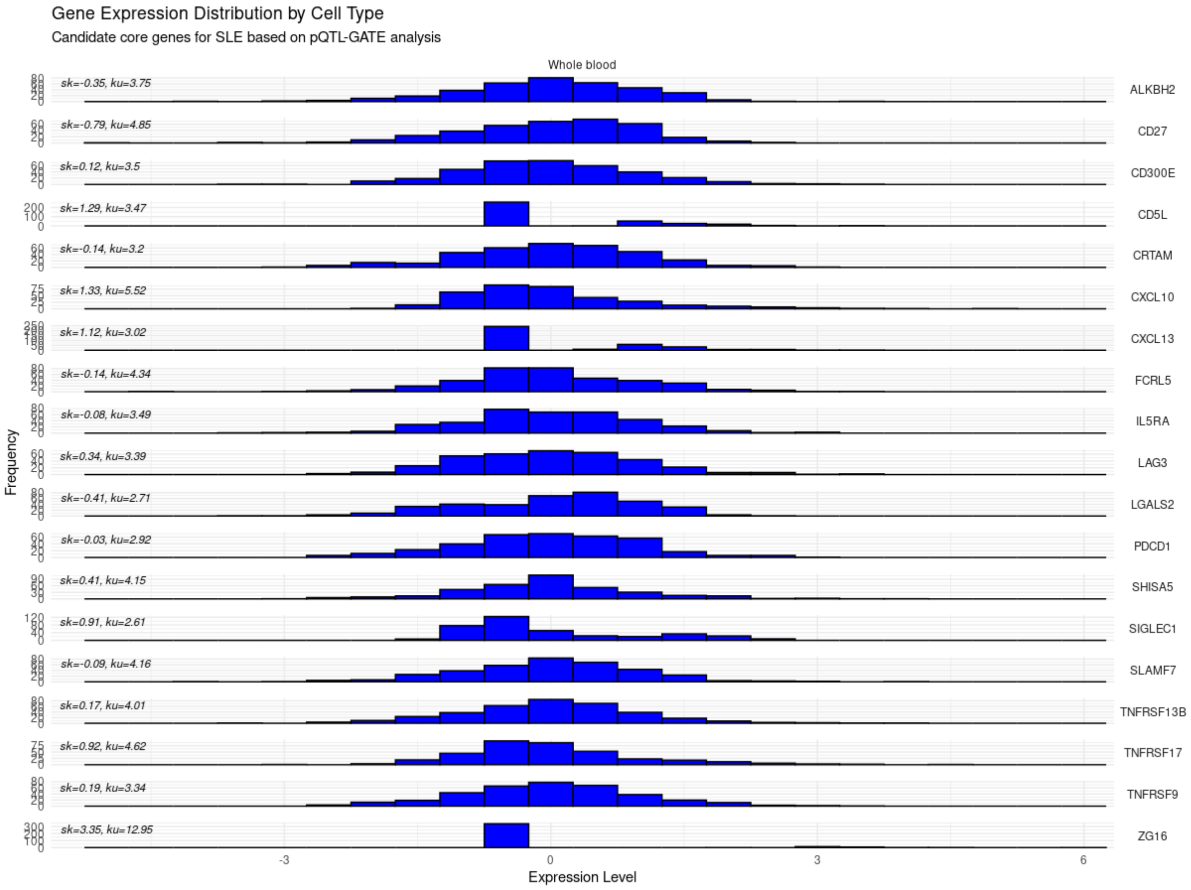


**Fig S2.** Distributions of whole-blood gene expression levels of pGATE-detected putative core genes. The skewness (sk) and kurtosis (ku) of each distribution are shown next to the respective histogram.

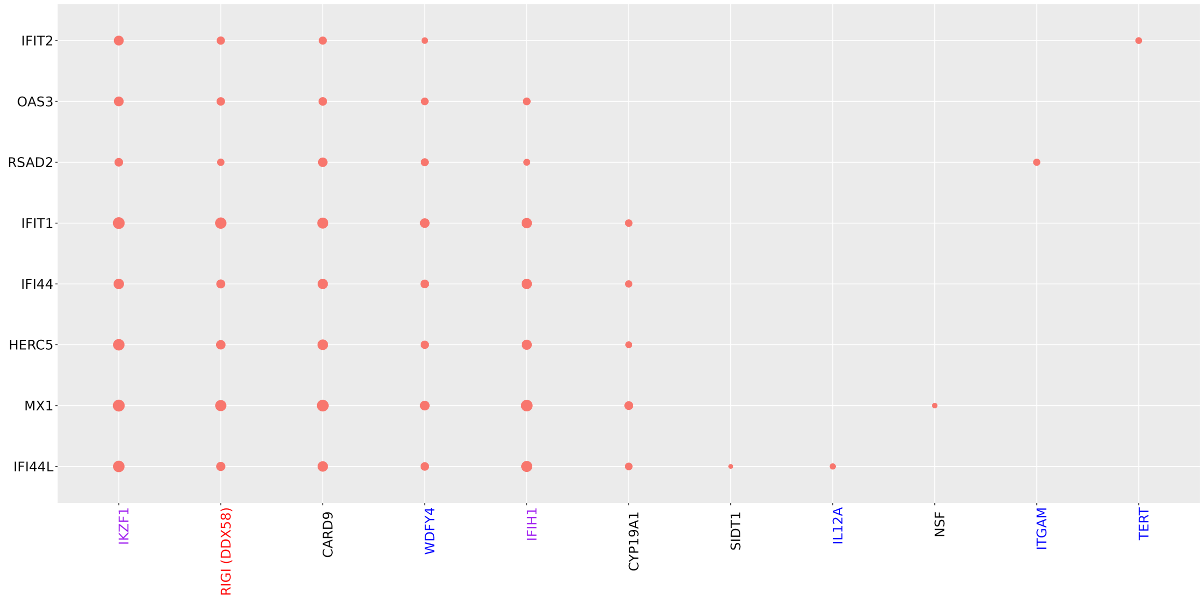


**Fig S3.** Contribution of *trans*-eQTLs clumped by genomic location ($x$-axis) on eGATE-detected ISGs ($y$-axis).
A point is drawn if a clump harbors a *trans*-eQTL for a gene. A red (teal) point reflects a positive (negative) effect of the *trans*-eQTL for that gene (based on SNPs in that clump) on SLE risk. The size of each point is inversely proportional to the standard error of the Wald ratio estimate for the effect of that gene on SLE using the corresponding *trans*-eQTL as instrumental variable. This standard error reflects our uncertainty about the estimate of the effect of the gene on SLE based on the specific *trans*-eQTL (a strong instrument and low uncertainty in the estimate of its effect on SLE would lead to a smaller standard error). Each clump is labeled by a gene within 200kb as follows: (blue text) if that gene is a GWAS hit, (red text) if that gene has rare variants known to cause monogenic lupus, or (purple text) if both conditions are true. Otherwise, the clump is labeled by the nearest gene within 200kb (black text). Information on the location of each clump and a more comprehensive list of nearby genes is given in Table S8.

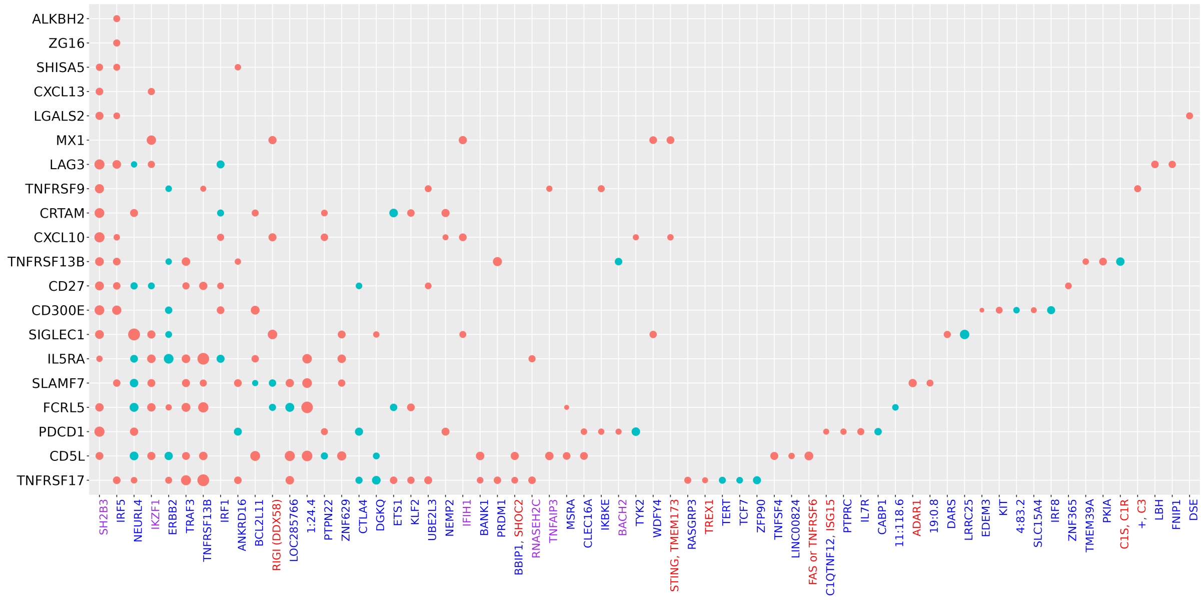


**Fig S4.** Contribution of *trans*-pQTLs near a GWAS hit or a gene for monogenic lupus clumped by genomic location ($x$-axis) on pGATE-detected putative core genes ($y$-axis).
A point is drawn if the clump harbors a *trans*-pQTL for a gene. A red (teal) point reflects a positive (negative) effect of the *trans*-pQTL for that gene (based on SNPs in that clump) on SLE risk. The size of each point is inversely proportional to the standard error of the Wald ratio estimate for the effect of that gene on SLE using the corresponding *trans*-pQTL as instrumental variable. This standard error reflects our uncertainty about the estimate of the effect of the gene on SLE based on the specific *trans*-pQTL (a strong instrument and low uncertainty in the estimate of its effect on SLE would lead to a smaller standard error).
Each clump is labeled by a gene within 200kb as follows: (blue text) if that gene is a GWAS hit (or chromosome:position of GWAS hit if it has not been attributed to a gene), (red text) if that gene has rare variants known to cause monogenic lupus, or (purple text) if both conditions are true. 64 clumps that satisfy at least one of these conditions are shown in the plot. Information on the location of all 303 clumps contributing to these putative core genes and a more comprehensive list of nearby genes for each clump is given in Table S9.
Of 187 regions with SLE GWAS hits reported in the GWAS catalog, 58 harbor *trans*-pQTLs for GATE-detected putative core genes.

## Supplementary Tables

**Table S1.** Associations of SLE with GATE scores for candidate core genes in the replication dataset (UKB).

| | UKB replication cohort | | Discovery cohort | |
|---|---|---|---|---|
| Gene symbol | Log OR | P | Log OR | P |
| ***Trans*-effects on transcript levels** | | | | |
| *(RSAD2)* | 0.161 | $1 \times 10^{-6}$ | 0.231 | $3 \times 10^{-22}$ |
| *(IFI44L)* | 0.125 | $2 \times 10^{-4}$ | 0.165 | $4 \times 10^{-12}$ |
| *(HERC5)* | 0.127 | $1 \times 10^{-4}$ | 0.156 | $6 \times 10^{-11}$ |
| *(IFI44)* | 0.131 | $7 \times 10^{-5}$ | 0.156 | $6 \times 10^{-11}$ |
| *(MX1)* | 0.141 | $2 \times 10^{-5}$ | 0.155 | $7 \times 10^{-11}$ |
| *(OAS3)* | 0.126 | $1 \times 10^{-4}$ | 0.153 | $1 \times 10^{-10}$ |
| *(IFIT1)* | 0.123 | $2 \times 10^{-4}$ | 0.152 | $2 \times 10^{-10}$ |
| *(IFIT2)* | 0.093 | 0.005 | 0.15 | $3 \times 10^{-10}$ |
| ***Trans*-effects on protein levels** | | | | |
| *CD27* | 0.057 | 0.2 | 0.177 | $1 \times 10^{-13}$ |
| *TNFRSF17* | 0.061 | 0.2 | 0.177 | $1 \times 10^{-13}$ |
| *CD5L* | 0.051 | 0.3 | 0.176 | $2 \times 10^{-13}$ |
| *LGALS2* | . | . | 0.156 | $1 \times 10^{-10}$ |
| *CXCL10* | 0.074 | 0.1 | 0.148 | $7 \times 10^{-10}$ |
| *CRTAM* | 0.052 | 0.1 | 0.145 | $2 \times 10^{-9}$ |
| *CXCL13* | . | . | 0.145 | $3 \times 10^{-9}$ |
| *TNFRSF13B* | 0.067 | 0.2 | 0.141 | $5 \times 10^{-9}$ |
| *TNFRSF9* | 0.077 | 0.1 | 0.138 | $1 \times 10^{-8}$ |
| *MX1* | . | . | 0.136 | $1 \times 10^{-8}$ |
| *SLAMF7* | 0.067 | 0.04 | 0.133 | $2 \times 10^{-8}$ |
| *SIGLEC1* | 0.088 | 0.07 | 0.134 | $4 \times 10^{-8}$ |
| *ZG16* | 0.061 | 0.7 | 0.13 | $6 \times 10^{-8}$ |
| *ALKBH2* | . | . | 0.129 | $6 \times 10^{-8}$ |
| *IL5RA* | -0.005 | 0.9 | 0.122 | $2 \times 10^{-7}$ |
| *FCRL5* | 0.058 | 0.2 | 0.123 | $3 \times 10^{-7}$ |
| *PDCD1* | 0.118 | 0.01 | 0.121 | $4 \times 10^{-7}$ |
| *LAG3* | 0.043 | 0.2 | 0.121 | $5 \times 10^{-7}$ |
| *SHISA5* | 0.067 | 0.2 | 0.119 | $6 \times 10^{-7}$ |
| *CD300E* | 0.098 | 0.04 | 0.119 | $7 \times 10^{-7}$ |

Top panel: UKB-GATE analysis based on 907 SLE cases and 450,547 controls.
Bottom panel: UKB-GATE analysis based on approximately 350 SLE cases and 400,000 controls following exclusion of samples in the UKB-PPP subset, which had been used to detect *trans*-pQTLs.
(Discovery cohort): associations of GATE scores with SLE from the main analysis presented in Table 1 for easy comparison.
Gene symbols in brackets represent a cluster of interferon stimulated genes with highly correlated GATE scores.
Log OR: log(odds ratio) for one standard deviation change in the covariate.
P: p-value of association.

**Table S2.** Causal effect estimates for the expression/protein level of GATE-detected genes (exposures) on SLE risk (outcome) using *trans*-QTLs as instrumental variables in Mendelian randomization analysis.

| Gene symbol | Study[a] | Estimate[b] | SE[b] | P[b] | N instruments[c] |
|---|---|---|---|---|---|
| **Analysis using eQTLs** | | | | | |
| *(RSAD2)* | eQTLGen | 2.11 | 0.71 | 0.003 | 6 |
| *(IFI44L)* | eQTLGen | 1.15 | 0.41 | 0.005 | 12 |
| *(HERC5)* | eQTLGen | 1.25 | 0.44 | 0.005 | 8 |
| *(IFI44)* | eQTLGen | 1.25 | 0.48 | 0.009 | 9 |
| *(MX1)* | eQTLGen | 0.85 | 0.22 | $1 \times 10^{-4}$ | 10 |
| *(OAS3)* | eQTLGen | 1.49 | 0.53 | 0.005 | 5 |
| *(IFIT1)* | eQTLGen | 0.92 | 0.26 | $4 \times 10^{-4}$ | 8 |
| *(IFIT2)* | eQTLGen | 1.84 | 0.64 | 0.004 | 6 |
| **Analysis using pQTLs** | | | | | |
| *CD27* | UKB | 0.49 | 0.23 | 0.03 | 36 |
| *TNFRSF17* | UKB | 0.63 | 0.20 | 0.002 | 46 |
| *CD5L* | UKB | 0.55 | 0.13 | $3 \times 10^{-5}$ | 64 |
| *LGALS2* | deCODE | 1.44 | 0.69 | 0.04 | 10 |
| *CXCL10* | UKB | 1.02 | 0.35 | 0.003 | 18 |
| *CRTAM* | deCODE | 0.85 | 0.40 | 0.03 | 13 |
| *CXCL13* | deCODE | 1.55 | 0.48 | 0.001 | 9 |
| *TNFRSF13B* | UKB | 0.40 | 0.19 | 0.04 | 30 |
| *TNFRSF9* | UKB | 0.92 | 0.33 | 0.006 | 21 |
| *MX1* | deCODE | 0.99 | 0.38 | 0.009 | 10 |
| *SLAMF7* | deCODE | 0.54 | 0.22 | 0.02 | 29 |
| *SIGLEC1* | UKB | 0.45 | 0.21 | 0.03 | 29 |
| *ZG16* | deCODE | 1.77 | 1.56 | 0.3 | 5 |
| *ALKBH2* | deCODE | 1.56 | 1.10 | 0.2 | 6 |
| *IL5RA* | deCODE | 0.47 | 0.16 | 0.003 | 31 |
| *FCRL5* | UKB | 0.48 | 0.18 | 0.007 | 42 |
| *PDCD1* | UKB | 0.48 | 0.22 | 0.03 | 38 |
| *LAG3* | deCODE | 0.69 | 0.26 | 0.008 | 20 |
| *SHISA5* | UKB | 0.86 | 0.78 | 0.3 | 11 |
| *CD300E* | UKB | 0.50 | 0.24 | 0.04 | 28 |

Gene symbols in brackets represent a cluster of correlated ISGs.

[a] Indicates the study from which eQTL/pQTL summary statistics were extracted. UKB denotes the UK Biobank Pharma Proteomics Project.

[b] Maximum likelihood estimate, standard error (SE), and P-value (P) for the causal effect of exposure on outcome. Estimation was performed by marginalizing over the posterior distribution of pleiotropic effects of the *trans*-QTLs.

[c] Number of independent *trans*-QTLs used as instruments in the analysis.

**Table S3.** Association of HLA-specific *trans*-scores and SLE

| Gene symbol | Chr | Start position (Mb) | Log OR | P |
|---|---|---|---|---|
| *TRBV4-1* | 7 | 142.31 | -0.22 | $1 \times 10^{-15}$ |
| *NELL2* | 12 | 44.51 | -0.20 | $5 \times 10^{-13}$ |
| *B9D2* | 19 | 41.35 | 0.20 | $7 \times 10^{-13}$ |
| *REG4* | 1 | 119.79 | -0.18 | $4 \times 10^{-10}$ |
| *TRAV26-2* | 14 | 22.20 | 0.15 | $1 \times 10^{-9}$ |
| *TNS1* | 2 | 217.80 | 0.15 | $1 \times 10^{-8}$ |
| *SSRP1* | 11 | 57.33 | 0.14 | $3 \times 10^{-8}$ |
| *LINC02446* | 12 | 10.55 | -0.19 | $6 \times 10^{-8}$ |
| *TRAV5* | 14 | 21.75 | -0.19 | $7 \times 10^{-8}$ |
| *TRBV6-6* | 7 | 142.47 | 0.16 | $1 \times 10^{-7}$ |
| *FHIT* | 3 | 59.75 | 0.16 | $1 \times 10^{-7}$ |
| *NCALD* | 8 | 101.69 | -0.22 | $1 \times 10^{-7}$ |
| *KMO* | 1 | 241.53 | 0.17 | $1 \times 10^{-7}$ |
| *EOMES* | 3 | 27.72 | -0.19 | $2 \times 10^{-7}$ |
| *RPL34* | 4 | 108.62 | -0.13 | $3 \times 10^{-7}$ |
| *TRBV5-6* | 7 | 142.50 | 0.12 | $3 \times 10^{-7}$ |
| *GZMA* | 5 | 55.10 | -0.14 | $3 \times 10^{-7}$ |
| *ZNF672* | 1 | 248.84 | -0.12 | $6 \times 10^{-7}$ |
| *HBD* | 11 | 5.23 | 0.13 | $6 \times 10^{-7}$ |
| *SPRY2* | 13 | 80.34 | -0.23 | $7 \times 10^{-7}$ |
| *TRAV41* | 14 | 22.32 | -0.12 | $8 \times 10^{-7}$ |
| *ZBTB42* | 14 | 104.80 | -0.21 | $1 \times 10^{-6}$ |
| *CLCA1* | 1 | 86.47 | -0.33 | $1 \times 10^{-38}$ |
| *TEAD4* | 12 | 2.96 | -0.33 | $1 \times 10^{-38}$ |
| *IFNA14* | 9 | 21.24 | -0.33 | $1 \times 10^{-37}$ |
| *FCRL6* | 1 | 159.80 | -0.31 | $4 \times 10^{-37}$ |
| *CELA3B* | 1 | 21.98 | -0.31 | $4 \times 10^{-36}$ |
| *C22orf15* | 22 | 23.76 | 0.32 | $4 \times 10^{-35}$ |
| *UCK2* | 1 | 165.83 | -0.31 | $1 \times 10^{-34}$ |
| *SLC6A9* | 1 | 43.99 | -0.31 | $2 \times 10^{-34}$ |
| *PRSS22* | 16 | 2.85 | -0.30 | $8 \times 10^{-33}$ |
| *NPM1* | 5 | 171.39 | -0.29 | $2 \times 10^{-32}$ |
| *GNAS* | 20 | 58.84 | -0.29 | $3 \times 10^{-32}$ |
| *FCRL1* | 1 | 157.79 | -0.31 | $3 \times 10^{-32}$ |
| *VPS28* | 8 | 144.42 | -0.29 | $2 \times 10^{-31}$ |
| *AK4* | 1 | 65.15 | -0.30 | $4 \times 10^{-31}$ |
| *TRAPPC3* | 1 | 36.14 | -0.29 | $7 \times 10^{-31}$ |
| *SIGLEC9* | 19 | 51.12 | -0.29 | $1 \times 10^{-30}$ |
| *MMP17* | 12 | 131.83 | 0.30 | $2 \times 10^{-30}$ |
| *RIC3* | 11 | 8.11 | -0.28 | $3 \times 10^{-30}$ |
| *CHST3* | 10 | 71.96 | -0.29 | $3 \times 10^{-30}$ |
| *THSD7A* | 7 | 11.37 | -0.28 | $5 \times 10^{-30}$ |
| *APCDD1* | 18 | 10.45 | -0.29 | $9 \times 10^{-30}$ |
| *TREML1* | 6 | 41.15 | -0.28 | $1 \times 10^{-29}$ |
| *RSPO2* | 8 | 107.90 | -0.29 | $1 \times 10^{-29}$ |
| *BOLA2* | 16 | 29.45 | -0.27 | $1 \times 10^{-29}$ |
| *NFATC4* | 14 | 24.37 | -0.29 | $3 \times 10^{-29}$ |
| *TSSK1B* | 5 | 113.43 | -0.27 | $3 \times 10^{-28}$ |
| *ITPKC* | 19 | 40.72 | -0.27 | $6 \times 10^{-28}$ |
| *ENTPD5* | 14 | 73.96 | -0.27 | $7 \times 10^{-28}$ |
| *POLI* | 18 | 54.27 | -0.27 | $8 \times 10^{-28}$ |
| *PPARGC1A* | 4 | 23.76 | -0.27 | $8 \times 10^{-28}$ |
| *UST* | 6 | 148.75 | -0.27 | $1 \times 10^{-27}$ |
| *RAB27B* | 18 | 54.72 | -0.26 | $2 \times 10^{-27}$ |
| *TOPBP1* | 3 | 133.60 | -0.28 | $2 \times 10^{-27}$ |

**Table S3.** Association of HLA-specific *trans*-scores and SLE *(continued)*

| Gene symbol | Chr | Start position (Mb) | Log OR | P |
|---|---|---|---|---|
| *TXNDC15* | 5 | 134.87 | -0.27 | $3 \times 10^{-27}$ |
| *BGLAP* | 1 | 156.24 | -0.28 | $3 \times 10^{-27}$ |
| *PATE4* | 11 | 125.83 | -0.27 | $3 \times 10^{-27}$ |
| *COL6A3* | 2 | 237.32 | -0.27 | $4 \times 10^{-27}$ |
| *NRXN3* | 14 | 78.17 | -0.27 | $5 \times 10^{-27}$ |
| *NOTCH3* | 19 | 15.16 | 0.27 | $6 \times 10^{-27}$ |
| *ADH4* | 4 | 99.12 | -0.27 | $6 \times 10^{-27}$ |
| *ASTL* | 2 | 96.12 | -0.28 | $1 \times 10^{-26}$ |
| *BTG2* | 1 | 203.31 | -0.26 | $1 \times 10^{-26}$ |
| *C1orf43* | 1 | 154.21 | -0.27 | $2 \times 10^{-26}$ |
| *CDKN2C* | 1 | 50.96 | -0.26 | $2 \times 10^{-26}$ |
| *HTRA1* | 10 | 122.46 | -0.27 | $4 \times 10^{-26}$ |
| *GRID2* | 4 | 92.30 | -0.26 | $4 \times 10^{-26}$ |
| *SLAMF7* | 1 | 160.74 | -0.26 | $5 \times 10^{-26}$ |
| *DYRK3* | 1 | 206.64 | -0.26 | $8 \times 10^{-26}$ |
| *ASGR2* | 17 | 7.10 | -0.26 | $1 \times 10^{-25}$ |
| *LYSMD3* | 5 | 90.52 | -0.26 | $2 \times 10^{-25}$ |
| *TINAGL1* | 1 | 31.58 | -0.27 | $4 \times 10^{-25}$ |
| *BTNL9* | 5 | 181.04 | -0.26 | $4 \times 10^{-25}$ |
| *ANKRD54* | 22 | 37.83 | -0.25 | $5 \times 10^{-25}$ |
| *TRIAP1* | 12 | 120.44 | -0.25 | $6 \times 10^{-25}$ |
| *FERMT3* | 11 | 64.21 | -0.25 | $6 \times 10^{-25}$ |
| *GDF11* | 12 | 55.74 | -0.29 | $7 \times 10^{-25}$ |
| *SYT2* | 1 | 202.59 | -0.25 | $1 \times 10^{-24}$ |
| *GZMK* | 5 | 55.02 | -0.25 | $2 \times 10^{-24}$ |
| *AXIN2* | 17 | 65.53 | -0.26 | $2 \times 10^{-24}$ |
| *PSG6* | 19 | 42.90 | -0.25 | $2 \times 10^{-24}$ |
| *LILRB3* | 19 | 54.22 | -0.25 | $3 \times 10^{-24}$ |
| *KLRG2* | 7 | 139.45 | -0.25 | $4 \times 10^{-24}$ |
| *OLFML3* | 1 | 113.98 | -0.24 | $5 \times 10^{-24}$ |
| *HPD* | 12 | 121.84 | -0.25 | $5 \times 10^{-24}$ |
| *COL8A1* | 3 | 99.64 | -0.25 | $5 \times 10^{-24}$ |
| *CCNB1IP1* | 14 | 20.31 | -0.26 | $6 \times 10^{-24}$ |
| *PCDHA7* | 5 | 140.83 | -0.25 | $6 \times 10^{-24}$ |
| *RCAN1* | 21 | 34.51 | -0.24 | $8 \times 10^{-24}$ |
| *PLA2G10* | 16 | 14.67 | -0.27 | $1 \times 10^{-23}$ |
| *DYNLT1* | 6 | 158.64 | -0.28 | $1 \times 10^{-23}$ |
| *LAMP1* | 13 | 113.30 | -0.24 | $2 \times 10^{-23}$ |
| *ST3GAL5* | 2 | 85.84 | -0.24 | $2 \times 10^{-23}$ |
| *SPN* | 16 | 29.66 | -0.24 | $2 \times 10^{-23}$ |
| *ITGA5* | 12 | 54.40 | -0.26 | $3 \times 10^{-23}$ |
| *ALDH3B1* | 11 | 68.01 | -0.27 | $3 \times 10^{-23}$ |
| *DEFB108B* | 11 | 71.83 | -0.24 | $4 \times 10^{-23}$ |
| *IDO1* | 8 | 39.90 | -0.24 | $4 \times 10^{-23}$ |
| *ADPRH* | 3 | 119.58 | -0.24 | $5 \times 10^{-23}$ |
| *S100A7* | 1 | 153.46 | -0.24 | $5 \times 10^{-23}$ |
| *CD99L2* | X | 150.77 | -0.26 | $8 \times 10^{-23}$ |
| *POLR1C* | 6 | 43.51 | -0.24 | $9 \times 10^{-23}$ |
| *RPS19* | 19 | 41.86 | -0.24 | $9 \times 10^{-23}$ |
| *CRYGS* | 3 | 186.54 | -0.26 | $1 \times 10^{-22}$ |
| *RPS3* | 11 | 75.40 | -0.28 | $1 \times 10^{-22}$ |
| *CARD9* | 9 | 136.36 | -0.24 | $1 \times 10^{-22}$ |
| *IFNA5* | 9 | 21.30 | -0.24 | $1 \times 10^{-22}$ |
| *DEFB110* | 6 | 50.01 | -0.24 | $1 \times 10^{-22}$ |

**Table S3.** Association of HLA-specific *trans*-scores and SLE *(continued)*

| Gene symbol | Chr | Start position (Mb) | Log OR | P |
|---|---|---|---|---|
| *FRRS1L* | 9 | 109.13 | -0.24 | $1 \times 10^{-22}$ |
| *INS* | 11 | 2.16 | -0.24 | $1 \times 10^{-22}$ |
| *GBP1* | 1 | 89.05 | -0.25 | $2 \times 10^{-22}$ |
| *CPT1B* | 22 | 50.57 | -0.24 | $2 \times 10^{-22}$ |
| *CD79B* | 17 | 63.93 | -0.24 | $2 \times 10^{-22}$ |
| *FST* | 5 | 53.48 | -0.25 | $2 \times 10^{-22}$ |
| *CALB2* | 16 | 71.36 | -0.24 | $3 \times 10^{-22}$ |
| *TREM2* | 6 | 41.16 | -0.24 | $3 \times 10^{-22}$ |
| *PECR* | 2 | 216.00 | -0.23 | $3 \times 10^{-22}$ |
| *BDH2* | 4 | 103.08 | -0.24 | $4 \times 10^{-22}$ |
| *CRISPLD2* | 16 | 84.82 | -0.24 | $4 \times 10^{-22}$ |
| *CLEC4A* | 12 | 8.12 | -0.26 | $6 \times 10^{-22}$ |
| *NRBP1* | 2 | 27.43 | -0.24 | $9 \times 10^{-22}$ |
| *SF1* | 11 | 64.76 | 0.25 | $9 \times 10^{-22}$ |
| *ACBD7* | 10 | 15.08 | -0.24 | $1 \times 10^{-21}$ |
| *HDAC8* | X | 72.33 | -0.23 | $1 \times 10^{-21}$ |
| *CSF1R* | 5 | 150.05 | -0.23 | $1 \times 10^{-21}$ |
| *PTPN9* | 15 | 75.46 | -0.23 | $2 \times 10^{-21}$ |
| *DLL1* | 6 | 170.28 | 0.25 | $2 \times 10^{-21}$ |
| *MAMDC2* | 9 | 70.04 | -0.24 | $2 \times 10^{-21}$ |
| *MYZAP* | 15 | 57.59 | -0.23 | $2 \times 10^{-21}$ |
| *SDCBP2* | 20 | 1.31 | -0.24 | $2 \times 10^{-21}$ |
| *CD55* | 1 | 207.32 | -0.23 | $4 \times 10^{-21}$ |
| *ELANE* | 19 | 0.85 | 0.25 | $4 \times 10^{-21}$ |
| *SNAI2* | 8 | 48.92 | -0.23 | $5 \times 10^{-21}$ |
| *TGM3* | 20 | 2.30 | 0.24 | $5 \times 10^{-21}$ |
| *CAV2* | 7 | 116.29 | -0.23 | $9 \times 10^{-21}$ |
| *NSFL1C* | 20 | 1.44 | 0.25 | $9 \times 10^{-21}$ |
| *SPAST* | 2 | 32.06 | -0.23 | $1 \times 10^{-20}$ |
| *SLCO5A1* | 8 | 69.67 | -0.24 | $1 \times 10^{-20}$ |
| *C4BPA* | 1 | 207.10 | 0.25 | $1 \times 10^{-20}$ |
| *GABBR2* | 9 | 98.29 | -0.23 | $2 \times 10^{-20}$ |
| *TCEAL8* | X | 103.25 | -0.25 | $2 \times 10^{-20}$ |
| *CTRL* | 16 | 67.93 | -0.27 | $2 \times 10^{-20}$ |
| *LPCAT2* | 16 | 55.51 | -0.22 | $3 \times 10^{-20}$ |
| *CCDC25* | 8 | 27.73 | -0.22 | $3 \times 10^{-20}$ |
| *RAB6B* | 3 | 133.82 | 0.23 | $3 \times 10^{-20}$ |
| *FARS2* | 6 | 5.26 | -0.23 | $5 \times 10^{-20}$ |
| *SPATA31D4* | 9 | 81.93 | -0.23 | $6 \times 10^{-20}$ |
| *PGK1* | X | 77.91 | -0.22 | $8 \times 10^{-20}$ |
| *HERPUD2* | 7 | 35.63 | -0.23 | $1 \times 10^{-19}$ |
| *OLFML1* | 11 | 7.49 | -0.22 | $1 \times 10^{-19}$ |
| *ZNF134* | 19 | 57.61 | -0.22 | $1 \times 10^{-19}$ |
| *SEC61B* | 9 | 99.22 | -0.23 | $1 \times 10^{-19}$ |
| *MX1* | 21 | 41.42 | -0.24 | $2 \times 10^{-19}$ |
| *DCK* | 4 | 70.99 | -0.22 | $2 \times 10^{-19}$ |
| *CD163* | 12 | 7.47 | -0.22 | $2 \times 10^{-19}$ |
| *UBE2C* | 20 | 45.81 | -0.22 | $2 \times 10^{-19}$ |
| *VAMP4* | 1 | 171.70 | -0.22 | $3 \times 10^{-19}$ |
| *TTN* | 2 | 178.53 | -0.22 | $3 \times 10^{-19}$ |
| *MUCL1* | 12 | 54.83 | -0.22 | $3 \times 10^{-19}$ |
| *LRRTM4* | 2 | 76.75 | -0.22 | $3 \times 10^{-19}$ |
| *BCL2L11* | 2 | 111.12 | -0.22 | $3 \times 10^{-19}$ |
| *RCVRN* | 17 | 9.90 | -0.21 | $4 \times 10^{-19}$ |

**Table S3.** Association of HLA-specific *trans*-scores and SLE *(continued)*

| Gene symbol | Chr | Start position (Mb) | Log OR | P |
|---|---|---|---|---|
| *MRAP* | 21 | 32.29 | 0.24 | $4 \times 10^{-19}$ |
| *MOCS3* | 20 | 50.96 | -0.22 | $4 \times 10^{-19}$ |
| *BMP3* | 4 | 81.03 | -0.22 | $5 \times 10^{-19}$ |
| *APOA4* | 11 | 116.82 | -0.29 | $1 \times 10^{-28}$ |
| *PLSCR3* | 17 | 7.39 | -0.21 | $6 \times 10^{-19}$ |
| *IL5* | 5 | 132.54 | -0.21 | $6 \times 10^{-19}$ |
| *SMCO2* | 12 | 27.45 | -0.22 | $6 \times 10^{-19}$ |
| *BACH2* | 6 | 89.93 | -0.23 | $8 \times 10^{-19}$ |
| *VSIG2* | 11 | 124.75 | -0.21 | $8 \times 10^{-19}$ |
| *SEMA4A* | 1 | 156.15 | -0.22 | $9 \times 10^{-19}$ |
| *LYPD1* | 2 | 132.64 | -0.21 | $9 \times 10^{-19}$ |
| *CCS* | 11 | 66.59 | -0.24 | $9 \times 10^{-19}$ |
| *HRK* | 12 | 116.86 | -0.21 | $1 \times 10^{-18}$ |
| *AIFM1* | X | 130.12 | -0.22 | $1 \times 10^{-18}$ |
| *BARD1* | 2 | 214.73 | -0.21 | $1 \times 10^{-18}$ |
| *KRT7* | 12 | 52.23 | -0.21 | $1 \times 10^{-18}$ |
| *RAB17* | 2 | 237.57 | -0.21 | $1 \times 10^{-18}$ |
| *SUN3* | 7 | 47.99 | -0.21 | $1 \times 10^{-18}$ |
| *ITGB5* | 3 | 124.76 | -0.22 | $2 \times 10^{-18}$ |
| *NADK* | 1 | 1.75 | -0.22 | $2 \times 10^{-18}$ |
| *PCDHGB1* | 5 | 141.35 | -0.21 | $2 \times 10^{-18}$ |
| *PRIM1* | 12 | 56.73 | -0.23 | $2 \times 10^{-18}$ |
| *MSH2* | 2 | 47.40 | -0.22 | $3 \times 10^{-18}$ |
| *MTMR1* | X | 150.69 | -0.21 | $3 \times 10^{-18}$ |
| *C1GALT1C1* | X | 120.63 | -0.21 | $3 \times 10^{-18}$ |
| *MTCP1* | X | 155.06 | -0.21 | $3 \times 10^{-18}$ |
| *PLBD2* | 12 | 113.36 | -0.21 | $4 \times 10^{-18}$ |
| *NR5A2* | 1 | 200.03 | -0.22 | $4 \times 10^{-18}$ |
| *BCAP29* | 7 | 107.58 | -0.21 | $4 \times 10^{-18}$ |
| *ZDHHC14* | 6 | 157.38 | -0.21 | $4 \times 10^{-18}$ |
| *SIRPB1* | 20 | 1.56 | -0.21 | $5 \times 10^{-18}$ |
| *CFHR4* | 1 | 196.89 | -0.23 | $6 \times 10^{-18}$ |
| *FAM162A* | 3 | 122.38 | -0.21 | $6 \times 10^{-18}$ |
| *ARG1* | 6 | 131.47 | 0.23 | $8 \times 10^{-18}$ |
| *PCSK1* | 5 | 96.39 | -0.21 | $9 \times 10^{-18}$ |
| *FABP1* | 2 | 88.12 | -0.21 | $9 \times 10^{-18}$ |
| *LRIT3* | 4 | 109.85 | -0.22 | $9 \times 10^{-18}$ |
| *PDCL2* | 4 | 55.56 | -0.21 | $1 \times 10^{-17}$ |
| *UNC93B1* | 11 | 67.99 | -0.23 | $1 \times 10^{-17}$ |
| *WIF1* | 12 | 65.05 | -0.25 | $2 \times 10^{-17}$ |
| *LAYN* | 11 | 111.54 | 0.23 | $2 \times 10^{-17}$ |
| *COL11A2* | 6 | 33.16 | -0.22 | $2 \times 10^{-17}$ |
| *PBK* | 8 | 27.81 | -0.20 | $2 \times 10^{-17}$ |
| *OTOR* | 20 | 16.75 | -0.20 | $3 \times 10^{-17}$ |
| *GALNT2* | 1 | 230.06 | 0.24 | $1 \times 10^{-19}$ |
| *EIF4G3* | 1 | 20.81 | -0.20 | $3 \times 10^{-17}$ |
| *SAP30* | 4 | 173.37 | -0.20 | $3 \times 10^{-17}$ |
| *CDC42BPA* | 1 | 226.99 | -0.21 | $4 \times 10^{-17}$ |
| *CPOX* | 3 | 98.58 | -0.21 | $4 \times 10^{-17}$ |
| *SH2D3C* | 9 | 127.74 | -0.21 | $5 \times 10^{-17}$ |
| *CSGALNACT2* | 10 | 43.14 | -0.21 | $5 \times 10^{-17}$ |
| *MPP7* | 10 | 28.05 | -0.20 | $6 \times 10^{-17}$ |
| *PDIA4* | 7 | 149.00 | -0.20 | $7 \times 10^{-17}$ |
| *TMPRSS6* | 22 | 37.07 | -0.20 | $7 \times 10^{-17}$ |

**Table S3.** Association of HLA-specific *trans*-scores and SLE *(continued)*

| Gene symbol | Chr | Start position (Mb) | Log OR | P |
|---|---|---|---|---|
| *PTPRS* | 19 | 5.16 | -0.20 | $8 \times 10^{-17}$ |
| *ALCAM* | 3 | 105.37 | 0.28 | $3 \times 10^{-28}$ |
| *PTCHD3* | 10 | 27.40 | -0.20 | $8 \times 10^{-17}$ |
| *CTNNA2* | 2 | 79.19 | -0.21 | $8 \times 10^{-17}$ |
| *RMI1* | 9 | 83.98 | -0.20 | $8 \times 10^{-17}$ |
| *COLEC10* | 8 | 119.00 | -0.21 | $9 \times 10^{-17}$ |
| *MANBA* | 4 | 102.63 | -0.20 | $9 \times 10^{-17}$ |
| *NOTCH2* | 1 | 119.91 | -0.20 | $9 \times 10^{-17}$ |
| *CCNB2* | 15 | 59.11 | -0.20 | $9 \times 10^{-17}$ |
| *VNN2* | 6 | 132.74 | 0.20 | $1 \times 10^{-16}$ |
| *HINT2* | 9 | 35.81 | -0.20 | $1 \times 10^{-16}$ |
| *LPO* | 17 | 58.22 | -0.22 | $1 \times 10^{-16}$ |
| *TOR1AIP1* | 1 | 179.88 | -0.20 | $1 \times 10^{-16}$ |
| *PARP1* | 1 | 226.36 | -0.20 | $1 \times 10^{-16}$ |
| *VCAM1* | 1 | 100.72 | -0.23 | $1 \times 10^{-16}$ |
| *MCTS1* | X | 120.59 | -0.21 | $1 \times 10^{-16}$ |
| *SLC27A2* | 15 | 50.18 | -0.22 | $2 \times 10^{-16}$ |
| *PPP1R3B* | 8 | 9.14 | -0.22 | $2 \times 10^{-16}$ |
| *HEPACAM2* | 7 | 93.19 | -0.20 | $2 \times 10^{-16}$ |
| *ZNF23* | 16 | 71.45 | -0.20 | $2 \times 10^{-16}$ |
| *COL3A1* | 2 | 188.97 | -0.20 | $2 \times 10^{-16}$ |
| *SLC3A1* | 2 | 44.28 | 0.25 | $2 \times 10^{-16}$ |
| *PZP* | 12 | 9.15 | -0.20 | $2 \times 10^{-16}$ |
| *PRRG1* | X | 37.35 | -0.20 | $2 \times 10^{-16}$ |
| *RABEPK* | 9 | 125.20 | -0.20 | $3 \times 10^{-16}$ |
| *PRDM1* | 6 | 105.99 | -0.19 | $3 \times 10^{-16}$ |
| *RRM2* | 2 | 10.12 | -0.20 | $3 \times 10^{-16}$ |
| *LY9* | 1 | 160.80 | -0.20 | $2 \times 10^{-16}$ |
| *APOC2* | 19 | 44.95 | -0.23 | $3 \times 10^{-16}$ |
| *PTK2B* | 8 | 27.31 | -0.20 | $3 \times 10^{-16}$ |
| *ARHGAP36* | X | 131.06 | -0.19 | $3 \times 10^{-16}$ |
| *TMX2* | 11 | 57.71 | -0.22 | $2 \times 10^{-16}$ |
| *COL1A1* | 17 | 50.18 | -0.20 | $2 \times 10^{-16}$ |
| *PROK2* | 3 | 71.77 | -0.20 | $5 \times 10^{-16}$ |
| *ZNF276* | 16 | 89.72 | -0.20 | $5 \times 10^{-16}$ |
| *ENO3* | 17 | 4.95 | -0.19 | $4 \times 10^{-16}$ |
| *VAMP1* | 12 | 6.46 | -0.19 | $5 \times 10^{-16}$ |
| *NACA* | 12 | 56.71 | -0.20 | $3 \times 10^{-16}$ |
| *CDH7* | 18 | 65.75 | -0.20 | $5 \times 10^{-16}$ |
| *DNER* | 2 | 229.36 | 0.21 | $6 \times 10^{-16}$ |
| *POLE2* | 14 | 49.64 | 0.21 | $6 \times 10^{-16}$ |
| *CCM2* | 7 | 45.00 | 0.21 | $6 \times 10^{-16}$ |
| *ANKRD27* | 19 | 32.60 | -0.19 | $7 \times 10^{-16}$ |
| *SENP7* | 3 | 101.32 | -0.19 | $7 \times 10^{-16}$ |
| *STAU2* | 8 | 73.42 | -0.19 | $6 \times 10^{-16}$ |
| *PPP1R8* | 1 | 27.83 | 0.21 | $6 \times 10^{-16}$ |
| *LYPD3* | 19 | 43.46 | -0.21 | $6 \times 10^{-16}$ |
| *ANKRD1* | 10 | 90.91 | 0.21 | $6 \times 10^{-16}$ |
| *ADH1C* | 4 | 99.34 | 0.21 | $6 \times 10^{-16}$ |
| *EPHA7* | 6 | 93.24 | 0.21 | $6 \times 10^{-16}$ |
| *PLA2G2D* | 1 | 20.11 | 0.21 | $6 \times 10^{-16}$ |
| *SNPH* | 20 | 1.27 | 0.21 | $6 \times 10^{-16}$ |
| *NAIF1* | 9 | 128.06 | 0.21 | $6 \times 10^{-16}$ |
| *RPS5* | 19 | 58.39 | 0.21 | $6 \times 10^{-16}$ |

**Table S3.** Association of HLA-specific *trans*-scores and SLE *(continued)*

| Gene symbol | Chr | Start position (Mb) | Log OR | P |
|---|---|---|---|---|
| *CCL3L1* | 17 | 36.19 | 0.21 | $6 \times 10^{-16}$ |
| *TPSG1* | 16 | 1.22 | -0.20 | $7 \times 10^{-16}$ |
| *PLXNC1* | 12 | 94.15 | 0.21 | $6 \times 10^{-16}$ |
| *TFF3* | 21 | 42.31 | 0.21 | $6 \times 10^{-16}$ |
| *BMPR2* | 2 | 202.38 | 0.21 | $6 \times 10^{-16}$ |
| *ART4* | 12 | 14.83 | -0.20 | $7 \times 10^{-16}$ |
| *TRAPPC5* | 19 | 7.68 | 0.21 | $6 \times 10^{-16}$ |
| *COX6C* | 8 | 99.87 | 0.21 | $6 \times 10^{-16}$ |
| *TMCC3* | 12 | 94.57 | 0.21 | $6 \times 10^{-16}$ |
| *MRPL21* | 11 | 68.89 | 0.21 | $6 \times 10^{-16}$ |
| *RSRP1* | 1 | 25.24 | 0.21 | $6 \times 10^{-16}$ |
| *NXT1* | 20 | 23.35 | 0.21 | $6 \times 10^{-16}$ |
| *SPINT1* | 15 | 40.84 | 0.20 | $1 \times 10^{-15}$ |
| *TNFRSF10B* | 8 | 23.02 | -0.20 | $1 \times 10^{-15}$ |
| *SSR1* | 6 | 7.27 | -0.20 | $1 \times 10^{-15}$ |
| *USP25* | 21 | 15.73 | -0.19 | $1 \times 10^{-15}$ |
| *SORBS3* | 8 | 22.54 | -0.19 | $1 \times 10^{-15}$ |
| *PIK3C2A* | 11 | 17.08 | -0.19 | $1 \times 10^{-15}$ |
| *EBI3* | 19 | 4.23 | -0.19 | $2 \times 10^{-15}$ |
| *PGK2* | 6 | 49.79 | -0.19 | $1 \times 10^{-15}$ |
| *EPDR1* | 7 | 37.92 | -0.19 | $2 \times 10^{-15}$ |
| *ZNF180* | 19 | 44.47 | -0.21 | $2 \times 10^{-15}$ |
| *RARRES2* | 7 | 150.34 | 0.22 | $2 \times 10^{-15}$ |
| *ANKRD46* | 8 | 100.51 | -0.23 | $2 \times 10^{-15}$ |
| *UMOD* | 16 | 20.33 | -0.19 | $2 \times 10^{-15}$ |
| *CHST5* | 16 | 75.53 | -0.20 | $2 \times 10^{-15}$ |
| *PCK1* | 20 | 57.56 | -0.19 | $2 \times 10^{-15}$ |
| *LTBR* | 12 | 6.38 | 0.20 | $2 \times 10^{-15}$ |
| *FAM171A1* | 10 | 15.21 | -0.20 | $2 \times 10^{-15}$ |
| *PRTN3* | 19 | 0.84 | -0.19 | $3 \times 10^{-15}$ |
| *TMEM108* | 3 | 133.04 | -0.20 | $3 \times 10^{-15}$ |
| *CD1A* | 1 | 158.25 | -0.20 | $3 \times 10^{-15}$ |
| *TIMELESS* | 12 | 56.42 | -0.21 | $3 \times 10^{-15}$ |
| *PRND* | 20 | 4.72 | -0.19 | $4 \times 10^{-15}$ |
| *KAT2B* | 3 | 20.04 | -0.19 | $4 \times 10^{-15}$ |
| *MMP2* | 16 | 55.39 | -0.19 | $4 \times 10^{-15}$ |
| *SNAPIN* | 1 | 153.66 | -0.20 | $4 \times 10^{-15}$ |
| *PIK3R1* | 5 | 68.22 | -0.23 | $4 \times 10^{-15}$ |
| *CLEC2D* | 12 | 9.66 | -0.19 | $5 \times 10^{-15}$ |
| *HIBADH* | 7 | 27.53 | -0.19 | $5 \times 10^{-15}$ |
| *GFRAL* | 6 | 55.33 | -0.23 | $5 \times 10^{-15}$ |
| *DNAJC18* | 5 | 139.41 | -0.19 | $5 \times 10^{-15}$ |
| *CAND1* | 12 | 67.27 | -0.19 | $5 \times 10^{-15}$ |
| *ATP2A3* | 17 | 3.92 | -0.20 | $6 \times 10^{-15}$ |
| *SCP2D1* | 20 | 18.81 | -0.19 | $6 \times 10^{-15}$ |
| *ROBO1* | 3 | 78.60 | 0.20 | $6 \times 10^{-15}$ |
| *LRTM1* | 3 | 54.92 | -0.19 | $7 \times 10^{-15}$ |
| *PYCR2* | 1 | 225.92 | -0.19 | $7 \times 10^{-15}$ |
| *DNASE2B* | 1 | 84.40 | -0.19 | $7 \times 10^{-15}$ |
| *LRRC32* | 11 | 76.66 | -0.22 | $7 \times 10^{-15}$ |
| *EPHB3* | 3 | 184.56 | -0.19 | $7 \times 10^{-15}$ |
| *RGS1* | 1 | 192.58 | -0.19 | $9 \times 10^{-15}$ |
| *BECN1* | 17 | 42.81 | -0.18 | $1 \times 10^{-14}$ |
| *MBTPS1* | 16 | 84.05 | -0.19 | $1 \times 10^{-14}$ |

**Table S3.** Association of HLA-specific *trans*-scores and SLE *(continued)*

| Gene symbol | Chr | Start position (Mb) | Log OR | P |
|---|---|---|---|---|
| *NDUFB11* | X | 47.14 | -0.19 | $1 \times 10^{-14}$ |
| *SPINK7* | 5 | 148.31 | -0.19 | $1 \times 10^{-14}$ |
| *CD207* | 2 | 70.83 | -0.20 | $1 \times 10^{-14}$ |
| *MINPP1* | 10 | 87.50 | -0.19 | $1 \times 10^{-14}$ |
| *LRTM2* | 12 | 1.82 | -0.19 | $1 \times 10^{-14}$ |
| *ATP1B1* | 1 | 169.11 | -0.19 | $1 \times 10^{-14}$ |
| *ITSN1* | 21 | 33.64 | -0.20 | $1 \times 10^{-14}$ |
| *UBE2D3* | 4 | 102.79 | 0.20 | $1 \times 10^{-14}$ |
| *CSDC2* | 22 | 41.56 | -0.19 | $1 \times 10^{-14}$ |
| *SPATA9* | 5 | 95.65 | -0.19 | $1 \times 10^{-14}$ |
| *BTLA* | 3 | 112.46 | -0.19 | $1 \times 10^{-14}$ |
| *FIG4* | 6 | 109.69 | -0.18 | $2 \times 10^{-14}$ |
| *RPL30* | 8 | 98.02 | -0.18 | $2 \times 10^{-14}$ |
| *LCP1* | 13 | 46.13 | -0.19 | $2 \times 10^{-14}$ |
| *CCL7* | 17 | 34.27 | -0.20 | $2 \times 10^{-14}$ |
| *RPS6KB1* | 17 | 59.89 | -0.29 | $2 \times 10^{-14}$ |
| *RGS4* | 1 | 163.07 | -0.19 | $2 \times 10^{-14}$ |
| *CA2* | 8 | 85.46 | -0.19 | $2 \times 10^{-14}$ |
| *PIAS3* | 1 | 145.85 | -0.19 | $2 \times 10^{-14}$ |
| *PDLIM4* | 5 | 132.26 | -0.19 | $2 \times 10^{-14}$ |
| *RNF114* | 20 | 49.94 | -0.19 | $2 \times 10^{-14}$ |
| *ENTPD1* | 10 | 95.71 | 0.20 | $3 \times 10^{-14}$ |
| *RHOB* | 2 | 20.45 | -0.18 | $3 \times 10^{-14}$ |
| *MGA* | 15 | 41.62 | -0.18 | $3 \times 10^{-14}$ |
| *NGFR* | 17 | 49.50 | -0.18 | $3 \times 10^{-14}$ |
| *ITLN1* | 1 | 160.88 | -0.21 | $3 \times 10^{-14}$ |
| *COPE* | 19 | 18.90 | -0.20 | $4 \times 10^{-14}$ |
| *RPS3A* | 4 | 151.10 | 0.18 | $4 \times 10^{-14}$ |
| *EDIL3* | 5 | 83.94 | -0.18 | $6 \times 10^{-14}$ |
| *FGG* | 4 | 154.60 | -0.21 | $6 \times 10^{-14}$ |
| *ITM2C* | 2 | 230.86 | -0.18 | $6 \times 10^{-14}$ |
| *TPST1* | 7 | 66.21 | -0.18 | $7 \times 10^{-14}$ |
| *CEACAM20* | 19 | 44.50 | -0.18 | $7 \times 10^{-14}$ |
| *PDGFC* | 4 | 156.76 | -0.18 | $7 \times 10^{-14}$ |
| *MYB* | 6 | 135.18 | -0.18 | $7 \times 10^{-14}$ |
| *ADM* | 11 | 10.31 | -0.18 | $8 \times 10^{-14}$ |
| *BAG2* | 6 | 57.17 | -0.19 | $8 \times 10^{-14}$ |
| *MAGEA6* | X | 152.77 | -0.18 | $9 \times 10^{-14}$ |
| *IGFBPL1* | 9 | 38.41 | -0.18 | $9 \times 10^{-14}$ |
| *CXCL11* | 4 | 76.03 | -0.18 | $9 \times 10^{-14}$ |
| *AMBP* | 9 | 114.06 | -0.19 | $9 \times 10^{-14}$ |
| *PLXNA1* | 3 | 126.98 | -0.18 | $1 \times 10^{-13}$ |
| *EPHB2* | 1 | 22.71 | -0.17 | $1 \times 10^{-13}$ |
| *TP53* | 17 | 7.66 | -0.18 | $1 \times 10^{-13}$ |
| *CYTIP* | 2 | 157.41 | -0.18 | $1 \times 10^{-13}$ |
| *RBL2* | 16 | 53.43 | -0.18 | $1 \times 10^{-13}$ |
| *NLRP4* | 19 | 55.84 | -0.18 | $1 \times 10^{-13}$ |
| *HHIP* | 4 | 144.65 | -0.18 | $1 \times 10^{-13}$ |
| *FUT10* | 8 | 33.37 | -0.19 | $1 \times 10^{-13}$ |
| *FOLR2* | 11 | 72.22 | -0.29 | $3 \times 10^{-18}$ |
| *DTX1* | 12 | 113.06 | -0.18 | $2 \times 10^{-13}$ |
| *FAM172A* | 5 | 93.62 | -0.18 | $2 \times 10^{-13}$ |
| *PTHLH* | 12 | 27.96 | -0.18 | $2 \times 10^{-13}$ |
| *TMEM106B* | 7 | 12.21 | -0.18 | $2 \times 10^{-13}$ |

**Table S3.** Association of HLA-specific *trans*-scores and SLE *(continued)*

| Gene symbol | Chr | Start position (Mb) | Log OR | P |
|---|---|---|---|---|
| *KLK3* | 19 | 50.85 | -0.18 | $2 \times 10^{-13}$ |
| *FCN1* | 9 | 134.90 | -0.18 | $2 \times 10^{-13}$ |
| *MRRF* | 9 | 122.26 | -0.18 | $2 \times 10^{-13}$ |
| *SYNCRIP* | 6 | 85.61 | 0.19 | $2 \times 10^{-13}$ |
| *GKN2* | 2 | 68.95 | -0.18 | $3 \times 10^{-13}$ |
| *CHST6* | 16 | 75.47 | -0.18 | $3 \times 10^{-13}$ |
| *HSD17B1* | 17 | 42.55 | -0.18 | $3 \times 10^{-13}$ |
| *IRF1* | 5 | 132.44 | -0.18 | $3 \times 10^{-13}$ |
| *FAM210A* | 18 | 13.66 | -0.17 | $4 \times 10^{-13}$ |
| *ZP4* | 1 | 237.88 | -0.17 | $4 \times 10^{-13}$ |
| *IGFL3* | 19 | 46.12 | -0.17 | $4 \times 10^{-13}$ |
| *NBL1* | 1 | 19.60 | -0.17 | $4 \times 10^{-13}$ |
| *BPHL* | 6 | 3.12 | -0.17 | $4 \times 10^{-13}$ |
| *PILRA* | 7 | 100.37 | -0.17 | $5 \times 10^{-13}$ |
| *HS3ST4* | 16 | 25.69 | -0.17 | $5 \times 10^{-13}$ |
| *TNMD* | X | 100.58 | -0.19 | $6 \times 10^{-13}$ |
| *IL2* | 4 | 122.45 | -0.18 | $6 \times 10^{-13}$ |
| *EXOSC3* | 9 | 37.76 | -0.18 | $7 \times 10^{-13}$ |
| *CFHR3* | 1 | 196.77 | -0.18 | $7 \times 10^{-13}$ |
| *SPRED1* | 15 | 38.25 | -0.17 | $7 \times 10^{-13}$ |
| *HSPH1* | 13 | 31.13 | -0.17 | $7 \times 10^{-13}$ |
| *CD69* | 12 | 9.75 | 0.21 | $8 \times 10^{-13}$ |
| *CDC25B* | 20 | 3.79 | -0.18 | $8 \times 10^{-13}$ |
| *CHEK1* | 11 | 125.63 | -0.17 | $8 \times 10^{-13}$ |
| *ACAN* | 15 | 88.80 | -0.18 | $8 \times 10^{-13}$ |
| *CANT1* | 17 | 78.99 | -0.17 | $1 \times 10^{-12}$ |
| *OLR1* | 12 | 10.16 | -0.17 | $1 \times 10^{-12}$ |
| *S100A14* | 1 | 153.61 | -0.17 | $1 \times 10^{-12}$ |
| *ACVR2B* | 3 | 38.45 | -0.18 | $1 \times 10^{-12}$ |
| *LCORL* | 4 | 17.84 | -0.17 | $1 \times 10^{-12}$ |
| *GFAP* | 17 | 44.90 | -0.17 | $1 \times 10^{-12}$ |
| *GALNT1* | 18 | 35.58 | -0.17 | $1 \times 10^{-12}$ |
| *MZF1* | 19 | 58.56 | -0.17 | $1 \times 10^{-12}$ |
| *HECW1* | 7 | 43.11 | -0.18 | $2 \times 10^{-12}$ |
| *MAP2K6* | 17 | 69.41 | -0.18 | $2 \times 10^{-12}$ |
| *BRAT1* | 7 | 2.54 | -0.17 | $2 \times 10^{-12}$ |
| *ZPBP* | 7 | 49.85 | -0.17 | $2 \times 10^{-12}$ |
| *PLG* | 6 | 160.70 | -0.17 | $2 \times 10^{-12}$ |
| *TXN* | 9 | 110.24 | -0.18 | $2 \times 10^{-12}$ |
| *CYB561D1* | 1 | 109.49 | -0.17 | $2 \times 10^{-12}$ |
| *PCDHB1* | 5 | 141.05 | -0.17 | $2 \times 10^{-12}$ |
| *SPINK5* | 5 | 148.03 | 0.19 | $2 \times 10^{-12}$ |
| *DEFB106A* | 8 | 7.83 | -0.18 | $2 \times 10^{-12}$ |
| *EXTL2* | 1 | 100.87 | -0.24 | $2 \times 10^{-12}$ |
| *LRRN1* | 3 | 3.80 | -0.17 | $2 \times 10^{-12}$ |
| *MASP1* | 3 | 187.22 | -0.17 | $2 \times 10^{-12}$ |
| *VPS4B* | 18 | 63.39 | 0.17 | $2 \times 10^{-12}$ |
| *SNX1* | 15 | 64.09 | -0.17 | $2 \times 10^{-12}$ |
| *PRKAR1B* | 7 | 0.55 | -0.17 | $2 \times 10^{-12}$ |
| *DNAJC10* | 2 | 182.72 | -0.18 | $3 \times 10^{-12}$ |
| *C20orf173* | 20 | 35.52 | -0.17 | $3 \times 10^{-12}$ |
| *ATP1B4* | X | 120.36 | -0.17 | $3 \times 10^{-12}$ |
| *TNFRSF18* | 1 | 1.20 | -0.25 | $3 \times 10^{-12}$ |
| *PRLR* | 5 | 35.05 | 0.19 | $3 \times 10^{-12}$ |

**Table S3.** Association of HLA-specific *trans*-scores and SLE *(continued)*

| Gene symbol | Chr | Start position (Mb) | Log OR | P |
|---|---|---|---|---|
| *P4HA1* | 10 | 73.01 | -0.17 | $4 \times 10^{-12}$ |
| *OLFM3* | 1 | 101.80 | -0.16 | $4 \times 10^{-12}$ |
| *IRF9* | 14 | 24.16 | -0.17 | $4 \times 10^{-12}$ |
| *STAB1* | 3 | 52.50 | -0.17 | $4 \times 10^{-12}$ |
| *IL31RA* | 5 | 55.85 | -0.17 | $4 \times 10^{-12}$ |
| *CST8* | 20 | 23.49 | -0.24 | $4 \times 10^{-12}$ |
| *CAMK1* | 3 | 9.76 | -0.16 | $5 \times 10^{-12}$ |
| *ANXA8* | 10 | 47.46 | -0.18 | $5 \times 10^{-12}$ |
| *S100A9* | 1 | 153.36 | -0.18 | $5 \times 10^{-12}$ |
| *CELF2* | 10 | 10.80 | -0.17 | $5 \times 10^{-12}$ |
| *NODAL* | 10 | 70.43 | -0.18 | $6 \times 10^{-12}$ |
| *C1orf226* | 1 | 162.38 | -0.17 | $6 \times 10^{-12}$ |
| *IL7R* | 5 | 35.85 | -0.16 | $7 \times 10^{-12}$ |
| *IL20* | 1 | 206.87 | -0.18 | $7 \times 10^{-12}$ |
| *CDHR5* | 11 | 0.62 | -0.17 | $7 \times 10^{-12}$ |
| *PDE6D* | 2 | 231.73 | -0.17 | $7 \times 10^{-12}$ |
| *ARPC3* | 12 | 110.43 | -0.16 | $8 \times 10^{-12}$ |
| *RPRD1A* | 18 | 35.98 | -0.17 | $8 \times 10^{-12}$ |
| *LYPLAL1* | 1 | 219.17 | -0.17 | $8 \times 10^{-12}$ |
| *DEFA1* | 8 | 6.98 | -0.22 | $8 \times 10^{-12}$ |
| *IL34* | 16 | 70.58 | -0.18 | $9 \times 10^{-12}$ |
| *NEU1* | 6 | 31.86 | -0.17 | $9 \times 10^{-12}$ |
| *NUP210* | 3 | 13.32 | -0.16 | $1 \times 10^{-11}$ |
| *ARL8B* | 3 | 5.12 | -0.16 | $1 \times 10^{-11}$ |
| *LIF* | 22 | 30.24 | -0.16 | $1 \times 10^{-11}$ |
| *PFDN1* | 5 | 140.25 | -0.16 | $1 \times 10^{-11}$ |
| *KDSR* | 18 | 63.33 | -0.16 | $1 \times 10^{-11}$ |
| *KRT19* | 17 | 41.52 | -0.17 | $1 \times 10^{-11}$ |
| *MUTYH* | 1 | 45.33 | -0.17 | $1 \times 10^{-11}$ |
| *PSG11* | 19 | 43.01 | -0.16 | $1 \times 10^{-11}$ |
| *FBP2* | 9 | 94.56 | -0.16 | $2 \times 10^{-11}$ |
| *NEFH* | 22 | 29.48 | -0.16 | $2 \times 10^{-11}$ |
| *TJP1* | 15 | 29.70 | -0.17 | $2 \times 10^{-11}$ |
| *KIF1C* | 17 | 5.00 | -0.18 | $2 \times 10^{-11}$ |
| *AZGP1* | 7 | 99.97 | -0.16 | $2 \times 10^{-11}$ |
| *ERLIN1* | 10 | 100.15 | -0.16 | $2 \times 10^{-11}$ |
| *WBP2* | 17 | 75.85 | -0.17 | $2 \times 10^{-11}$ |
| *SIGLEC12* | 19 | 51.49 | -0.16 | $2 \times 10^{-11}$ |
| *KREMEN1* | 22 | 29.07 | -0.16 | $2 \times 10^{-11}$ |
| *BCHE* | 3 | 165.77 | -0.16 | $2 \times 10^{-11}$ |
| *SCN4B* | 11 | 118.13 | 0.18 | $2 \times 10^{-11}$ |
| *DNAJC11* | 1 | 6.63 | -0.16 | $2 \times 10^{-11}$ |
| *SOD2* | 6 | 159.67 | -0.16 | $2 \times 10^{-11}$ |
| *TMPRSS5* | 11 | 113.69 | -0.16 | $2 \times 10^{-11}$ |
| *CD274* | 9 | 5.45 | -0.17 | $3 \times 10^{-11}$ |
| *BUB1* | 2 | 110.64 | -0.17 | $3 \times 10^{-11}$ |
| *SCARA3* | 8 | 27.63 | -0.16 | $3 \times 10^{-11}$ |
| *SIGLEC8* | 19 | 51.45 | -0.18 | $3 \times 10^{-11}$ |
| *GOSR2* | 17 | 46.92 | -0.16 | $4 \times 10^{-11}$ |
| *ADH7* | 4 | 99.41 | -0.16 | $4 \times 10^{-11}$ |
| *MANSC1* | 12 | 12.33 | -0.16 | $4 \times 10^{-11}$ |
| *F11* | 4 | 186.27 | -0.16 | $4 \times 10^{-11}$ |
| *CD248* | 11 | 66.31 | -0.17 | $4 \times 10^{-11}$ |
| *EMC1* | 1 | 19.22 | -0.16 | $5 \times 10^{-11}$ |

**Table S3.** Association of HLA-specific *trans*-scores and SLE *(continued)*

| Gene symbol | Chr | Start position (Mb) | Log OR | P |
|---|---|---|---|---|
| *C1orf162* | 1 | 111.47 | -0.16 | $5 \times 10^{-11}$ |
| *SYT7* | 11 | 61.51 | -0.17 | $5 \times 10^{-11}$ |
| *CLTA* | 9 | 36.19 | -0.16 | $5 \times 10^{-11}$ |
| *CCNA1* | 13 | 36.43 | -0.16 | $5 \times 10^{-11}$ |
| *ATG3* | 3 | 112.53 | -0.16 | $6 \times 10^{-11}$ |
| *CD70* | 19 | 6.58 | -0.16 | $6 \times 10^{-11}$ |
| *NCMAP* | 1 | 24.56 | -0.16 | $7 \times 10^{-11}$ |
| *LCN10* | 9 | 136.74 | -0.16 | $7 \times 10^{-11}$ |
| *BST2* | 19 | 17.40 | -0.16 | $7 \times 10^{-11}$ |
| *IL17D* | 13 | 20.70 | -0.26 | $8 \times 10^{-11}$ |
| *PPM1D* | 17 | 60.60 | -0.16 | $8 \times 10^{-11}$ |
| *CDC25A* | 3 | 48.16 | -0.17 | $8 \times 10^{-11}$ |
| *DSTN* | 20 | 17.57 | -0.16 | $9 \times 10^{-11}$ |
| *SFTPD* | 10 | 79.94 | 0.16 | $9 \times 10^{-11}$ |
| *RNASE10* | 14 | 20.51 | -0.16 | $9 \times 10^{-11}$ |
| *OSBPL11* | 3 | 125.53 | -0.16 | $9 \times 10^{-11}$ |
| *IL17RC* | 3 | 9.92 | -0.18 | $1 \times 10^{-10}$ |
| *DUS2* | 16 | 67.99 | -0.16 | $1 \times 10^{-10}$ |
| *NCF4* | 22 | 36.86 | -0.15 | $1 \times 10^{-10}$ |
| *BCCIP* | 10 | 125.82 | -0.15 | $1 \times 10^{-10}$ |
| *NDRG4* | 16 | 58.46 | -0.15 | $1 \times 10^{-10}$ |
| *CTSE* | 1 | 206.01 | -0.15 | $1 \times 10^{-10}$ |
| *TACSTD2* | 1 | 58.58 | -0.15 | $1 \times 10^{-10}$ |
| *SATB2* | 2 | 199.27 | -0.15 | $2 \times 10^{-10}$ |
| *KIR2DL1* | 19 | 54.77 | -0.15 | $2 \times 10^{-10}$ |
| *PRSS35* | 6 | 83.51 | -0.15 | $2 \times 10^{-10}$ |
| *GALNT13* | 2 | 153.87 | -0.15 | $2 \times 10^{-10}$ |
| *ENTPD6* | 20 | 25.20 | -0.24 | $1 \times 10^{-18}$ |
| *CEACAM8* | 19 | 42.58 | -0.16 | $2 \times 10^{-10}$ |
| *FLT3* | 13 | 28.00 | 0.19 | $1 \times 10^{-10}$ |
| *GLYAT* | 11 | 58.64 | -0.16 | $2 \times 10^{-10}$ |
| *CCDC90B* | 11 | 83.26 | -0.16 | $2 \times 10^{-10}$ |
| *FABP12* | 8 | 81.52 | -0.16 | $2 \times 10^{-10}$ |
| *KCNF1* | 2 | 10.91 | -0.15 | $2 \times 10^{-10}$ |
| *RERG* | 12 | 15.11 | -0.15 | $2 \times 10^{-10}$ |
| *EFNB2* | 13 | 106.49 | -0.16 | $2 \times 10^{-10}$ |
| *DHX9* | 1 | 182.84 | -0.15 | $2 \times 10^{-10}$ |
| *LDOC1* | X | 141.11 | -0.15 | $3 \times 10^{-10}$ |
| *CD226* | 18 | 69.83 | -0.15 | $3 \times 10^{-10}$ |
| *TENM3* | 4 | 182.14 | 0.17 | $3 \times 10^{-10}$ |
| *MIOX* | 22 | 50.49 | -0.15 | $3 \times 10^{-10}$ |
| *FIBP* | 11 | 65.88 | -0.15 | $3 \times 10^{-10}$ |
| *RNASE2* | 14 | 20.96 | 0.16 | $3 \times 10^{-10}$ |
| *BCAR3* | 1 | 93.56 | -0.15 | $3 \times 10^{-10}$ |
| *TCL1B* | 14 | 95.69 | -0.15 | $4 \times 10^{-10}$ |
| *TOMM20L* | 14 | 58.40 | 0.17 | $4 \times 10^{-10}$ |
| *SMS* | X | 21.94 | -0.15 | $4 \times 10^{-10}$ |
| *PROS1* | 3 | 93.87 | -0.16 | $4 \times 10^{-10}$ |
| *DCP1A* | 3 | 53.28 | -0.15 | $5 \times 10^{-10}$ |
| *HTATIP2* | 11 | 20.36 | -0.15 | $5 \times 10^{-10}$ |
| *BIN1* | 2 | 127.05 | -0.15 | $5 \times 10^{-10}$ |
| *C1RL* | 12 | 7.09 | -0.15 | $5 \times 10^{-10}$ |
| *FZD10* | 12 | 130.16 | -0.16 | $5 \times 10^{-10}$ |
| *ACRV1* | 11 | 125.67 | -0.15 | $5 \times 10^{-10}$ |

**Table S3.** Association of HLA-specific *trans*-scores and SLE *(continued)*

| Gene symbol | Chr | Start position (Mb) | Log OR | P |
|---|---|---|---|---|
| *OCIAD1* | 4 | 48.81 | -0.15 | $6 \times 10^{-10}$ |
| *CDH11* | 16 | 64.94 | -0.15 | $6 \times 10^{-10}$ |
| *PRPF6* | 20 | 63.98 | -0.15 | $6 \times 10^{-10}$ |
| *LYG1* | 2 | 99.28 | -0.16 | $6 \times 10^{-10}$ |
| *CDCP1* | 3 | 45.08 | 0.19 | $7 \times 10^{-10}$ |
| *FGFR1* | 8 | 38.40 | -0.16 | $7 \times 10^{-10}$ |
| *HCN1* | 5 | 45.25 | -0.15 | $8 \times 10^{-10}$ |
| *TPD52L1* | 6 | 125.12 | -0.15 | $9 \times 10^{-10}$ |
| *CFAP45* | 1 | 159.87 | -0.15 | $9 \times 10^{-10}$ |
| *CPN2* | 3 | 194.34 | 0.17 | $1 \times 10^{-9}$ |
| *HIF1AN* | 10 | 100.53 | -0.15 | $1 \times 10^{-9}$ |
| *ADAM23* | 2 | 206.44 | -0.15 | $1 \times 10^{-9}$ |
| *FLT3LG* | 19 | 49.47 | 0.19 | $1 \times 10^{-9}$ |
| *FAM174A* | 5 | 100.54 | -0.15 | $1 \times 10^{-9}$ |
| *PSTPIP1* | 15 | 76.99 | -0.15 | $1 \times 10^{-9}$ |
| *SETD2* | 3 | 47.02 | -0.15 | $1 \times 10^{-9}$ |
| *DUSP16* | 12 | 12.47 | -0.15 | $1 \times 10^{-9}$ |
| *PACSIN3* | 11 | 47.18 | -0.15 | $1 \times 10^{-9}$ |
| *PLD5* | 1 | 242.08 | 0.19 | $1 \times 10^{-9}$ |
| *NDST1* | 5 | 150.49 | -0.15 | $1 \times 10^{-9}$ |
| *EFNB3* | 17 | 7.71 | -0.15 | $2 \times 10^{-9}$ |
| *TNFRSF9* | 1 | 7.92 | -0.20 | $2 \times 10^{-9}$ |
| *RNASE3* | 14 | 20.89 | -0.21 | $4 \times 10^{-10}$ |
| *STAP1* | 4 | 67.56 | -0.14 | $2 \times 10^{-9}$ |
| *CSRP2* | 12 | 76.86 | -0.15 | $2 \times 10^{-9}$ |
| *VOPP1* | 7 | 55.44 | -0.15 | $2 \times 10^{-9}$ |
| *ACADS* | 12 | 120.73 | -0.14 | $2 \times 10^{-9}$ |
| *S100A10* | 1 | 151.98 | -0.15 | $2 \times 10^{-9}$ |
| *TREM1* | 6 | 41.27 | -0.15 | $2 \times 10^{-9}$ |
| *NR4A1* | 12 | 52.02 | 0.15 | $2 \times 10^{-9}$ |
| *PNLIPRP1* | 10 | 116.59 | -0.14 | $2 \times 10^{-9}$ |
| *ADIPOQ* | 3 | 186.84 | -0.15 | $2 \times 10^{-9}$ |
| *CD8B* | 2 | 86.82 | -0.14 | $2 \times 10^{-9}$ |
| *TNFRSF13B* | 17 | 16.93 | -0.15 | $2 \times 10^{-9}$ |
| *KCNIP1* | 5 | 170.35 | -0.14 | $2 \times 10^{-9}$ |
| *CRP* | 1 | 159.71 | -0.14 | $2 \times 10^{-9}$ |
| *THTPA* | 14 | 23.56 | -0.14 | $2 \times 10^{-9}$ |
| *SH2D1B* | 1 | 162.40 | -0.14 | $3 \times 10^{-9}$ |
| *WNT10A* | 2 | 218.88 | -0.14 | $3 \times 10^{-9}$ |
| *FGF8* | 10 | 101.77 | -0.14 | $3 \times 10^{-9}$ |
| *FN1* | 2 | 215.36 | -0.14 | $3 \times 10^{-9}$ |
| *CXCL17* | 19 | 42.43 | -0.15 | $3 \times 10^{-9}$ |
| *CFD* | 19 | 0.86 | -0.14 | $3 \times 10^{-9}$ |
| *EIF4EBP3* | 5 | 140.55 | -0.14 | $3 \times 10^{-9}$ |
| *ALK* | 2 | 29.19 | 0.15 | $3 \times 10^{-9}$ |
| *PIGR* | 1 | 206.93 | -0.14 | $4 \times 10^{-9}$ |
| *ISL1* | 5 | 51.38 | -0.14 | $4 \times 10^{-9}$ |
| *SMARCC1* | 3 | 47.59 | -0.19 | $4 \times 10^{-9}$ |
| *SUOX* | 12 | 56.00 | -0.14 | $4 \times 10^{-9}$ |
| *PCSK7* | 11 | 117.20 | -0.14 | $4 \times 10^{-9}$ |
| *IFNA2* | 9 | 21.38 | 0.15 | $4 \times 10^{-9}$ |
| *C1orf115* | 1 | 220.69 | 0.15 | $4 \times 10^{-9}$ |
| *GNPTG* | 16 | 1.35 | -0.14 | $4 \times 10^{-9}$ |
| *TXNIP* | 1 | 145.99 | -0.15 | $4 \times 10^{-9}$ |

**Table S3.** Association of HLA-specific *trans*-scores and SLE *(continued)*

| Gene symbol | Chr | Start position (Mb) | Log OR | P |
|---|---|---|---|---|
| *CD72* | 9 | 35.61 | 0.22 | $4 \times 10^{-18}$ |
| *B3GALT2* | 1 | 193.18 | -0.14 | $4 \times 10^{-9}$ |
| *NBR1* | 17 | 43.17 | -0.14 | $4 \times 10^{-9}$ |
| *C5* | 9 | 120.93 | -0.14 | $5 \times 10^{-9}$ |
| *MECP2* | X | 154.02 | -0.14 | $5 \times 10^{-9}$ |
| *SH3GLB1* | 1 | 86.70 | -0.14 | $5 \times 10^{-9}$ |
| *DUSP4* | 8 | 29.33 | -0.14 | $5 \times 10^{-9}$ |
| *RNF149* | 2 | 101.27 | -0.14 | $5 \times 10^{-9}$ |
| *SUSD1* | 9 | 112.04 | -0.14 | $5 \times 10^{-9}$ |
| *PKDCC* | 2 | 42.05 | -0.14 | $6 \times 10^{-9}$ |
| *CETN2* | X | 152.83 | -0.16 | $6 \times 10^{-9}$ |
| *JTB* | 1 | 153.97 | -0.14 | $6 \times 10^{-9}$ |
| *DEFB129* | 20 | 0.23 | -0.14 | $6 \times 10^{-9}$ |
| *CDC34* | 19 | 0.53 | -0.14 | $6 \times 10^{-9}$ |
| *EDDM3B* | 14 | 20.77 | -0.14 | $6 \times 10^{-9}$ |
| *PPIG* | 2 | 169.58 | 0.16 | $6 \times 10^{-9}$ |
| *SDC3* | 1 | 30.87 | -0.14 | $6 \times 10^{-9}$ |
| *ADAM15* | 1 | 155.05 | -0.14 | $7 \times 10^{-9}$ |
| *ILF3* | 19 | 10.65 | -0.15 | $7 \times 10^{-9}$ |
| *HBQ1* | 16 | 0.18 | -0.14 | $7 \times 10^{-9}$ |
| *TRH* | 3 | 129.97 | -0.14 | $8 \times 10^{-9}$ |
| *PDK2* | 17 | 50.09 | -0.14 | $8 \times 10^{-9}$ |
| *LCN2* | 9 | 128.15 | -0.14 | $8 \times 10^{-9}$ |
| *TMEM9* | 1 | 201.13 | -0.15 | $8 \times 10^{-9}$ |
| *SERPIND1* | 22 | 20.77 | -0.14 | $8 \times 10^{-9}$ |
| *CRH* | 8 | 66.18 | -0.14 | $9 \times 10^{-9}$ |
| *RNPEP* | 1 | 201.98 | -0.14 | $9 \times 10^{-9}$ |
| *KPNA2* | 17 | 68.04 | -0.16 | $9 \times 10^{-9}$ |
| *GFRA2* | 8 | 21.69 | -0.14 | $9 \times 10^{-9}$ |
| *JPH1* | 8 | 74.23 | -0.14 | $1 \times 10^{-8}$ |
| *BCAM* | 19 | 44.81 | -0.14 | $1 \times 10^{-8}$ |
| *NRIP3* | 11 | 8.97 | -0.14 | $1 \times 10^{-8}$ |
| *SPARCL1* | 4 | 87.47 | 0.15 | $1 \times 10^{-8}$ |
| *SPHK2* | 19 | 48.62 | -0.15 | $1 \times 10^{-8}$ |
| *REPIN1* | 7 | 150.37 | -0.14 | $1 \times 10^{-8}$ |
| *RIPPLY1* | X | 106.90 | -0.14 | $1 \times 10^{-8}$ |
| *NLRP1* | 17 | 5.50 | -0.14 | $1 \times 10^{-8}$ |
| *IL36A* | 2 | 113.01 | -0.14 | $1 \times 10^{-8}$ |
| *DCBLD1* | 6 | 117.45 | -0.14 | $1 \times 10^{-8}$ |
| *ARFGAP1* | 20 | 63.27 | -0.14 | $1 \times 10^{-8}$ |
| *PLXND1* | 3 | 129.56 | -0.14 | $1 \times 10^{-8}$ |
| *MMP10* | 11 | 102.77 | -0.25 | $3 \times 10^{-16}$ |
| *PSPN* | 19 | 6.38 | -0.14 | $1 \times 10^{-8}$ |
| *ABL2* | 1 | 179.10 | -0.14 | $2 \times 10^{-8}$ |
| *CD1D* | 1 | 158.18 | -0.14 | $2 \times 10^{-8}$ |
| *PON1* | 7 | 95.30 | -0.13 | $2 \times 10^{-8}$ |
| *GAL3ST1* | 22 | 30.55 | -0.14 | $2 \times 10^{-8}$ |
| *UBTD2* | 5 | 172.21 | -0.13 | $2 \times 10^{-8}$ |
| *SELPLG* | 12 | 108.62 | -0.14 | $2 \times 10^{-8}$ |
| *VTI1B* | 14 | 67.65 | -0.13 | $2 \times 10^{-8}$ |
| *GJA1* | 6 | 121.44 | 0.14 | $2 \times 10^{-8}$ |
| *FGF3* | 11 | 69.81 | -0.14 | $2 \times 10^{-8}$ |
| *PKD2* | 4 | 88.01 | -0.13 | $3 \times 10^{-8}$ |
| *RABIF* | 1 | 202.88 | -0.13 | $3 \times 10^{-8}$ |

**Table S3.** Association of HLA-specific *trans*-scores and SLE *(continued)*

| Gene symbol | Chr | Start position (Mb) | Log OR | P |
|---|---|---|---|---|
| *STK17B* | 2 | 196.13 | -0.14 | $3 \times 10^{-8}$ |
| *HMG20B* | 19 | 3.57 | 0.14 | $3 \times 10^{-8}$ |
| *GCSH* | 16 | 81.08 | -0.13 | $3 \times 10^{-8}$ |
| *F3* | 1 | 94.53 | 0.14 | $3 \times 10^{-8}$ |
| *KCNE5* | X | 109.62 | -0.14 | $3 \times 10^{-8}$ |
| *FAM162B* | 6 | 116.75 | -0.13 | $3 \times 10^{-8}$ |
| *CLMP* | 11 | 123.07 | -0.18 | $3 \times 10^{-8}$ |
| *PCSK1N* | X | 48.83 | -0.13 | $3 \times 10^{-8}$ |
| *LRP4* | 11 | 46.86 | -0.14 | $3 \times 10^{-8}$ |
| *SMURF2* | 17 | 64.54 | -0.13 | $3 \times 10^{-8}$ |
| *CLEC11A* | 19 | 50.72 | -0.14 | $3 \times 10^{-8}$ |
| *ABL1* | 9 | 130.71 | -0.13 | $3 \times 10^{-8}$ |
| *KERA* | 12 | 91.05 | -0.13 | $4 \times 10^{-8}$ |
| *PPP3R2* | 9 | 101.59 | -0.13 | $4 \times 10^{-8}$ |
| *FATE1* | X | 151.72 | -0.13 | $4 \times 10^{-8}$ |
| *CCNH* | 5 | 87.32 | -0.13 | $4 \times 10^{-8}$ |
| *HNRNPH1* | 5 | 179.61 | -0.14 | $4 \times 10^{-8}$ |
| *CHFR* | 12 | 132.82 | -0.13 | $4 \times 10^{-8}$ |
| *BRSK2* | 11 | 1.39 | 0.14 | $4 \times 10^{-8}$ |
| *CASP8* | 2 | 201.23 | 0.14 | $4 \times 10^{-8}$ |
| *MSTN* | 2 | 190.06 | -0.16 | $7 \times 10^{-10}$ |
| *LDLRAD4* | 18 | 13.22 | -0.13 | $4 \times 10^{-8}$ |
| *IL1RAPL2* | X | 104.57 | -0.14 | $4 \times 10^{-8}$ |
| *SOCS7* | 17 | 38.35 | -0.13 | $4 \times 10^{-8}$ |
| *CLEC4C* | 12 | 7.73 | -0.13 | $4 \times 10^{-8}$ |
| *TFRC* | 3 | 196.01 | -0.13 | $4 \times 10^{-8}$ |
| *CCL21* | 9 | 34.71 | -0.14 | $5 \times 10^{-8}$ |
| *NDUFB4* | 3 | 120.60 | -0.13 | $5 \times 10^{-8}$ |
| *EXOSC1* | 10 | 97.44 | -0.13 | $5 \times 10^{-8}$ |
| *TNFSF10* | 3 | 172.51 | -0.13 | $5 \times 10^{-8}$ |
| *LYVE1* | 11 | 10.56 | -0.13 | $5 \times 10^{-8}$ |
| *KIF16B* | 20 | 16.27 | -0.13 | $5 \times 10^{-8}$ |
| *CCL2* | 17 | 34.26 | -0.14 | $5 \times 10^{-8}$ |
| *TNFRSF1B* | 1 | 12.17 | -0.19 | $5 \times 10^{-8}$ |
| *HGS* | 17 | 81.68 | -0.13 | $5 \times 10^{-8}$ |
| *TNFRSF4* | 1 | 1.21 | -0.13 | $5 \times 10^{-8}$ |
| *CEACAM19* | 19 | 44.66 | -0.13 | $5 \times 10^{-8}$ |
| *PDXK* | 21 | 43.72 | -0.13 | $5 \times 10^{-8}$ |
| *POR* | 7 | 75.90 | -0.13 | $6 \times 10^{-8}$ |
| *TNFSF18* | 1 | 173.04 | -0.13 | $6 \times 10^{-8}$ |
| *FLT1* | 13 | 28.30 | -0.13 | $6 \times 10^{-8}$ |
| *NNAT* | 20 | 37.52 | -0.13 | $6 \times 10^{-8}$ |
| *NDC80* | 18 | 2.57 | -0.13 | $6 \times 10^{-8}$ |
| *APLN* | X | 129.65 | -0.13 | $6 \times 10^{-8}$ |
| *MRAS* | 3 | 138.35 | 0.14 | $7 \times 10^{-8}$ |
| *PDZD7* | 10 | 101.01 | -0.13 | $7 \times 10^{-8}$ |
| *SCGB3A1* | 5 | 180.59 | 0.13 | $6 \times 10^{-8}$ |
| *TST* | 22 | 37.01 | 0.13 | $8 \times 10^{-8}$ |
| *MICALL2* | 7 | 1.43 | -0.13 | $8 \times 10^{-8}$ |
| *FOXG1* | 14 | 28.77 | -0.13 | $8 \times 10^{-8}$ |
| *SEMA3B* | 3 | 50.27 | -0.13 | $8 \times 10^{-8}$ |
| *PROK1* | 1 | 110.45 | -0.13 | $9 \times 10^{-8}$ |
| *LRRC52* | 1 | 165.54 | -0.13 | $9 \times 10^{-8}$ |
| *COL10A1* | 6 | 116.12 | -0.13 | $9 \times 10^{-8}$ |

**Table S3.** Association of HLA-specific *trans*-scores and SLE *(continued)*

| Gene symbol | Chr | Start position (Mb) | Log OR | P |
|---|---|---|---|---|
| *SAMHD1* | 20 | 36.89 | 0.13 | $9 \times 10^{-8}$ |
| *ENPP6* | 4 | 184.09 | -0.13 | $1 \times 10^{-7}$ |
| *TMEM52B* | 12 | 10.17 | -0.14 | $1 \times 10^{-7}$ |
| *KLRC3* | 12 | 10.41 | -0.13 | $1 \times 10^{-7}$ |
| *C1orf185* | 1 | 51.10 | -0.13 | $1 \times 10^{-7}$ |
| *ANXA13* | 8 | 123.68 | -0.13 | $1 \times 10^{-7}$ |
| *CXCL8* | 4 | 73.74 | 0.13 | $1 \times 10^{-7}$ |
| *NAT1* | 8 | 18.17 | -0.13 | $1 \times 10^{-7}$ |
| *CLEC6A* | 12 | 8.46 | -0.13 | $1 \times 10^{-7}$ |
| *SULT1C2* | 2 | 108.29 | -0.13 | $1 \times 10^{-7}$ |
| *ADAM17* | 2 | 9.49 | -0.13 | $1 \times 10^{-7}$ |
| *IFNA10* | 9 | 21.21 | -0.13 | $1 \times 10^{-7}$ |
| *SPR* | 2 | 72.89 | -0.13 | $1 \times 10^{-7}$ |
| *UBB* | 17 | 16.38 | -0.13 | $1 \times 10^{-7}$ |
| *DEFB119* | 20 | 31.38 | -0.13 | $1 \times 10^{-7}$ |
| *GUSB* | 7 | 65.96 | -0.13 | $2 \times 10^{-7}$ |
| *WFDC10A* | 20 | 45.63 | -0.13 | $2 \times 10^{-7}$ |
| *CDH6* | 5 | 31.19 | -0.13 | $2 \times 10^{-7}$ |
| *IST1* | 16 | 71.85 | -0.13 | $2 \times 10^{-7}$ |
| *NCF1* | 7 | 74.77 | -0.12 | $2 \times 10^{-7}$ |
| *CCL22* | 16 | 57.36 | 0.13 | $2 \times 10^{-7}$ |
| *CD93* | 20 | 23.08 | 0.14 | $1 \times 10^{-7}$ |
| *SMIM9* | X | 154.82 | -0.12 | $2 \times 10^{-7}$ |
| *C10orf105* | 10 | 71.71 | -0.12 | $2 \times 10^{-7}$ |
| *LGR5* | 12 | 71.44 | 0.13 | $2 \times 10^{-7}$ |
| *BRAF* | 7 | 140.72 | -0.13 | $2 \times 10^{-7}$ |
| *CRABP1* | 15 | 78.34 | -0.12 | $2 \times 10^{-7}$ |
| *AKR1D1* | 7 | 138.00 | -0.12 | $2 \times 10^{-7}$ |
| *NEGR1* | 1 | 71.40 | 0.14 | $2 \times 10^{-7}$ |
| *AGR2* | 7 | 16.79 | -0.25 | $9 \times 10^{-12}$ |
| *PARP16* | 15 | 65.23 | -0.13 | $2 \times 10^{-7}$ |
| *BMP2* | 20 | 6.77 | -0.13 | $2 \times 10^{-7}$ |
| *BPIFA1* | 20 | 33.24 | -0.12 | $2 \times 10^{-7}$ |
| *IFNA6* | 9 | 21.35 | -0.12 | $2 \times 10^{-7}$ |
| *COLEC12* | 18 | 0.32 | -0.13 | $3 \times 10^{-7}$ |
| *MSRB2* | 10 | 23.10 | -0.12 | $3 \times 10^{-7}$ |
| *PLXNB2* | 22 | 50.27 | -0.12 | $3 \times 10^{-7}$ |
| *GAD1* | 2 | 170.81 | -0.12 | $3 \times 10^{-7}$ |
| *NID1* | 1 | 235.98 | 0.13 | $3 \times 10^{-7}$ |
| *QPRT* | 16 | 29.66 | -0.12 | $3 \times 10^{-7}$ |
| *DVL2* | 17 | 7.23 | -0.12 | $3 \times 10^{-7}$ |
| *EYS* | 6 | 63.72 | -0.12 | $3 \times 10^{-7}$ |
| *CNTFR* | 9 | 34.55 | -0.12 | $3 \times 10^{-7}$ |
| *SAT1* | X | 23.78 | -0.13 | $4 \times 10^{-7}$ |
| *HMGA2* | 12 | 65.82 | -0.12 | $4 \times 10^{-7}$ |
| *SCGB1A1* | 11 | 62.41 | -0.12 | $4 \times 10^{-7}$ |
| *P2RX6* | 22 | 21.01 | -0.12 | $4 \times 10^{-7}$ |
| *FOXL2* | 3 | 138.94 | -0.12 | $4 \times 10^{-7}$ |
| *GAS6* | 13 | 113.82 | -0.18 | $3 \times 10^{-11}$ |
| *CCL26* | 7 | 75.77 | -0.12 | $4 \times 10^{-7}$ |
| *CPN1* | 10 | 100.04 | -0.13 | $4 \times 10^{-7}$ |
| *PNOC* | 8 | 28.32 | -0.12 | $4 \times 10^{-7}$ |
| *RRAS2* | 11 | 14.28 | -0.12 | $4 \times 10^{-7}$ |
| *SMYD2* | 1 | 214.28 | -0.13 | $4 \times 10^{-7}$ |

**Table S3.** Association of HLA-specific *trans*-scores and SLE *(continued)*

| Gene symbol | Chr | Start position (Mb) | Log OR | P |
|---|---|---|---|---|
| *RNF128* | X | 106.69 | -0.12 | $5 \times 10^{-7}$ |
| *ADAM30* | 1 | 119.89 | -0.12 | $5 \times 10^{-7}$ |
| *IGF2R* | 6 | 159.97 | -0.12 | $5 \times 10^{-7}$ |
| *KRTDAP* | 19 | 35.49 | -0.12 | $5 \times 10^{-7}$ |
| *AKAP7* | 6 | 131.14 | -0.12 | $5 \times 10^{-7}$ |
| *RND1* | 12 | 48.86 | -0.12 | $5 \times 10^{-7}$ |
| *TIMP2* | 17 | 78.85 | -0.12 | $6 \times 10^{-7}$ |
| *PDCD6* | 5 | 0.27 | -0.13 | $6 \times 10^{-7}$ |
| *NFKBID* | 19 | 35.89 | -0.12 | $6 \times 10^{-7}$ |
| *FLT4* | 5 | 180.60 | -0.12 | $6 \times 10^{-7}$ |
| *RBM24* | 6 | 17.28 | -0.12 | $6 \times 10^{-7}$ |
| *FCER2* | 19 | 7.69 | 0.14 | $9 \times 10^{-8}$ |
| *MATN4* | 20 | 45.29 | -0.12 | $6 \times 10^{-7}$ |
| *XG* | X | 2.75 | -0.16 | $6 \times 10^{-7}$ |
| *AGR3* | 7 | 16.86 | -0.17 | $6 \times 10^{-12}$ |
| *HP* | 16 | 72.05 | -0.12 | $7 \times 10^{-7}$ |
| *COX5B* | 2 | 97.65 | -0.12 | $7 \times 10^{-7}$ |
| *CNDP2* | 18 | 74.50 | -0.12 | $7 \times 10^{-7}$ |
| *SLITRK4* | X | 143.62 | -0.12 | $7 \times 10^{-7}$ |
| *ADCYAP1* | 18 | 0.90 | -0.13 | $7 \times 10^{-7}$ |
| *ITM2B* | 13 | 48.23 | -0.12 | $7 \times 10^{-7}$ |
| *SYTL4* | X | 100.67 | -0.14 | $8 \times 10^{-7}$ |
| *KLK9* | 19 | 51.00 | -0.12 | $8 \times 10^{-7}$ |
| *RPS4X* | X | 72.26 | -0.13 | $8 \times 10^{-7}$ |
| *BDP1* | 5 | 71.46 | -0.12 | $8 \times 10^{-7}$ |
| *PSMA3* | 14 | 58.24 | -0.12 | $9 \times 10^{-7}$ |
| *TYRO3* | 15 | 41.56 | -0.12 | $9 \times 10^{-7}$ |
| *NPTX2* | 7 | 98.62 | -0.12 | $9 \times 10^{-7}$ |
| *C1QTNF5* | 11 | 119.34 | 0.12 | $9 \times 10^{-7}$ |
| *CPVL* | 7 | 29.00 | -0.12 | $1 \times 10^{-6}$ |
| *PRC1* | 15 | 90.97 | -0.14 | $1 \times 10^{-8}$ |
| *KLRB1* | 12 | 9.59 | -0.14 | $3 \times 10^{-9}$ |
| *CCL19* | 9 | 34.69 | 0.16 | $6 \times 10^{-10}$ |
| *GFRA1* | 10 | 116.06 | -0.12 | $2 \times 10^{-7}$ |
| *CEBPB* | 20 | 50.19 | -0.14 | $1 \times 10^{-8}$ |
| *CLEC7A* | 12 | 10.12 | 0.23 | $4 \times 10^{-16}$ |
| *CD164L2* | 1 | 27.38 | 0.35 | $4 \times 10^{-40}$ |
| *SLAMF8* | 1 | 159.83 | -0.23 | $8 \times 10^{-12}$ |
| *LMOD1* | 1 | 201.90 | 0.26 | $4 \times 10^{-19}$ |
| *PCBD1* | 10 | 70.88 | -0.13 | $4 \times 10^{-7}$ |
| *FAM171B* | 2 | 186.69 | 0.16 | $4 \times 10^{-10}$ |
| *ITIH4* | 3 | 52.81 | -0.27 | $6 \times 10^{-25}$ |
| *DEFB116* | 20 | 31.30 | -0.19 | $4 \times 10^{-15}$ |
| *TGFBI* | 5 | 136.03 | -0.23 | $1 \times 10^{-13}$ |
| *GZMA* | 5 | 55.10 | -0.14 | $5 \times 10^{-8}$ |
| *LECT2* | 5 | 135.92 | -0.19 | $1 \times 10^{-8}$ |
| *CXCL10* | 4 | 76.02 | 0.16 | $2 \times 10^{-10}$ |
| *CPE* | 4 | 165.36 | 0.17 | $3 \times 10^{-11}$ |
| *CD5* | 11 | 61.10 | 0.16 | $2 \times 10^{-8}$ |
| *QPCT* | 2 | 37.34 | -0.26 | $2 \times 10^{-11}$ |
| *FOLR3* | 11 | 72.11 | -0.21 | $1 \times 10^{-12}$ |
| *IL15RA* | 10 | 5.94 | -0.25 | $4 \times 10^{-11}$ |
| *CA4* | 17 | 60.15 | -0.22 | $2 \times 10^{-12}$ |
| *CRIM1* | 2 | 36.36 | 0.18 | $1 \times 10^{-9}$ |

**Table S3.** Association of HLA-specific *trans*-scores and SLE *(continued)*

| Gene symbol | Chr | Start position (Mb) | Log OR | P |
|---|---|---|---|---|
| *CRELD1* | 3 | 9.93 | -0.16 | $7 \times 10^{-10}$ |
| *CGREF1* | 2 | 27.10 | -0.20 | $6 \times 10^{-15}$ |
| *HSP90B1* | 12 | 103.93 | -0.24 | $5 \times 10^{-22}$ |
| *ANGPTL1* | 1 | 178.85 | -0.14 | $3 \times 10^{-8}$ |
| *RAB39B* | X | 155.26 | 0.19 | $3 \times 10^{-15}$ |
| *IL17C* | 16 | 88.64 | -0.22 | $6 \times 10^{-17}$ |
| *CCL24* | 7 | 75.81 | -0.13 | $1 \times 10^{-7}$ |
| *CPB2* | 13 | 46.05 | -0.20 | $2 \times 10^{-9}$ |
| *ARHGAP1* | 11 | 46.68 | 0.23 | $1 \times 10^{-18}$ |
| *TIGIT* | 3 | 114.28 | 0.29 | $1 \times 10^{-9}$ |
| *IL17A* | 6 | 52.19 | -0.22 | $3 \times 10^{-17}$ |
| *LAIR1* | 19 | 54.35 | 0.14 | $6 \times 10^{-9}$ |
| *S100A12* | 1 | 153.37 | -0.19 | $2 \times 10^{-13}$ |
| *EGLN1* | 1 | 231.36 | -0.23 | $1 \times 10^{-7}$ |
| *RNASE1* | 14 | 20.80 | 0.31 | $3 \times 10^{-32}$ |
| *CXCL13* | 4 | 77.51 | -0.13 | $3 \times 10^{-7}$ |
| *LILRA5* | 19 | 54.31 | -0.15 | $4 \times 10^{-10}$ |
| *SCIN* | 7 | 12.57 | 0.35 | $1 \times 10^{-40}$ |
| *ACRBP* | 12 | 6.64 | 0.34 | $2 \times 10^{-38}$ |
| *IGFBP7* | 4 | 57.03 | 0.33 | $4 \times 10^{-38}$ |
| *REST* | 4 | 56.91 | 0.33 | $1 \times 10^{-37}$ |
| *SIT1* | 9 | 35.65 | -0.33 | $2 \times 10^{-37}$ |
| *ATP6V1D* | 14 | 67.29 | 0.35 | $4 \times 10^{-37}$ |
| *DENR* | 12 | 122.75 | 0.32 | $2 \times 10^{-36}$ |
| *ZNRF4* | 19 | 5.46 | 0.30 | $2 \times 10^{-33}$ |
| *SFTPA2* | 10 | 79.56 | 0.33 | $6 \times 10^{-33}$ |
| *DOCK9* | 13 | 98.79 | 0.30 | $2 \times 10^{-32}$ |
| *MMUT* | 6 | 49.43 | 0.29 | $6 \times 10^{-32}$ |
| *DPP10* | 2 | 114.44 | 0.30 | $2 \times 10^{-31}$ |
| *CD22* | 19 | 35.32 | 0.29 | $4 \times 10^{-31}$ |
| *PLCB1* | 20 | 8.08 | -0.30 | $6 \times 10^{-30}$ |
| *ARNT* | 1 | 150.81 | -0.29 | $2 \times 10^{-29}$ |
| *PCDH12* | 5 | 141.94 | 0.30 | $2 \times 10^{-27}$ |
| *CAPG* | 2 | 85.39 | 0.30 | $5 \times 10^{-27}$ |
| *PPT1* | 1 | 40.07 | 0.29 | $6 \times 10^{-27}$ |
| *IL17F* | 6 | 52.24 | -0.28 | $7 \times 10^{-27}$ |
| *CD33* | 19 | 51.23 | 0.27 | $1 \times 10^{-26}$ |
| *CFH* | 1 | 196.65 | -0.30 | $2 \times 10^{-26}$ |
| *CD2* | 1 | 116.75 | 0.27 | $2 \times 10^{-25}$ |
| *FETUB* | 3 | 186.64 | -0.27 | $2 \times 10^{-25}$ |
| *CD300E* | 17 | 74.61 | -0.33 | $9 \times 10^{-25}$ |
| *FGR* | 1 | 27.61 | -0.27 | $1 \times 10^{-24}$ |
| *ADH1B* | 4 | 99.30 | -0.27 | $2 \times 10^{-24}$ |
| *DPEP2* | 16 | 67.99 | 0.31 | $4 \times 10^{-24}$ |
| *CRNN* | 1 | 152.41 | -0.25 | $3 \times 10^{-23}$ |
| *NPL* | 1 | 182.79 | 0.33 | $3 \times 10^{-23}$ |
| *CD101* | 1 | 117.00 | 0.25 | $4 \times 10^{-23}$ |
| *OPTC* | 1 | 203.49 | -0.26 | $6 \times 10^{-23}$ |
| *MMP3* | 11 | 102.84 | -0.24 | $7 \times 10^{-23}$ |
| *COPB2* | 3 | 139.35 | 0.24 | $3 \times 10^{-22}$ |
| *ANK2* | 4 | 112.82 | 0.26 | $5 \times 10^{-22}$ |
| *PCSK9* | 1 | 55.04 | -0.29 | $5 \times 10^{-22}$ |
| *SYT1* | 12 | 78.86 | 0.25 | $2 \times 10^{-21}$ |
| *CST1* | 20 | 23.75 | -0.23 | $3 \times 10^{-21}$ |

**Table S3.** Association of HLA-specific *trans*-scores and SLE *(continued)*

| Gene symbol | Chr | Start position (Mb) | Log OR | P |
|---|---|---|---|---|
| *GGH* | 8 | 63.01 | -0.32 | $2 \times 10^{-20}$ |
| *CCDC80* | 3 | 112.60 | -0.24 | $3 \times 10^{-20}$ |
| *NPR1* | 1 | 153.68 | 0.22 | $1 \times 10^{-19}$ |
| *AFP* | 4 | 73.43 | 0.22 | $3 \times 10^{-19}$ |
| *TRPV3* | 17 | 3.51 | 0.24 | $5 \times 10^{-19}$ |
| *REG1B* | 2 | 79.09 | -0.23 | $5 \times 10^{-19}$ |
| *SIRT1* | 10 | 67.88 | -0.22 | $8 \times 10^{-19}$ |
| *GSTA3* | 6 | 52.90 | -0.22 | $1 \times 10^{-18}$ |
| *RTN4R* | 22 | 20.24 | -0.22 | $2 \times 10^{-18}$ |
| *RNF4* | 4 | 2.46 | -0.21 | $2 \times 10^{-18}$ |
| *FGA* | 4 | 154.58 | -0.23 | $5 \times 10^{-18}$ |
| *CD200R1* | 3 | 112.92 | 0.21 | $5 \times 10^{-18}$ |
| *IL19* | 1 | 206.77 | -0.21 | $6 \times 10^{-18}$ |
| *VNN1* | 6 | 132.68 | -0.23 | $6 \times 10^{-18}$ |
| *TSPAN7* | X | 38.56 | 0.22 | $9 \times 10^{-18}$ |
| *SFRP1* | 8 | 41.26 | 0.22 | $1 \times 10^{-17}$ |
| *CD80* | 3 | 119.52 | 0.21 | $4 \times 10^{-17}$ |
| *ENAH* | 1 | 225.49 | 0.21 | $9 \times 10^{-17}$ |
| *HAVCR1* | 5 | 157.03 | -0.20 | $2 \times 10^{-16}$ |
| *LSP1* | 11 | 1.85 | -0.24 | $2 \times 10^{-16}$ |
| *CREB3* | 9 | 35.73 | 0.20 | $4 \times 10^{-16}$ |
| *F9* | X | 139.53 | -0.21 | $5 \times 10^{-16}$ |
| *RELB* | 19 | 45.00 | -0.19 | $7 \times 10^{-16}$ |
| *SKAP1* | 17 | 48.13 | -0.23 | $7 \times 10^{-16}$ |
| *LRIG3* | 12 | 58.87 | -0.21 | $1 \times 10^{-15}$ |
| *AIF1L* | 9 | 131.10 | 0.21 | $1 \times 10^{-15}$ |
| *BMPER* | 7 | 33.90 | -0.25 | $2 \times 10^{-15}$ |
| *VCPKMT* | 14 | 50.11 | 0.20 | $3 \times 10^{-15}$ |
| *MYOC* | 1 | 171.64 | -0.26 | $3 \times 10^{-15}$ |
| *DTX2* | 7 | 76.46 | 0.28 | $4 \times 10^{-15}$ |
| *RAP1A* | 1 | 111.54 | 0.19 | $1 \times 10^{-14}$ |
| *SERPING1* | 11 | 57.60 | -0.19 | $1 \times 10^{-14}$ |
| *NAGA* | 22 | 42.06 | -0.20 | $2 \times 10^{-14}$ |
| *FGF23* | 12 | 4.37 | -0.19 | $3 \times 10^{-14}$ |
| *LCAT* | 16 | 67.94 | -0.23 | $4 \times 10^{-14}$ |
| *IGF1R* | 15 | 98.65 | 0.21 | $4 \times 10^{-14}$ |
| *OMG* | 17 | 31.27 | 0.24 | $5 \times 10^{-14}$ |
| *ANG* | 14 | 20.68 | -0.20 | $5 \times 10^{-14}$ |
| *CDH3* | 16 | 68.64 | -0.18 | $6 \times 10^{-14}$ |
| *TIMD4* | 5 | 156.92 | 0.19 | $7 \times 10^{-14}$ |
| *LILRB4* | 19 | 54.64 | 0.19 | $8 \times 10^{-14}$ |
| *NCF2* | 1 | 183.55 | -0.26 | $8 \times 10^{-14}$ |
| *SDC1* | 2 | 20.20 | 0.19 | $8 \times 10^{-14}$ |
| *PRAP1* | 10 | 133.35 | -0.20 | $9 \times 10^{-14}$ |
| *SH3GL3* | 15 | 83.45 | 0.18 | $1 \times 10^{-13}$ |
| *IGSF21* | 1 | 18.11 | -0.23 | $2 \times 10^{-13}$ |
| *ARHGAP5* | 14 | 32.08 | 0.19 | $2 \times 10^{-13}$ |
| *IL20RB* | 3 | 136.95 | -0.22 | $3 \times 10^{-13}$ |
| *PNLIPRP2* | 10 | 116.62 | 0.31 | $3 \times 10^{-13}$ |
| *SERPINA5* | 14 | 94.56 | -0.28 | $4 \times 10^{-13}$ |
| *IL4R* | 16 | 27.31 | 0.19 | $4 \times 10^{-13}$ |
| *OBP2B* | 9 | 133.21 | 0.25 | $5 \times 10^{-13}$ |
| *PCNA* | 20 | 5.11 | 0.18 | $5 \times 10^{-13}$ |
| *B4GALT1* | 9 | 33.10 | 0.17 | $5 \times 10^{-13}$ |

**Table S3.** Association of HLA-specific *trans*-scores and SLE *(continued)*

| Gene symbol | Chr | Start position (Mb) | Log OR | P |
|---|---|---|---|---|
| *CXCL3* | 4 | 74.04 | 0.19 | $6 \times 10^{-13}$ |
| *ATP6AP2* | X | 40.58 | 0.18 | $7 \times 10^{-13}$ |
| *NCS1* | 9 | 130.17 | 0.20 | $8 \times 10^{-13}$ |
| *NGF* | 1 | 115.29 | 0.19 | $9 \times 10^{-13}$ |
| *CCNE1* | 19 | 29.81 | -0.18 | $1 \times 10^{-12}$ |
| *CX3CL1* | 16 | 57.37 | 0.20 | $2 \times 10^{-12}$ |
| *SLC39A14* | 8 | 22.37 | 0.19 | $2 \times 10^{-12}$ |
| *CRTAC1* | 10 | 97.86 | -0.32 | $2 \times 10^{-12}$ |
| *ACVRL1* | 12 | 51.91 | 0.18 | $4 \times 10^{-12}$ |
| *CA8* | 8 | 60.19 | -0.17 | $4 \times 10^{-12}$ |
| *TCL1A* | 14 | 95.71 | 0.17 | $6 \times 10^{-12}$ |
| *SNU13* | 22 | 41.67 | -0.17 | $6 \times 10^{-12}$ |
| *INPP5J* | 22 | 31.12 | -0.17 | $6 \times 10^{-12}$ |
| *CNTN5* | 11 | 99.02 | -0.17 | $7 \times 10^{-12}$ |
| *LY75* | 2 | 159.80 | -0.17 | $7 \times 10^{-12}$ |
| *PCDH9* | 13 | 66.30 | 0.20 | $7 \times 10^{-12}$ |
| *C1QL2* | 2 | 119.16 | 0.18 | $7 \times 10^{-12}$ |
| *QSOX1* | 1 | 180.15 | -0.18 | $1 \times 10^{-11}$ |
| *NMNAT1* | 1 | 9.94 | -0.26 | $2 \times 10^{-11}$ |
| *FAM3C* | 7 | 121.35 | -0.18 | $2 \times 10^{-11}$ |
| *A1BG* | 19 | 58.35 | -0.16 | $2 \times 10^{-11}$ |
| *SERPINH1* | 11 | 75.56 | 0.21 | $3 \times 10^{-11}$ |
| *EPCAM* | 2 | 47.35 | -0.16 | $4 \times 10^{-11}$ |
| *VIT* | 2 | 36.70 | -0.17 | $5 \times 10^{-11}$ |
| *PLXDC1* | 17 | 39.06 | 0.20 | $5 \times 10^{-11}$ |
| *SAA4* | 11 | 18.23 | -0.17 | $5 \times 10^{-11}$ |
| *SCG3* | 15 | 51.68 | -0.16 | $6 \times 10^{-11}$ |
| *IKZF2* | 2 | 213.00 | -0.17 | $7 \times 10^{-11}$ |
| *CXCL1* | 4 | 73.87 | 0.16 | $8 \times 10^{-11}$ |
| *APBB1IP* | 10 | 26.44 | -0.23 | $9 \times 10^{-11}$ |
| *ITGA6* | 2 | 172.43 | 0.18 | $1 \times 10^{-10}$ |
| *SORD* | 15 | 45.02 | -0.16 | $1 \times 10^{-10}$ |
| *SMPD3* | 16 | 68.36 | -0.17 | $1 \times 10^{-10}$ |
| *SLC4A1* | 17 | 44.25 | 0.16 | $2 \times 10^{-10}$ |
| *BAMBI* | 10 | 28.68 | -0.23 | $2 \times 10^{-10}$ |
| *ACTN2* | 1 | 236.66 | 0.16 | $3 \times 10^{-10}$ |
| *CENPJ* | 13 | 24.88 | 0.17 | $4 \times 10^{-10}$ |
| *TRIM5* | 11 | 5.66 | -0.28 | $4 \times 10^{-10}$ |
| *LAMB1* | 7 | 107.92 | -0.15 | $4 \times 10^{-10}$ |
| *ADRA2A* | 10 | 111.08 | 0.15 | $5 \times 10^{-10}$ |
| *CHIT1* | 1 | 203.21 | 0.18 | $5 \times 10^{-10}$ |
| *ZP3* | 7 | 76.40 | 0.18 | $5 \times 10^{-10}$ |
| *OGA* | 10 | 101.78 | -0.25 | $6 \times 10^{-10}$ |
| *F7* | 13 | 113.11 | -0.21 | $6 \times 10^{-10}$ |
| *CDA* | 1 | 20.59 | -0.24 | $6 \times 10^{-10}$ |
| *IGFBP1* | 7 | 45.89 | 0.19 | $7 \times 10^{-10}$ |
| *TDP1* | 14 | 89.95 | -0.18 | $7 \times 10^{-10}$ |
| *CCL3* | 17 | 36.09 | -0.15 | $7 \times 10^{-10}$ |
| *ASPSCR1* | 17 | 81.98 | 0.18 | $7 \times 10^{-10}$ |
| *CAPS* | 19 | 5.91 | -0.16 | $8 \times 10^{-10}$ |
| *SSNA1* | 9 | 137.19 | 0.17 | $8 \times 10^{-10}$ |
| *CTSS* | 1 | 150.73 | -0.15 | $1 \times 10^{-9}$ |
| *LDLR* | 19 | 11.09 | -0.18 | $1 \times 10^{-9}$ |
| *CCN3* | 8 | 119.42 | -0.15 | $1 \times 10^{-9}$ |

**Table S3.** Association of HLA-specific *trans*-scores and SLE *(continued)*

| Gene symbol | Chr | Start position (Mb) | Log OR | P |
|---|---|---|---|---|
| *SERPINF2* | 17 | 1.74 | -0.20 | $1 \times 10^{-9}$ |
| *CLEC5A* | 7 | 141.93 | 0.18 | $2 \times 10^{-9}$ |
| *LSM8* | 7 | 118.18 | 0.23 | $2 \times 10^{-9}$ |
| *NBN* | 8 | 89.92 | -0.26 | $2 \times 10^{-9}$ |
| *TSHB* | 1 | 115.03 | -0.15 | $2 \times 10^{-9}$ |
| *LYPLA2* | 1 | 23.79 | 0.15 | $3 \times 10^{-9}$ |
| *PDGFRA* | 4 | 54.23 | -0.17 | $3 \times 10^{-9}$ |
| *PFDN4* | 20 | 54.21 | 0.15 | $3 \times 10^{-9}$ |
| *SELE* | 1 | 169.72 | -0.15 | $3 \times 10^{-9}$ |
| *PGR* | 11 | 101.03 | -0.16 | $3 \times 10^{-9}$ |
| *PXN* | 12 | 120.21 | -0.18 | $5 \times 10^{-9}$ |
| *ARHGEF5* | 7 | 144.36 | 0.15 | $5 \times 10^{-9}$ |
| *RAB6A* | 11 | 73.68 | -0.28 | $6 \times 10^{-9}$ |
| *CPM* | 12 | 68.84 | -0.19 | $6 \times 10^{-9}$ |
| *PLXDC2* | 10 | 19.82 | 0.14 | $6 \times 10^{-9}$ |
| *SIRPA* | 20 | 1.89 | 0.16 | $6 \times 10^{-9}$ |
| *GALNT3* | 2 | 165.75 | -0.14 | $7 \times 10^{-9}$ |
| *TOP2B* | 3 | 25.60 | -0.21 | $8 \times 10^{-9}$ |
| *NAGK* | 2 | 71.06 | 0.18 | $1 \times 10^{-8}$ |
| *OGT* | X | 71.53 | 0.15 | $1 \times 10^{-8}$ |
| *PRRT3* | 3 | 9.95 | 0.14 | $1 \times 10^{-8}$ |
| *CD34* | 1 | 207.88 | 0.15 | $1 \times 10^{-8}$ |
| *KLKB1* | 4 | 186.21 | -0.16 | $1 \times 10^{-8}$ |
| *CLSPN* | 1 | 35.72 | -0.20 | $1 \times 10^{-8}$ |
| *IL6* | 7 | 22.73 | -0.14 | $1 \times 10^{-8}$ |
| *PON2* | 7 | 95.40 | -0.14 | $2 \times 10^{-8}$ |
| *CCL28* | 5 | 43.38 | 0.14 | $2 \times 10^{-8}$ |
| *BCAN* | 1 | 156.64 | 0.20 | $2 \times 10^{-8}$ |
| *FNDC1* | 6 | 159.17 | 0.15 | $2 \times 10^{-8}$ |
| *CBLIF* | 11 | 59.83 | 0.20 | $2 \times 10^{-8}$ |
| *PROC* | 2 | 127.42 | -0.16 | $2 \times 10^{-8}$ |
| *FABP9* | 8 | 81.46 | -0.14 | $3 \times 10^{-8}$ |
| *CFI* | 4 | 109.73 | -0.17 | $3 \times 10^{-8}$ |
| *MERTK* | 2 | 111.90 | 0.13 | $4 \times 10^{-8}$ |
| *IL12RB1* | 19 | 18.06 | -0.14 | $4 \times 10^{-8}$ |
| *HYOU1* | 11 | 119.04 | -0.14 | $5 \times 10^{-8}$ |
| *RPA2* | 1 | 27.89 | -0.19 | $5 \times 10^{-8}$ |
| *ITGB2* | 21 | 44.89 | -0.15 | $5 \times 10^{-8}$ |
| *NPC2* | 14 | 74.48 | 0.13 | $5 \times 10^{-8}$ |
| *S100A11* | 1 | 152.03 | -0.20 | $5 \times 10^{-8}$ |
| *ALPP* | 2 | 232.38 | 0.16 | $6 \times 10^{-8}$ |
| *CLASP1* | 2 | 121.34 | 0.13 | $7 \times 10^{-8}$ |
| *RAB44* | 6 | 36.70 | -0.23 | $8 \times 10^{-8}$ |
| *ARHGAP30* | 1 | 161.05 | 0.14 | $9 \times 10^{-8}$ |
| *GPA33* | 1 | 167.05 | -0.18 | $1 \times 10^{-7}$ |
| *PILRB* | 7 | 100.35 | -0.17 | $1 \times 10^{-7}$ |
| *CTRC* | 1 | 15.44 | 0.17 | $1 \times 10^{-7}$ |
| *KDM3A* | 2 | 86.44 | -0.13 | $1 \times 10^{-7}$ |
| *HS3ST3B1* | 17 | 14.30 | -0.13 | $2 \times 10^{-7}$ |
| *MTHFSD* | 16 | 86.53 | -0.17 | $2 \times 10^{-7}$ |
| *PDP1* | 8 | 93.86 | -0.13 | $3 \times 10^{-7}$ |
| *FOLH1* | 11 | 49.15 | -0.12 | $4 \times 10^{-7}$ |
| *CTSL* | 9 | 87.72 | 0.13 | $4 \times 10^{-7}$ |
| *CSF2* | 5 | 132.07 | -0.19 | $4 \times 10^{-7}$ |

**Table S3.** Association of HLA-specific *trans*-scores and SLE *(continued)*

| Gene symbol | Chr | Start position (Mb) | Log OR | P |
|---|---|---|---|---|
| *MDK* | 11 | 46.38 | 0.14 | $4 \times 10^{-7}$ |
| *SNX2* | 5 | 122.78 | 0.13 | $7 \times 10^{-7}$ |
| *AGXT* | 2 | 240.87 | -0.14 | $7 \times 10^{-7}$ |
| *PPP1R9B* | 17 | 50.13 | -0.24 | $7 \times 10^{-7}$ |
| *IL6R* | 1 | 154.41 | -0.18 | $9 \times 10^{-7}$ |

**Table S4.** Genetic contribution to disease attributable to *trans*-effects on the expression (or protein level) of each putative core gene.

| Gene | $h^2_{cis}$ | $h^2_{trans}$ | Log OR | $r^2$ | $r^2/h^2_{trans}$ | $I$ |
|---|---|---|---|---|---|---|
| ***Trans*-effects on transcript levels** | | | | | | |
| *(RSAD2)* | 0.01 | 0.35 | 0.23 | 0.01 | 0.03 | 1.34 |
| *(IFI44L)* | 0.00 | 0.35 | 0.17 | 0.02 | 0.06 | 0.34 |
| *(HERC5)* | 0.00 | 0.39 | 0.16 | 0.02 | 0.05 | 0.34 |
| *(IFI44)* | 0.00 | 0.37 | 0.16 | 0.02 | 0.05 | 0.32 |
| *(MX1)* | 0.05 | 0.38 | 0.16 | 0.02 | 0.05 | 0.34 |
| *(OAS3)* | 0.01 | 0.37 | 0.15 | 0.02 | 0.05 | 0.31 |
| *(IFIT1)* | 0.02 | 0.41 | 0.15 | 0.01 | 0.02 | 0.75 |
| *(IFIT2)* | 0.01 | 0.29 | 0.15 | 0.01 | 0.02 | 0.68 |
| ***Trans*-effects on protein levels** | | | | | | |
| *CD27* | 0.06 | 0.20 | 0.18 | 0.02 | 0.10 | 0.23 |
| *TNFRSF17* | 0.02 | 0.21 | 0.18 | 0.04 | 0.19 | 0.12 |
| *CD5L* | 0.04 | 0.26 | 0.18 | 0.07 | 0.27 | 0.08 |
| *LGALS2* | . | . | 0.16 | . | . | . |
| *CXCL10* | 0.05 | 0.09 | 0.15 | 0.02 | 0.22 | 0.07 |
| *CRTAM* | 0.10 | 0.17 | 0.14 | 0.03 | 0.17 | 0.09 |
| *CXCL13* | 0.00 | 0.11 | 0.15 | 0.01 | 0.09 | 0.17 |
| *TNFRSF13B* | 0.02 | 0.19 | 0.14 | 0.02 | 0.11 | 0.13 |
| *TNFRSF9* | 0.02 | 0.19 | 0.14 | 0.02 | 0.11 | 0.13 |
| *MX1* | . | . | 0.14 | . | . | . |
| *SLAMF7* | 0.29 | 0.16 | 0.13 | 0.04 | 0.25 | 0.05 |
| *SIGLEC1* | 0.15 | 0.24 | 0.13 | 0.03 | 0.13 | 0.10 |
| *ZG16* | . | . | 0.13 | . | . | . |
| *ALKBH2* | . | . | 0.13 | . | . | . |
| *IL5RA* | 0.22 | 0.27 | 0.12 | 0.07 | 0.26 | 0.04 |
| *FCRL5* | 0.13 | 0.22 | 0.12 | 0.04 | 0.18 | 0.06 |
| *PDCD1* | 0.04 | 0.20 | 0.12 | 0.03 | 0.15 | 0.07 |
| *LAG3* | 0.02 | 0.20 | 0.12 | 0.03 | 0.15 | 0.07 |
| *SHISA5* | 0.02 | 0.13 | 0.12 | 0.01 | 0.08 | 0.13 |
| *CD300E* | 0.12 | 0.19 | 0.12 | 0.03 | 0.16 | 0.07 |

$h^2_{cis}$ – heritability of gene expression (protein levels) explained by *cis*-effects (taken from Ouwens *etal* (2020) for gene expression and from Sun *etal* (2023) for proteins.

$h^2_{trans}$ – heritability of gene expression (protein levels) explained by *trans*-effects (extracted as above).

Log OR - log(odds ratio) for the effect on SLE corresponding to one standard deviation change in GATE score.

$r^2$ – squared correlation between GATE score and measured expression (evaluated in PRECISESADS) or protein level (evaluated in UKB-PPP).

$r^2/h^2_{trans}$ – value of the dilution factor used in equation 1 to adjust the information for discrimination for the fact that the GATE score is a sub-optimal predictor for the *trans*-regulated expression (protein level).

$I$ – information for discrimination (in bits) attributable to *trans*-effects on the expression (protein level) of a gene.

The total information for discrimination attributable to *trans*-effects on the protein levels of the pGATE-detected core genes (i.e. excluding the ISGs) is 1.61 bits.

**Table S5.** Associations between SLE and measured transcript and protein levels for all putative core genes

| Gene | Measured transcript level[a] | | | | Measured protein level[b] | | | |
|---|---|---|---|---|---|---|---|---|
| | Non-cases | Cases | Log OR | P | Non-cases | Cases | Log OR | P |
| ***Trans*-effects on transcript levels** | | | | | | | | |
| *(RSAD2)* | 219 | 149 | 2.06 | $8 \times 10^{-12}$ | . | . | . | . |
| *(IFI44L)* | 219 | 149 | 2.25 | $2 \times 10^{-11}$ | . | . | . | . |
| *(HERC5)* | 219 | 149 | 1.89 | $4 \times 10^{-11}$ | . | . | . | . |
| *(IFI44)* | 219 | 149 | 2.43 | $1 \times 10^{-11}$ | . | . | . | . |
| *(MX1)* | 219 | 149 | 1.74 | $9 \times 10^{-11}$ | . | . | . | . |
| *(OAS3)* | 219 | 149 | 2.16 | $2 \times 10^{-11}$ | . | . | . | . |
| *(IFIT1)* | 219 | 149 | 1.76 | $1 \times 10^{-10}$ | 43102 | 512 | 0.21 | $2 \times 10^{-8}$ |
| *(IFIT2)* | 219 | 149 | 1.83 | $3 \times 10^{-10}$ | . | . | . | . |
| ***Trans*-effects on protein levels** | | | | | | | | |
| *CD27* | 219 | 149 | -0.21 | 0.3 | 50738 | 557 | 0.75 | $8 \times 10^{-90}$ |
| *TNFRSF17* | 219 | 149 | 0.53 | 0.005 | 42932 | 513 | 0.4 | $2 \times 10^{-20}$ |
| *CD5L* | 219 | 149 | -0.07 | 0.7 | 43726 | 520 | 0.25 | $3 \times 10^{-8}$ |
| *LGALS2* | 219 | 149 | 0.01 | 1 | . | . | . | . |
| *CXCL10* | 219 | 149 | 1.61 | $1 \times 10^{-8}$ | 50806 | 556 | 0.68 | $7 \times 10^{-66}$ |
| *CRTAM* | 219 | 149 | -0.16 | 0.5 | 50212 | 538 | 0.39 | $2 \times 10^{-18}$ |
| *CXCL13* | 219 | 149 | 0.76 | $9 \times 10^{-5}$ | 51023 | 552 | 0.61 | $4 \times 10^{-75}$ |
| *TNFRSF13B* | 219 | 149 | -0.35 | 0.05 | 50638 | 555 | 0.34 | $5 \times 10^{-20}$ |
| *TNFRSF9* | 219 | 149 | -0.63 | 0.002 | 50212 | 538 | 0.71 | $4 \times 10^{-78}$ |
| *MX1* | 219 | 149 | 1.74 | $9 \times 10^{-11}$ | . | . | . | . |
| *SLAMF7* | 219 | 149 | 0.01 | 1 | 50530 | 540 | 0.32 | $1 \times 10^{-13}$ |
| *SIGLEC1* | 219 | 149 | 2.13 | $7 \times 10^{-11}$ | 50806 | 556 | 0.82 | $5 \times 10^{-123}$ |
| *ZG16* | 219 | 149 | 0.21 | 0.2 | . | . | . | . |
| *ALKBH2* | 219 | 149 | -0.3 | 0.1 | . | . | . | . |
| *IL5RA* | 219 | 149 | 0.03 | 0.9 | 50530 | 540 | -0.18 | $5 \times 10^{-5}$ |
| *FCRL5* | 219 | 149 | -0.2 | 0.3 | 50604 | 548 | 0.26 | $1 \times 10^{-8}$ |
| *PDCD1* | 219 | 149 | 0.13 | 0.5 | 50921 | 556 | 0.75 | $3 \times 10^{-84}$ |
| *LAG3* | 219 | 149 | 0.46 | 0.02 | 50354 | 552 | 0.69 | $2 \times 10^{-72}$ |
| *SHISA5* | 219 | 149 | 1.07 | $1 \times 10^{-5}$ | 43904 | 522 | 0.59 | $2 \times 10^{-68}$ |
| *CD300E* | 219 | 149 | 1.12 | $3 \times 10^{-5}$ | 50839 | 557 | 0.77 | $2 \times 10^{-76}$ |

Gene symbols in brackets represent a cluster of correlated ISGs.
Dots denote that the circulating protein for the corresponding gene was not measured in UK Biobank.
Log OR: log(odds ratio) for one standard deviation change in the covariate.
P: p-value of association.
[a] Displays tests of association between SLE and measured transcript in the PRECISESADS cohort for all putative core genes
[b] Displays tests of association between SLE and measured circulating proteins in UK Biobank.

**Table S6.** References to the publications reporting validations of putative core genes through perturbation in experimental model or drug effect in humans

| Gene | Experiment | Perturbation | Mouse model of arthritis | Drug effect |
|---|---|---|---|---|
| MX1 | [51] | silencing/overexpression via adenovirus | MRL/lpr mice | [52,53] |
| TNFRSF9 | [54] | knock-out | MRL-Fas(lpr)/4-1BB–/– | . |
| FCRL5 | [55] | upregulation | MD4/Fcrl5 Tg and MD4/ML5/Fcrl5 Tg | . |
| PDCD1 | [56] | spontaneous onset | C57BL/6(B6)-PD-1(-/-) congenic mice | . |
| TNFRSF13B | [57] | heterozygous knock-out | immune complex glomerulonephritis in BAFF-transgenic mice | [58] |
| TNFRSF17 | [59] | knockout | Nba2.APRIL-/- .Yaa, Nba2.TACI-/- .Yaa and double-KO | [58] |

**Table S7.** Table of all protein names and gene name synonyms for core genes identified from pGATE analysis.

| Uniprot ID | Gene names | Protein names |
|---|---|---|
| Q496F6 | *CD300E CD300LE CLM2 CMRF35A5 IREM2* | CMRF35-like molecule 2 (CLM-2) (CD300 antigen-like family member E) (CMRF35-A5) (Immune receptor expressed on myeloid cells 2) (IREM-2) (Polymeric immunoglobulin receptor 2) (PIgR-2) (PIgR2) (Poly-Ig receptor 2) (CD antigen CD300e) |
| O95727 | *CRTAM* | Cytotoxic and regulatory T-cell molecule (Class-I MHC-restricted T-cell-associated molecule) (CD antigen CD355) |
| Q9BZZ2 | *SIGLEC1 SN* | Sialoadhesin (Sialic acid-binding Ig-like lectin 1) (Siglec-1) (CD antigen CD169) |
| O60844 | *ZG16* | Zymogen granule membrane protein 16 (Zymogen granule protein 16) (hZG16) (Secretory lectin ZG16) |
| O43866 | *CD5L API6 UNQ203/PRO229* | CD5 antigen-like (Apoptosis inhibitor expressed by macrophages) (hAIM) (CT-2) (IgM-associated peptide) (SP-alpha) |
| P02778 | *CXCL10 INP10 SCYB10* | C-X-C motif chemokine 10 (10 kDa interferon gamma-induced protein) (Gamma-IP10) (IP-10) (Small-inducible cytokine B10) [Cleaved into: CXCL10(1-73)] |
| Q01344 | *IL5RA IL5R* | Interleukin-5 receptor subunit alpha (IL-5 receptor subunit alpha) (IL-5R subunit alpha) (IL-5R-alpha) (IL-5RA) (CDw125) (CD antigen CD125) |
| O43927 | *CXCL13 BCA1 BLC SCYB13* | C-X-C motif chemokine 13 (Angie) (B cell-attracting chemokine 1) (BCA-1) (B lymphocyte chemoattractant) (CXC chemokine BLC) (Small-inducible cytokine B13) |
| Q96RD9 | *FCRL5 FCRH5 IRTA2 UNQ503/PRO820* | Fc receptor-like protein 5 (FcR-like protein 5) (FcRL5) (BXMAS1) (Fc receptor homolog 5) (FcRH5) (Immune receptor translocation-associated protein 2) (CD antigen CD307e) |
| P05162 | *LGALS2* | Galectin-2 (Gal-2) (Beta-galactoside-binding lectin L-14-II) (HL14) (Lactose-binding lectin 2) (S-Lac lectin 2) |
| P26842 | *CD27 TNFRSF7* | CD27 antigen (CD27L receptor) (T-cell activation antigen CD27) (T14) (Tumor necrosis factor receptor superfamily member 7) (CD antigen CD27) |
| Q6NS38 | *ALKBH2 ABH2* | DNA oxidative demethylase ALKBH2 (EC 1.14.11.33) (Alkylated DNA repair protein alkB homolog 2) (Alpha-ketoglutarate-dependent dioxygenase alkB homolog 2) (Oxy DC1) |
| P18627 | *LAG3 FDC* | Lymphocyte activation gene 3 protein (LAG-3) (CD antigen CD223) [Cleaved into: Secreted lymphocyte activation gene 3 protein (sLAG-3)] |
| Q15116 | *PDCD1 PD1* | Programmed cell death protein 1 (Protein PD-1) (hPD-1) (CD antigen CD279) |
| O14836 | *TNFRSF13B TACI* | Tumor necrosis factor receptor superfamily member 13B (Transmembrane activator and CAML interactor) (CD antigen CD267) |
| P20591 | *MX1* | Interferon-induced GTP-binding protein Mx1 (Interferon-induced protein p78) (IFI-78K) (Interferon-regulated resistance GTP-binding protein MxA) (Myxoma resistance protein 1) (Myxovirus resistance protein 1) [Cleaved into: Interferon-induced GTP-binding protein Mx1, N-terminally processed] |
| Q07011 | *TNFRSF9 CD137 ILA* | Tumor necrosis factor receptor superfamily member 9 (4-1BB ligand receptor) (CDw137) (T-cell antigen 4-1BB homolog) (T-cell antigen ILA) (CD antigen CD137) |
| Q02223 | *TNFRSF17 BCM BCMA* | Tumor necrosis factor receptor superfamily member 17 (B-cell maturation protein) (CD antigen CD269) |
| Q8N114 | *SHISA5 SCOTIN PSEC0133* | Protein shisa-5 (Putative NF-kappa-B-activating protein 120) (Scotin) |
| Q9NQ25 | *SLAMF7 CS1 UNQ576/PRO1138* | SLAM family member 7 (CD2 subset 1) (CD2-like receptor-activating cytotoxic cells) (CRACC) (Membrane protein FOAP-12) (Novel Ly9) (Protein 19A) (CD antigen CD319) |

**Table S8.** Clumps (genomic locations) harboring *trans*-eQTLs contributing to GATE scores for genes strongly associated with SLE in eQTL-GATE analysis

| Chrom | Clump start position (Mb) | Clump end position (Mb) | Target genes | Genes within 200kb | Monogenic within 200kb | Target gene count |
|---|---|---|---|---|---|---|
| 2 | 162.25 | 162.40 | *HERC5, IFI44, IFI44L, IFIT1, MX1, OAS3, RSAD2* | ***FAP***, ***IFIH1***, *GCA, KCNH7* | *IFIH1* | 7 |
| 3 | 113.58 | 113.58 | *IFI44L* | *SIDT1* | | 1 |
| 3 | 159.92 | 159.95 | *IFI44L* | ***IL12A*** | | 1 |
| 5 | 1.28 | 1.29 | *IFIT2* | ***TERT*** | | 1 |
| 7 | 50.22 | 50.33 | *HERC5, IFI44, IFI44L, IFIT1, IFIT2, MX1, OAS3, RSAD2* | ***IKZF1***, ***C7orf72*** | *IKZF1* | 8 |
| 9 | 32.43 | 32.52 | *HERC5, IFI44, IFI44L, IFIT1, IFIT2, MX1, OAS3, RSAD2* | *ACO1, RIGI (DDX58)* | *RIGI (DDX58)* | 8 |
| 9 | 136.36 | 136.46 | *HERC5, IFI44, IFI44L, IFIT1, IFIT2, MX1, OAS3, RSAD2* | *CARD9, DNLZ, ENTR1, GPSM1, INPP5E, ...* | | 8 |
| 10 | 48.82 | 48.91 | *HERC5, IFI44, IFI44L, IFIT1, IFIT2, MX1, OAS3, RSAD2* | ***WDFY4***, ***LRRC18*** | | 8 |
| 15 | 51.23 | 51.46 | *HERC5, IFI44, IFI44L, IFIT1, MX1* | *CYP19A1, DMXL2, GLDN, MIR4713, MIR4713HG* | | 5 |
| 16 | 31.26 | 31.36 | *RSAD2* | ***ITGAM***, ***ITGAX*** | | 1 |
| 17 | 46.71 | 46.78 | *MX1* | *NSF, RPS7P11, WNT3* | | 1 |

For each clump that harbors *trans*-QTLs for a target gene, a locus-specific score for that clump and that gene is computed. The GATE score for each gene is then computed by adding up these locus-specific scores across all clumps. Genes within 200kb from the clump are in bold if they have been previously reported as SLE GWAS hits. Only the first 5 genes are listed for each clump, and three dots denote the presence of more than 5 genes in that location. If rare SNPs within 200kb from the clump were associated with the monogenic Lupus, we report the corresponding gene. A single dot denotes a clump which was not annotated by genes in Ensembl.

**Table S9.** Clumps (genomic locations) harboring *trans*-pQTLs contributing to GATE scores for putative core genes for SLE detected via pQTL-GATE analysis

| Chrom | Clump start position (Mb) | Clump end position (Mb) | Target genes | Genes within 200kb from the clump | Monogenic within 200kb | Target gene count |
|---|---|---|---|---|---|---|
| 1 | 1.21 | 1.25 | *PDCD1* | ***C1QTNF12***, *B3GALT6, SDF4, TNFRSF4* | *ISG15* | 1 |
| 1 | 11.06 | 11.28 | *CD5L* | *ANGPTL7, EXOSC10, MTOR, RPL39P6, SRM, ...* | | 1 |
| 1 | 23.61 | 23.66 | *CD5L* | *MDS2* | | 1 |
| 1 | 24.38 | 25.17 | *CD5L, FCRL5, IL5RA, SLAMF7* | *+* | | 4 |
| 1 | 26.24 | 26.32 | *CD300E* | *CD52, CEP85, CRYBG2, SH3BGRL3, UBXN11* | | 1 |
| 1 | 26.72 | 26.92 | *TNFRSF13B* | *ARID1A, GPATCH3, GPN2, NR0B2, NUDC, ...* | | 1 |
| 1 | 38.15 | 38.18 | *TNFRSF17* | | | 1 |
| 1 | 38.82 | 38.91 | *CD5L* | *GJA9, MYCBP, RHBDL2, RRAGC* | | 1 |
| 1 | 43.33 | 44.12 | *PDCD1* | *ARTN, ATP6V0B, B4GALT2, CCDC24, CDC20, ...* | | 1 |
| 1 | 45.33 | 45.56 | *CD5L, TNFRSF9* | *AKR1A1, CCDC163, HMGB1P48, MMACHC, MUTYH, ...* | | 2 |
| 1 | 47.41 | 47.49 | *SHISA5* | *FOXD2, FOXE3, LINC01389* | | 1 |
| 1 | 54.38 | 54.40 | *SIGLEC1* | *SSBP3* | | 1 |
| 1 | 66.90 | 67.04 | *CD300E* | *DNAI4, MIER1, SLC35D1* | | 1 |
| 1 | 99.33 | 99.38 | *FCRL5* | | | 1 |
| 1 | 100.53 | 100.72 | *CD5L* | *GPR88, LINC01349, RPL7AP17, VCAM1* | | 1 |
| 1 | 109.24 | 109.30 | *LAG3* | *CELSR2, MYBPHL, PSRC1* | | 1 |
| 1 | 113.53 | 114.13 | *CD5L, CRTAM, CXCL10, PDCD1* | ***PTPN22***, *AP4B1, BCL2L15, DCLRE1B, HIPK1, ...* | | 4 |
| 1 | 117.64 | 117.66 | *CD27* | *VDAC2P3* | | 1 |
| 1 | 150.29 | 150.98 | *CD300E, TNFRSF17* | *ADAMTSL4, ARNT, CERS2, CIART, CTSK, ...* | | 2 |
| 1 | 152.75 | 153.13 | *SLAMF7* | *IVL, KPRP, LCE1A, LCE1B, ...* | | 1 |
| 1 | 154.68 | 154.96 | *SLAMF7* | *KCNN3, Metazoa SRP, PBXIP1, PMVK, ...* | *ADAR1* | 1 |
| 1 | 156.77 | 156.84 | *PDCD1* | *HDGF, NTRK1, PRCC, SH2D2A* | | 1 |
| 1 | 161.19 | 161.22 | *CD300E* | *ADAMTS4, FCER1G, NDUFS2* | | 1 |
| 1 | 167.80 | 167.81 | *SLAMF7* | | | 1 |
| 1 | 173.34 | 173.48 | *CD5L* | ***TNFSF4, LOC100506023, PRDX6*** | | 1 |

| | | | | | | |
|---|---|---|---|---|---|---|
| 1 | 178.99 | 179.79 | *CD27, PDCD1* | *ABL2, AXDND1, COX5BP8, EIF4A1P11, FAM163A, ...* | | 2 |
| 1 | 184.70 | 184.90 | *CD300E* | ***EDEM3***, *NIBAN1* | | 1 |
| 1 | 196.53 | 197.34 | *ALKBH2, CXCL13, IL5RA, LGALS2, MX1* | *ASPM, CFH, CFHR1, CFHR2, CFHR3, ...* | | 5 |
| 1 | 198.30 | 198.46 | *PDCD1* | ***PTPRC***, ***CD45***, *, NEK7* | | 1 |
| 1 | 199.93 | 200.02 | *SIGLEC1* | | | 1 |
| 1 | 206.50 | 206.51 | *PDCD1, TNFRSF9* | ***IKBKE***, *C1orf147, RASSF5* | | 2 |
| 1 | 212.85 | 212.92 | *MX1* | *FLVCR1, SPATA45* | | 1 |
| 1 | 226.72 | 226.90 | *LAG3, TNFRSF17* | *ITPKB, PSEN2, RPS27P5* | | 2 |
| 2 | 3.59 | 3.60 | *LGALS2, ZG16* | *COLEC11* | | 2 |
| 2 | 20.13 | 20.19 | *TNFRSF17* | *RN7SL140P, RPS16P2* | | 1 |
| 2 | 25.63 | 25.93 | *IL5RA* | *ASXL2, DTNB, KIF3C, NDUFB4P4, PTGES3P2, ...* | | 1 |
| 2 | 27.36 | 27.64 | *CD27, CXCL10, SIGLEC1* | *C2orf16, CCDC121, EIF2B4, FNDC4, FTH1P3, ...* | | 3 |
| 2 | 30.20 | 30.23 | *LAG3* | ***LBH***, *H3P5* | | 1 |
| 2 | 33.48 | 33.48 | *TNFRSF17* | ***RASGRP3*** | | 1 |
| 2 | 38.69 | 38.82 | *CRTAM* | *ASS1P2, DHX57, GALM, GEMIN6, NPLP1, ...* | | 1 |
| 2 | 42.08 | 42.11 | *CD27* | | | 1 |
| 2 | 43.22 | 43.64 | *TNFRSF9* | *LINC01126, RN7SL531P, THADA, Y RNA, ...* | | 1 |
| 2 | 60.32 | 60.36 | *SLAMF7* | *MIR4432HG* | | 1 |
| 2 | 62.21 | 62.38 | *PDCD1* | *B3GNT2, RN7SL51P* | | 1 |
| 2 | 71.83 | 71.83 | *SLAMF7* | | | 1 |
| 2 | 72.13 | 72.13 | *FCRL5* | *CYP26B1* | | 1 |
| 2 | 88.57 | 88.63 | *FCRL5* | *EIF2AK3* | | 1 |
| 2 | 96.58 | 96.78 | *TNFRSF13B* | *CNNM4, FER1L5, KANSL3, LMAN2L* | | 1 |
| 2 | 100.09 | 100.25 | *FCRL5, TNFRSF17* | *AFF3, LINC01104* | | 2 |
| 2 | 102.28 | 102.48 | *ALKBH2, CXCL10* | *IL18R1, IL18RAP, IL1RL1, MIR4772, ...* | | 2 |
| 2 | 105.65 | 105.84 | *CD5L* | *NCK2* | | 1 |
| 2 | 110.83 | 111.25 | *CD300E, CD5L, CRTAM, IL5RA, SLAMF7* | ***BCL2L11***, *ACOXL, RPL5P9* | | 5 |
| 2 | 133.71 | 134.97 | *SIGLEC1* | *ACMSD, CCNT2, EDDM3CP, MAP3K19, MGAT5, ...* | | 1 |
| 2 | 135.84 | 136.16 | *SIGLEC1* | ***DARS***, *CXCR4, DARS1, MANEALP1, MCM6* | | 1 |
| 2 | 159.47 | 159.82 | *FCRL5, TNFRSF17* | *BAZ2B, CD302, LY75, MARCHF7, RPS3AP13, ...* | | 2 |
| 2 | 162.11 | 162.47 | *CXCL10, MX1, SIGLEC1* | ***FAP***, ***IFIH1***, *GCA, GCG, KCNH7* | *IFIH1* | 3 |
| 2 | 190.64 | 191.14 | *CRTAM, CXCL10, PDCD1* | ***NEMP2***, ***NAB1***, ***STAT4***, *GLS, RAB1AP1, ...* | | 3 |

| | | | | | | |
|---|---|---|---|---|---|---|
| 2 | 202.36 | 202.78 | *SIGLEC1* | *BMPR2, FAM117B, H3P8, ICA1L, MTATP6P17, ...* | | 1 |
| 2 | 203.83 | 203.93 | *CD27, PDCD1, TNFRSF17* | ***CTLA4*** | | 3 |
| 2 | 233.23 | 233.47 | *CD27* | *ATG16L1, DGKD, SAG, SCARNA5, SCARNA6, ...* | | 1 |
| 3 | 4.58 | 4.58 | *CD27* | *ITPR1* | | 1 |
| 3 | 27.72 | 27.76 | *FCRL5* | *EOMES* | | 1 |
| 3 | 32.39 | 32.46 | *CD5L* | *CMTM7* | | 1 |
| 3 | 45.87 | 46.53 | *CXCL10* | *CCR1, CCR2, CCR3, CCR5, CCR5AS, ...* | | 1 |
| 3 | 46.85 | 47.54 | *CD300E, CRTAM, TNFRSF9* | *BOLA2P2, CCDC12, ELP6, KIF9, KLHL18, ...* | | 3 |
| 3 | 48.63 | 50.38 | *TNFRSF17* | *ACTL11P, AMIGO3, AMT, APEH, ARIH2, ...* | *TREX1* | 1 |
| 3 | 56.83 | 56.92 | *CD27, PDCD1* | *ARHGEF3* | | 2 |
| 3 | 101.97 | 102.09 | *CD5L* | *LINC02085* | | 1 |
| 3 | 119.39 | 119.54 | *TNFRSF13B* | ***TMEM39A**, **TIMMDC1**, **TMEM39A1**, **CD80**, ARHGAP31, ...* | | 1 |
| 3 | 127.76 | 127.76 | *CD5L* | *MGLL* | | 1 |
| 3 | 128.57 | 128.68 | *CD300E* | *LINC01565, Metazoa SRP, POU5F1P6, RPN1* | | 1 |
| 3 | 130.93 | 131.40 | *LGALS2* | *ASTE1, ATP2C1, NEK11, NUDT16, NUDT16L2P* | | 1 |
| 3 | 152.20 | 152.53 | *CD5L, FCRL5* | *MBNL1, TMEM14EP, Y RNA* | | 2 |
| 3 | 157.09 | 157.10 | *FCRL5* | *LINC00880, LINC00881* | | 1 |
| 3 | 166.05 | 166.14 | *LGALS2* | | | 1 |
| 3 | 170.00 | 170.20 | *PDCD1* | *GPR160, KMT5AP3, PHC3* | | 1 |
| 3 | 176.98 | 177.15 | *FCRL5* | *MTND5P15, TBL1XR1, Y RNA* | | 1 |
| 3 | 186.99 | 187.07 | *FCRL5, PDCD1, SLAMF7* | *ST6GAL1* | | 3 |
| 3 | 192.90 | 192.91 | *TNFRSF13B, TNFRSF17* | *MB21D2* | | 2 |
| 3 | 196.26 | 196.58 | *CD5L, IL5RA* | *DYNLT2B, FBXO45, PCYT1A, RN7SL434P, RN7SL738P, ...* | | 2 |
| 4 | 0.79 | 1.42 | *CD5L, SIGLEC1, TNFRSF17* | ***DGKQ**, CPLX1, CTBP1, FGFRL1, GAK, ...* | | 3 |
| 4 | 38.72 | 38.91 | *FCRL5* | *FAM114A1, MIR574, RNA5SP158, TLR1, TLR10, ...* | | 1 |
| 4 | 54.60 | 54.65 | *CD300E* | ***KIT**, LINC02260* | | 1 |
| 4 | 67.61 | 67.75 | *CD5L* | *GNRHR, SNORA62, ST3GAL1P1, UBA6* | | 1 |
| 4 | 76.26 | 76.52 | *SHISA5* | *CCDC158, FAM47E, SHROOM3, SNX5P1, STBD1* | | 1 |
| 4 | 83.22 | 83.27 | *CD300E* | + | | 1 |
| 4 | 98.96 | 99.17 | *SIGLEC1* | *ABT1P1, ADH4, ADH5, METAP1, MIR3684, ...* | | 1 |

| | | | | | | |
|---|---|---|---|---|---|---|
| 4 | 101.17 | 101.60 | *CD5L, TNFRSF17* | ***BANK1***, *FLJ20021, MIR1255A, MIR8066, PPP3CA* | | 2 |
| 4 | 102.47 | 103.22 | *CD5L, IL5RA, TNFRSF17* | *ACTR3BP4, BDH2, CENPE, CISD2, KRT8P46, ...* | | 3 |
| 4 | 104.58 | 104.92 | *CD300E* | *RPL6P14* | | 1 |
| 4 | 121.25 | 121.26 | *CXCL10* | | | 1 |
| 4 | 142.71 | 142.86 | *TNFRSF9* | *INPP4B* | | 1 |
| 4 | 145.96 | 146.09 | *FCRL5, IL5RA* | *LINC01095, Y RNA* | | 2 |
| 5 | 1.28 | 1.29 | *TNFRSF17* | ***TERT*** | | 1 |
| 5 | 35.80 | 35.93 | *PDCD1* | ***IL7R***, ***CAPSL***, *SPEF2* | | 1 |
| 5 | 39.36 | 39.39 | *SHISA5* | *C9, DAB2* | | 1 |
| 5 | 56.14 | 56.27 | *CD300E* | *ANKRD55, C1GALT1P2, RNA5SP184, RNA5SP185* | | 1 |
| 5 | 58.47 | 58.51 | *CD5L* | *GAPT* | | 1 |
| 5 | 69.14 | 69.53 | *LAG3* | *AK6, CCDC125, CCNB1, CDK7, CENPH, ...* | | 1 |
| 5 | 70.87 | 71.75 | *LAG3* | *BDP1, CARTPT, CDH12P4, GTF2H2, GUSBP17, ...* | | 1 |
| 5 | 72.47 | 72.65 | *FCRL5* | *LINC02056, ZNF366* | | 1 |
| 5 | 89.38 | 89.46 | *CD5L* | | | 1 |
| 5 | 95.89 | 96.01 | *TNFRSF17* | *ELL2, FABP5P5* | | 1 |
| 5 | 125.00 | 125.02 | *CD5L* | *LINC02240* | | 1 |
| 5 | 126.88 | 127.21 | *CD27* | *C5orf63, MARCHF3, MRPS5P3, SELENOTP2* | | 1 |
| 5 | 130.72 | 131.90 | *LAG3* | ***FNIP1***, *ACTBP4, CDC42SE2, HINT1, LYRM7, ...* | | 1 |
| 5 | 132.15 | 132.68 | *CD27, CD300E, CRTAM, CXCL10, IL5RA, LAG3* | ***IRF1***, *, IL13, IL4, IL5, ...* | | 6 |
| 5 | 134.09 | 134.12 | *TNFRSF17* | ***TCF7***, ***SKP1*** | | 1 |
| 5 | 138.16 | 138.29 | *PDCD1* | *BRD8, CDC23, CDC25C, GFRA3, KIF20A, ...* | | 1 |
| 5 | 139.44 | 139.63 | *CXCL10, MX1* | *DNAJC18, ECSCR, H3P25, NCOA4P4, SMIM33, ...* | *STING, TMEM173* | 2 |
| 5 | 150.08 | 150.13 | *CD300E* | *CSF1R, PDGFRB, RPL7P1, Y RNA* | | 1 |
| 5 | 154.50 | 154.52 | *TNFRSF13B* | | | 1 |
| 5 | 177.13 | 177.39 | *SHISA5* | *LMAN2, MXD3, NSD1, PRELID1, ...* | | 1 |
| 6 | 0.18 | 0.98 | *CD5L, FCRL5, SLAMF7, TNFRSF17* | ***LOC285766***, ***DUSP22***, ***IRF4***, *EXOC2, HUS1B, ...* | | 4 |
| 6 | 6.73 | 6.76 | *TNFRSF9* | | | 1 |
| 6 | 7.08 | 7.09 | *IL5RA* | | | 1 |
| 6 | 7.32 | 7.32 | *CD27* | *SSR1* | | 1 |
| 6 | 41.71 | 41.71 | *CD5L* | *TFEB* | | 1 |
| 6 | 51.28 | 51.30 | *PDCD1* | | | 1 |
| 6 | 90.10 | 90.33 | *PDCD1, TNFRSF13B* | ***BACH2***, *MIR4464* | *BACH2* | 2 |

| | | | | | | |
|---|---|---|---|---|---|---|
| 6 | 106.12 | 106.43 | *TNFRSF13B, TNFRSF17* | ***PRDM1***, ***ATG5***, *CRYBG1, RN7SL47P, U4, ...* | | 2 |
| 6 | 116.45 | 116.50 | *LGALS2* | ***DSE***, *CALHM6, CBX3P9, KRT18P22, TRAPPC3L* | | 1 |
| 6 | 117.68 | 117.73 | *TNFRSF17* | *NUS1* | | 1 |
| 6 | 137.64 | 137.92 | *CD5L, TNFRSF9* | ***OLIG3***, ***LOC100130476***, ***TNFAIP3***, ***PERP***, , ... | *TNFAIP3* | 2 |
| 6 | 139.30 | 139.31 | *IL5RA* | | | 1 |
| 6 | 142.85 | 142.86 | *IL5RA* | *HIVEP2* | | 1 |
| 6 | 144.69 | 144.72 | *TNFRSF17* | *UTRN* | | 1 |
| 6 | 149.24 | 149.48 | *FCRL5* | *FABP12P1, RN7SL234P, SUMO4, TAB2, ZC3H12D* | | 1 |
| 6 | 159.05 | 159.09 | *PDCD1* | | | 1 |
| 7 | 1.23 | 1.26 | *SHISA5, TNFRSF17* | *UNCX* | | 2 |
| 7 | 6.34 | 6.45 | *TNFRSF17* | *DAGLB, FAM220A, KDELR2, RAC1, RPSAP73, ...* | | 1 |
| 7 | 25.96 | 26.00 | *CD27* | | | 1 |
| 7 | 50.21 | 50.55 | *CD27, CD5L, CXCL13, FCRL5, IL5RA, LAG3, MX1, SIGLEC1, SLAMF7* | ***IKZF1***, ***C7orf72***, *DDC, FIGNL1* | *IKZF1* | 9 |
| 7 | 76.30 | 76.63 | *CD27* | *DTX2, FDPSP2, HSPB1, LINC03009, POMZP3, ...* | | 1 |
| 7 | 128.92 | 129.14 | *ALKBH2, CD27, CD300E, CXCL10, LAG3, LGALS2, SHISA5, SLAMF7, TNFRSF13B, TNFRSF17, ZG16* | ***IRF5***, ***TNPO3***, *CYCSP20, ODCP, RN7SL306P, ...* | | 11 |
| 7 | 135.00 | 135.24 | *CD5L* | *AGBL3, CYREN, MIR6509, RNF14P4, STRA8, ...* | | 1 |
| 7 | 140.02 | 140.06 | *SIGLEC1* | *PARP12, TBXAS1* | | 1 |
| 7 | 150.54 | 151.33 | *CD5L, IL5RA, TNFRSF13B* | *ABCB8, ABCF2, AGAP3, ALDH7A1P3, AOC1, ...* | | 3 |
| 7 | 151.71 | 151.72 | *SHISA5* | *PRKAG2* | | 1 |
| 8 | 10.88 | 11.61 | *CD5L, FCRL5* | ***MSRA***, ***BLK***, ***FAM167A***, ***C8orf13***, *ensembl , ...* | | 2 |
| 8 | 23.52 | 23.53 | *TNFRSF13B* | *SLC25A37* | | 1 |
| 8 | 23.84 | 23.93 | *CD27* | *STC1* | | 1 |
| 8 | 78.64 | 78.80 | *TNFRSF13B* | ***PKIA***, ***ZC2HC1A***, *C4orf46P3, IL7, THAP12P7* | | 1 |
| 8 | 102.52 | 102.62 | *CD5L, FCRL5, IL5RA* | *ODF1, POU5F1P2* | | 3 |
| 8 | 110.16 | 110.17 | *CRTAM* | | | 1 |
| 8 | 128.15 | 128.21 | *CD5L* | ***LINC00824***, *PVT1* | | 1 |
| 8 | 141.64 | 141.65 | *TNFRSF13B* | | | 1 |
| 8 | 143.90 | 144.04 | *FCRL5* | *GRINA, MIR661, PARP10, PLEC, SPATC1* | | 1 |
| 9 | 1.85 | 1.87 | *PDCD1* | | | 1 |
| 9 | 5.48 | 5.50 | *PDCD1* | | | 1 |
| 9 | 27.34 | 27.34 | *MX1* | *MOB3B* | | 1 |

| | | | | | | |
|---|---|---|---|---|---|---|
| 9 | 32.43 | 33.19 | *CXCL10, FCRL5, MX1, SIGLEC1, SLAMF7* | *ACO1, APTX, ASS1P12, B4GALT1, BOLA3P4, ...* | *RIGI (DDX58)* | 5 |
| 9 | 35.55 | 35.62 | *CD5L* | *CD72, FAM166B, MIR4667, RPS29P17, RUSC2, ...* | | 1 |
| 9 | 75.15 | 75.16 | *ZG16* | | | 1 |
| 9 | 87.46 | 87.52 | *IL5RA, SLAMF7* | *DAPK1* | | 2 |
| 9 | 93.07 | 93.09 | *TNFRSF13B* | *SUSD3* | | 1 |
| 9 | 110.57 | 110.60 | *CXCL10* | *SVEP1* | | 1 |
| 9 | 113.30 | 113.36 | *CD300E, SIGLEC1* | *BSPRY, WDR31* | | 2 |
| 9 | 114.57 | 114.94 | *CRTAM, SLAMF7, TNFRSF17* | *ATP6V1G1, DELEC1, TEX48, TEX53, TMEM268, ...* | | 3 |
| 9 | 120.75 | 120.96 | *PDCD1* | *B3GALT9, C5, CUTALP, FBXW2, PHF19, ...* | | 1 |
| 9 | 133.27 | 133.37 | *SIGLEC1* | *ABO, LCN1P2, MED22, RPL7A, SNORD24, ...* | | 1 |
| 9 | 136.35 | 136.49 | *LAG3, MX1* | *C9orf163, CARD9, DNLZ, ENTR1, GPSM1, ...* | | 2 |
| 10 | 0.82 | 1.13 | *SHISA5* | *GTPBP4, IDI1, IDI2, LARP4B, WDR37* | | 1 |
| 10 | 3.86 | 3.89 | *CD27, CD5L, TNFRSF17* | | | 3 |
| 10 | 5.69 | 6.15 | *PDCD1, SHISA5, SLAMF7, TNFRSF13B, TNFRSF17* | ***ANKRD16***, *FBH1, GDI2, IL15RA, IL2RA, ...* | | 5 |
| 10 | 8.04 | 8.07 | *TNFRSF17* | *GATA3* | | 1 |
| 10 | 11.10 | 11.21 | *FCRL5* | *CELF2* | | 1 |
| 10 | 11.92 | 12.05 | *CD27* | *UPF2* | | 1 |
| 10 | 28.49 | 28.68 | *TNFRSF17* | *BAMBI, LINC02652, RNU4ATAC6P, TPRKBP1, WAC* | | 1 |
| 10 | 30.40 | 30.49 | *SIGLEC1* | *CCND3P1, MAP3K8* | | 1 |
| 10 | 48.77 | 48.91 | *MX1, SIGLEC1* | ***WDFY4***, ***LRRC18*** | | 2 |
| 10 | 62.69 | 62.79 | *CD27* | ***ZNF365***, *ALDH7A1P4* | | 1 |
| 10 | 69.38 | 69.54 | *PDCD1* | *ATP5MC1P7, HK1, TACR2, TMEM256P1, TSPAN15* | | 1 |
| 10 | 80.28 | 80.52 | *CD5L, PDCD1* | *DYDC1, DYDC2, MAT1A, PRXL2A, TSPAN14, ...* | | 2 |
| 10 | 88.98 | 89.02 | *CD5L* | *ACTA2, FAS* | *FAS or TN-FRSF6* | 1 |
| 10 | 92.67 | 92.73 | *IL5RA* | *EIF2S2P3, HHEX, Y RNA* | | 1 |
| 10 | 110.20 | 110.87 | *CD5L, TNFRSF17* | ***BBIP1***, *DUSP5, HMGB3P5, MXI1, PDCD4, ...* | *SHOC2* | 2 |
| 10 | 132.93 | 132.99 | *CD5L* | *CFAP46, LINC01166, LINC01167, LINC01168* | | 1 |
| 11 | 9.73 | 9.76 | *TNFRSF13B* | *LINC02709, SWAP70* | | 1 |
| 11 | 47.26 | 47.55 | *CD5L* | *CELF1, MADD, MIR4487, MYBPC3, NR1H3, ...* | | 1 |
| 11 | 49.81 | 49.91 | *LGALS2* | *OR4A1P, TRIM51DP, TRIM51FP* | | 1 |

| | | | | | | |
|---|---|---|---|---|---|---|
| 11 | 60.35 | 60.41 | *LAG3* | *MS4A14, MS4A6E, MS4A7* | | 1 |
| 11 | 61.78 | 61.86 | *FCRL5* | *FADS1, FADS2, FEN1, MIR1908, MIR611, ...* | | 1 |
| 11 | 64.46 | 64.51 | *ALKBH2* | | | 1 |
| 11 | 65.53 | 65.79 | *IL5RA, TNFRSF17* | ***RNASEH2C***, ***OVOL1***, *AP5B1, EHBP1L1, FAM89B, ...* | *RNASEH2C* | 2 |
| 11 | 92.76 | 92.88 | *IL5RA* | *FAT3* | | 1 |
| 11 | 94.05 | 94.05 | *CXCL13* | *HEPHL1* | | 1 |
| 11 | 94.40 | 94.46 | *TNFRSF9* | *GPR83, MRE11* | | 1 |
| 11 | 102.34 | 102.39 | *SLAMF7* | *BIRC2* | | 1 |
| 11 | 111.40 | 111.41 | *SLAMF7* | *POU2AF1* | | 1 |
| 11 | 114.52 | 114.58 | *TNFRSF17* | *NXPE1, NXPE4* | | 1 |
| 11 | 118.63 | 118.84 | *FCRL5* | *+* | | 1 |
| 11 | 120.11 | 120.20 | *CD27* | *TRIM29* | | 1 |
| 11 | 122.63 | 122.72 | *CD5L* | *UBASH3B* | | 1 |
| 11 | 123.47 | 123.53 | *CD5L* | *GRAMD1B* | | 1 |
| 11 | 126.24 | 126.50 | *SIGLEC1* | *DCPS, FAM118B, FOXRED1, GSEC, KIRREL3, ...* | | 1 |
| 11 | 128.34 | 128.62 | *CRTAM, FCRL5, TNFRSF17* | ***ETS1***, ***FLI1***, *MIR6090* | | 3 |
| 12 | 6.38 | 6.41 | *CXCL13, SLAMF7, TNFRSF13B, TNFRSF9* | *LTBR* | | 4 |
| 12 | 7.44 | 7.57 | *TNFRSF13B* | *CD163, CD163L1, GAPDHP31* | *C1S, C1R* | 1 |
| 12 | 12.35 | 12.35 | *LAG3* | *MANSC1* | | 1 |
| 12 | 24.53 | 24.55 | *IL5RA* | *SOX5* | | 1 |
| 12 | 47.76 | 47.88 | *FCRL5, IL5RA, SLAMF7* | *HDAC7, RAPGEF3, SLC48A1, VDR* | | 3 |
| 12 | 56.47 | 56.50 | *SIGLEC1* | *GLS2, MIP, SPRYD4* | | 1 |
| 12 | 56.99 | 57.10 | *LAG3* | *GPR182, MYO1A, NAB2, NEMP1, STAT6, ...* | | 1 |
| 12 | 68.13 | 68.20 | *CXCL10* | *IFNG* | | 1 |
| 12 | 89.33 | 89.38 | *CD5L* | *DUSP6* | | 1 |
| 12 | 101.47 | 101.52 | *LAG3* | *RNA5SP367, SPIC* | | 1 |
| 12 | 109.89 | 112.80 | *CD27, CD300E, CD5L, CRTAM, CXCL10, CXCL13, FCRL5, IL5RA, LAG3, LGALS2, PDCD1, SHISA5, SIGLEC1, TNFRSF13B, TNFRSF9* | ***SH2B3***, ***ATXN2***, *ACAD10, ADAM1A, ADAM1B, ...* | *SH2B3, PTPN11* | 15 |
| 12 | 118.58 | 118.60 | *LAG3* | | | 1 |
| 12 | 120.70 | 120.94 | *PDCD1* | ***CABP1***, *ACADS, ARF1P2, CLIC1P1, MIR4700, ...* | | 1 |
| 12 | 124.36 | 124.38 | *SIGLEC1* | *NCOR2* | | 1 |
| 12 | 128.65 | 128.65 | *CD300E* | ***SLC15A4***, *TMEM132C* | | 1 |
| 13 | 27.94 | 28.07 | *CD300E* | *ATP5F1EP2, CDX2, FLT3, KATNBL1P1, LINC00543, ...* | | 1 |
| 13 | 107.44 | 107.50 | *TNFRSF13B* | *NALF1* | | 1 |
| 13 | 108.07 | 108.53 | *CD27, CD5L, FCRL5, TNFRSF13B, TNFRSF17, TNFRSF9* | *ABHD13, HCFC2P1, LIG4, RNA5SP39, TNFSF13B* | | 6 |
| 14 | 25.02 | 25.07 | *ZG16* | *STXBP6* | | 1 |
| 14 | 29.84 | 29.90 | *CD5L* | *PRKD1* | | 1 |

| | | | | | |
|---|---|---|---|---|---|
| 14 | 43.97 | 44.03 | *ZG16* | *LINC02307* | 1 |
| 14 | 67.50 | 67.51 | *SIGLEC1* | *TMEM229B* | 1 |
| 14 | 75.36 | 75.58 | *CD5L* | *BATF, FLVCR2, JDP2* | 1 |
| 14 | 102.55 | 103.09 | *CD27, CD5L, FCRL5, IL5RA, SLAMF7, TNFRSF13B, TNFRSF17* | ***TRAF3***, *AMN, CDC42BPB, LBHD2, LINC02323, ...* | 7 |
| 14 | 103.37 | 103.52 | *CD300E* | *MARK3, RPL10AP1, RPSAP5* | 1 |
| 14 | 105.40 | 105.74 | *FCRL5* | *ATP5MC1P1, COPDA1, CRIP1, CRIP2, ELK2AP, ...* | 1 |
| 14 | 106.62 | 106.82 | *ALKBH2* | *RNA5SP389, SLC20A1P1* | 1 |
| 15 | 43.33 | 44.14 | *LAG3* | *ACTBP7, ADAL, CATSPER2, CATSPER2P1, CKMT1A, ...* | 1 |
| 15 | 51.43 | 51.67 | *MX1* | *DMXL2* | 1 |
| 15 | 58.46 | 58.51 | *PDCD1* | *ALDH1A2, LIPC* | 1 |
| 15 | 69.69 | 69.88 | *CD5L, PDCD1* | *DRAIC, GEMIN8P1* | 2 |
| 15 | 70.08 | 70.09 | *FCRL5* | *TLE3* | 1 |
| 15 | 77.15 | 77.26 | *PDCD1* | *PEAK1* | 1 |
| 15 | 79.87 | 79.99 | *CD300E* | *BCL2A1, ensembl , MTHFS, ST20* | 1 |
| 15 | 84.85 | 84.86 | *TNFRSF9* | *ALPK3* | 1 |
| 15 | 90.39 | 90.47 | *CD5L* | *IQGAP1* | 1 |
| 15 | 94.26 | 94.28 | *CD5L* | *MCTP2* | 1 |
| 15 | 99.23 | 99.23 | *TNFRSF9* | *TTC23* | 1 |
| 16 | 8.00 | 8.00 | *TNFRSF13B* | | 1 |
| 16 | 8.48 | 8.49 | *SIGLEC1* | | 1 |
| 16 | 10.83 | 11.12 | *CD5L, PDCD1* | ***CLEC16A***, ***CIITA***, ***SOCS1***, *RPL7P46* | 2 |
| 16 | 11.88 | 12.03 | *CD27, CD5L* | *COX6CP1, GSPT1, NPIPB2, SNX29, TNFRSF17, ...* | 2 |
| 16 | 18.77 | 18.93 | *CD27* | *ARL6IP1, RPS15A, SMG1* | 1 |
| 16 | 20.34 | 20.40 | *CD27, CD300E, SHISA5, TNFRSF9* | *PDILT, UMOD* | 4 |
| 16 | 28.41 | 28.91 | *TNFRSF9* | *APOBR, ATP2A1, ATXN2L, CDC37P1, CDC37P2, ...* | 1 |
| 16 | 30.51 | 31.37 | *CD5L, IL5RA, SIGLEC1, SLAMF7* | ***ZNF629***, ***ITGAM***, ***ITGAX***, *BCKDK, BCL7C, ...* | 4 |
| 16 | 66.40 | 66.50 | *CD5L* | *BEAN1, CDH5, LINC00920* | 1 |
| 16 | 67.15 | 68.38 | *TNFRSF17* | ***ZFP90***, *ACD, AGRP, ATP6V0D1, B3GNT9, ...* | 1 |
| 16 | 71.39 | 72.20 | *CD5L* | *AP1G1, ATP5F1AP3, ATXN1L, CHST4, DHODH, ...* | 1 |
| 16 | 85.90 | 85.98 | *CD300E* | ***IRF8***, *, LINC02132, MIR6774* | 1 |
| 16 | 89.64 | 89.85 | *SIGLEC1* | *CDK10, CHMP1A, FANCA, LINC02166, SPATA2L, ...* | 1 |

| | | | | | | |
|---|---|---|---|---|---|---|
| 17 | 7.03 | 7.79 | *CD27, CD5L, CRTAM, FCRL5, IL5RA, LAG3, PDCD1, SIGLEC1, SLAMF7, TNFRSF17* | ***NEURL4***, ***ACAP1***, *ACADVL, ASGR1, ASGR2, ...* | | 10 |
| 17 | 16.59 | 17.23 | *CD27, CD5L, FCRL5, IL5RA, SLAMF7, TNFRSF17, TNFRSF9* | ***TNFRSF13B***, *CCDC144A, COTL1P1, FAM106C, FLCN, ...* | | 7 |
| 17 | 27.44 | 27.55 | *CD300E* | *KSR1, MSANTD3P1* | | 1 |
| 17 | 28.27 | 28.39 | *LGALS2* | *IFT20, KRT18P55, MIR4723, POLDIP2, ...* | | 1 |
| 17 | 35.44 | 35.49 | *CXCL10* | *E2F3P1, SLFN12L, SLFN13* | | 1 |
| 17 | 39.64 | 40.21 | *CD300E, CD5L, FCRL5, IL5RA, SIGLEC1, TNFRSF13B, TNFRSF17, TNFRSF9* | ***ERBB2***, ***MIEN1***, ***GRB7***, ***GSDMB***, ***IKZF3***, ***HER-2***, ***C17orf37***, ***ZNFN1A3***, ***ZBPB2***, *...* | | 8 |
| 17 | 42.31 | 42.41 | *IL5RA* | *CAVIN1, STAT3* | | 1 |
| 17 | 45.16 | 46.79 | *CD27, CD5L, CRTAM, FCRL5, TNFRSF13B, TNFRSF17* | *ARHGAP27, ARL17A, ARL17B, CRHR1, DND1P1, ...* | | 6 |
| 17 | 47.95 | 48.47 | *CD27* | *CBX1, CDK5RAP3, COPZ2, MIR1203, MIR152, ...* | | 1 |
| 17 | 49.24 | 49.40 | *PDCD1* | *FLJ40194, MIR6129, ZNF652* | | 1 |
| 17 | 59.48 | 59.98 | *SIGLEC1* | *CLTC, DHX40, DHX40P1, LINC01476, MIR21, ...* | | 1 |
| 17 | 68.49 | 69.53 | *CXCL13, LAG3* | *ABCA10, ABCA5, ABCA6, ABCA8, ABCA9, ...* | | 2 |
| 17 | 70.22 | 70.26 | *CD27* | *CALM2P1* | | 1 |
| 17 | 72.75 | 72.75 | *TNFRSF13B* | *SLC39A11* | | 1 |
| 17 | 76.67 | 76.85 | *SLAMF7* | *JMJD6, LINC02080, METTL23, MFSD11, ...* | | 1 |
| 17 | 79.93 | 79.94 | *FCRL5, TNFRSF17* | *TBC1D16* | | 2 |
| 17 | 81.06 | 81.30 | *FCRL5, PDCD1, SLAMF7* | *AATK, BAIAP2, CEP131, MIR1250, MIR3065, ...* | | 3 |
| 18 | 12.77 | 12.88 | *CXCL10* | *LINC01882, PTPN2* | | 1 |
| 18 | 36.94 | 37.00 | *ALKBH2* | *KIAA1328* | | 1 |
| 18 | 55.05 | 55.38 | *CD27, CD5L* | *LINC01929, RNA5SP459, TCF4* | | 2 |
| 18 | 63.05 | 63.18 | *FCRL5, TNFRSF17* | *BCL2* | | 2 |
| 19 | 0.82 | 0.83 | *SLAMF7* | *+* | | 1 |
| 19 | 1.62 | 1.65 | *CD5L* | *TCF3* | | 1 |
| 19 | 3.46 | 3.55 | *TNFRSF17* | *C19orf71, DOHH, FZR1, MFSD12, NFIC, ...* | | 1 |
| 19 | 3.94 | 4.12 | *PDCD1* | *DAPK3, EEF2, MAP2K2, MIR637, NMRK2, ...* | | 1 |
| 19 | 6.53 | 6.55 | *TNFRSF9* | *+* | *C3* | 1 |
| 19 | 10.29 | 11.08 | *CXCL10, PDCD1* | ***TYK2***, *AP1M2, ATG4D, C19orf38, CARM1, ...* | | 2 |
| 19 | 16.30 | 16.64 | *CRTAM, FCRL5, TNFRSF17* | ***KLF2***, *C19orf44, CALR3, CHERP, EPS15L1, ...* | | 3 |
| 19 | 17.52 | 17.58 | *CD5L* | *COLGALT1, NIBAN3* | | 1 |

| | | | | | |
|---|---|---|---|---|---|
| 19 | 18.04 | 18.30 | *SIGLEC1* | ***LRRC25***, ***SSBP4***, , *IFI30, IL12RB1, ...* | 1 |
| 19 | 19.14 | 19.19 | *IL5RA, TNFRSF17* | *BORCS8, MEF2B, RFXANK* | 2 |
| 19 | 35.66 | 35.94 | *CD27* | *APLP1, ARHGAP33, HCST, HSPB6, IGFLR1, ...* | 1 |
| 19 | 42.03 | 42.25 | *IL5RA* | *DEDD2, ERF, GRIK5, GSK3A, MIR4323, ...* | 1 |
| 19 | 43.50 | 43.66 | *CD300E* | *CADM4, ETHE1, IRGQ, PHLDB3, PINLYP, ...* | 1 |
| 19 | 44.74 | 44.92 | *CD27, CD5L, CXCL10, CXCL13* | *APOC1, APOE, BCL3, NECTIN2, TOMM40* | 4 |
| 19 | 46.65 | 46.77 | *SIGLEC1* | *DACT3, FKRP, MIR320E, PRKD2, RN7SL364P, ...* | 1 |
| 19 | 48.59 | 48.75 | *FCRL5, PDCD1* | *FUT1, FUT2, IZUMO1, MAMSTR, NTN5, ...* | 2 |
| 19 | 54.15 | 54.27 | *TNFRSF13B* | *CNOT3, LENG1, LILRA6, LILRB3, LILRB5, ...* | 1 |
| 19 | 54.82 | 54.88 | *CD5L* | *FCAR, KIR2DS4, KIR3DL1, KIR3DL2* | 1 |
| 20 | 3.57 | 3.76 | *CD5L* | *ADAM33, ATRN, C20orf27, GFRA4, HSPA12B, ...* | 1 |
| 20 | 4.98 | 5.01 | *IL5RA, TNFRSF13B* | *SLC23A2* | 2 |
| 20 | 33.88 | 34.14 | *FCRL5* | *EIF2S2, MIR4755, PIGPP3, RALY, RPS2P1, ...* | 1 |
| 20 | 34.99 | 35.20 | *CD300E* | *EDEM2, MIR499A, MIR499B, MYH7B, PROCR, ...* | 1 |
| 20 | 40.87 | 40.89 | *SLAMF7* | | 1 |
| 21 | 29.54 | 29.64 | *TNFRSF13B* | *BACH1, GRIK1* | 1 |
| 21 | 35.05 | 35.13 | *TNFRSF9* | *RUNX1* | 1 |
| 21 | 44.29 | 44.29 | *PDCD1* | *AIRE* | 1 |
| 22 | 21.56 | 21.64 | *CD27, TNFRSF17, TNFRSF9* | ***UBE2L3***, ***HIC2***, ***YDJC***, *CCDC116, SDF2L1* | 3 |
| 22 | 23.75 | 23.84 | *IL5RA, TNFRSF17* | *C22orf15, CHCHD10, DERL3, MMP11, SMARCB1, ...* | 2 |
| 22 | 25.24 | 25.24 | *LAG3* | | 1 |
| 22 | 29.49 | 30.21 | *CXCL13, FCRL5, SLAMF7, TNFRSF13B* | *ASCC2, CABP7, CNN2P1, HORMAD2, MIR6818, ...* | 4 |
| 22 | 36.36 | 36.46 | *CD27* | *MYH9, RPS15AP38, Y RNA* | 1 |
| 22 | 39.02 | 39.02 | *SLAMF7* | *APOBEC3C, APOBEC3D, ensembl* | 1 |
| 22 | 40.80 | 42.41 | *PDCD1, TNFRSF13B, TNFRSF9* | *ACO2, ACTBP15, C22orf46, CCDC134, CENPM, ...* | 3 |
| 22 | 43.12 | 43.17 | *CD300E* | *BIK, MCAT, TSPO, TTLL12* | 1 |

For each clump that harbors *trans*-QTLs for a target gene, a locus-specific score for that clump and that gene is computed. The GATE score for each gene is then computed by adding up these locus-specific scores across all clumps. Genes within 200kb from the clump are in bold if they have been previously reported as SLE GWAS hits. Only the first 5 genes are listed for each clump, and three dots are denote the presence of more than 5 genes in that location. If rare SNPs within 200kb from the clump were associated with the monogenic Lupus, we report the corresponding gene. A single dot denotes a clump which was not annotated by genes in Ensembl.
